# Animal Ca^2+ ^release-activated Ca^2+ ^(CRAC) channels appear to be homologous to and derived from the ubiquitous cation diffusion facilitators

**DOI:** 10.1186/1756-0500-3-158

**Published:** 2010-06-03

**Authors:** Madeleine G Matias, Kenny M Gomolplitinant, Dorjee G Tamang, Milton H Saier

**Affiliations:** 1 Division of Biological Sciences, University of California at San Diego, La Jolla, California 92093-0116, USA

## Abstract

**Background:**

Antigen stimulation of immune cells triggers Ca^2+ ^entry through Ca^2+ ^release-activated Ca^2+ ^(CRAC) channels, promoting an immune response to pathogens. Defects in a CRAC (Orai) channel in humans gives rise to the hereditary Severe Combined Immune Deficiency (SCID) syndrome. We here report results that define the evolutionary relationship of the CRAC channel proteins of animals, and the ubiquitous Cation Diffusion Facilitator (CDF) carrier proteins.

**Findings:**

CDF antiporters derived from a primordial 2 transmembrane spanner (TMS) hairpin structure by intragenic triplication to yield 6 TMS proteins. Four programs (IC/GAP, GGSEARCH, HMMER and SAM) were evaluated for identifying sequence similarity and establishing homology using statistical means. Overall, the order of sensitivity (similarity detection) was IC/GAP = GGSEARCH > HMMER > SAM, but the use of all four programs was superior to the use of any two or three of them. Members of the CDF family appeared to be homologous to members of the 4 TMS Orai channel proteins.

**Conclusions:**

CRAC channels derived from CDF carriers by loss of the first two TMSs of the latter. Based on statistical analyses with multiple programs, TMSs 3-6 in CDF carriers are homologous to TMSs 1-4 in CRAC channels, and the former was the precursor of the latter. This is an unusual example of how a functionally and structurally more complex protein may have predated a simpler one.

## Background

Antigen stimulation of immune cells triggers Ca^2+ ^entry through Ca^2+ ^release-activated Ca^2+ ^(CRAC) channels, promoting an immune response to pathogens [1]. Cells from patients with one form of the hereditary Severe Combined Immune Deficiency (SCID) syndrome are defective in Store-Operated Ca^2+ ^(SOC) entry and CRAC channel function [2]. The genetic defect in these patients appears to be in a protein called Orai1, which contains four putative transmembrane segments (TMSs) [3]. SCID patients are homozygous for a single missense mutation in Orai1 (TC# 1.A.52.1.1), and expression of wild-type Orai1 in SCID T cells restores SOC influx and the CRAC current. Orai1 is an essential component of the CRAC channel complex [4,5].

Human Orai1 has homologues in all animals with sequenced genomes, and these channel proteins have been identified largely in animals. They interact with Stromal Interaction Molecule 1 (STIM1) to form the functional channel complex [5-8]. One study concluded that Orai1 forms a homotetramer [9]. Coupling of STIM1 to SOC entry depends on its movement in the endoplasmic reticulum (ER) [10].

Orai1 and TRPC1 are core components of CRAC and SOC channels, respectively [3,11]. Mutations of acidic residues in TMSs 1 and 3 and in the I-II loop of Orai1 decrease Ca^2+ ^flux and increase Cs^+ ^flux [12]. STIM1, a Ca^2+^-sensor of luminal Ca^2+ ^content in the ER, interacts with and mediates store-dependent regulation of both channels. TRPC1^+ ^Stim1-dependent SOC requires functional Orai1 [13]. Stim1 triggers activation of CRAC channels in the surface membrane after Ca^2+ ^store depletion [11,14].

Although CRAC channels have been characterized only from animals, homologues may be present in unicellular eukaryotes such as the choanoflagellates [15]. A limited distribution in eukaryotes is implied. However, CDF antiporters are ubiquitous, being found in profusion in bacteria, archaea and eukaryotes [16]. They transport heavy metals including cobalt, cadmium, zinc and possibly nickel, copper and mercuric ions. There are 10 mammalian CDF paralogues [17].

Most members of the CDF family possess six putative transmembrane spanners with N- and C-termini on the cytoplasmic side of the membrane [18]. These proteins exhibit an unusual degree of sequence divergence and size variation (300-750 residues), and eukaryotic proteins exhibit differences in cell localization. Some (e.g., ZnT2-7) catalyze heavy metal uptake from the cytoplasm into various intracellular organelles while others (e.g., ZnT1) catalyze efflux from the cytoplasm across the plasma membrane into the extracellular medium [19-21].

At least two metal binding residues have been identified in the *E. coli *homologue, YiiP (TC #2.A.4.1.5), and one plays a role in H^+ ^binding as well [19]. The two Zn^2+^/Cd^2+ ^binding residues are two interacting conserved aspartyl residues (Asp-157 and Asp-49) at the dimer interface of the homodimer [22]. The β-carboxyl groups in these two residues were suggested to form a bimetal binding center [21-23].

Lu and Fu [24] have reported the x-ray structure of YiiP in complex with zinc at 3.8 angstrom resolution. YiiP is a homodimer held together in a parallel orientation through four Zn^2+ ^ions at the interface of the cytoplasmic domains. The two transmembrane domains swing out to yield a Y-shaped structure. In each protomer, the cytoplasmic domain adopts a metallochaperone-like protein fold. The transmembrane domain features a bundle of six transmembrane helices and a tetrahedral Zn^2+ ^binding site located in a cavity that is open to the membrane outer leaflet and the periplasm. The generalized transport reaction for CDF family members involves heavy metal:H^+ ^antiport.

## Methods

### Supplementary Materials

All supplementary materials for this paper can be found at the following web address: http://www.biology.ucsd.edu/~msaier/supmat/Crac/index.html

### Similarity Searches & Construction of Phylogenetic Trees

PSI-Blast [25] searches were performed to screen the National Center for Biotechnology Information (NCBI) non-redundant (nr) protein database using *Homo sapiens *Orai1 (TC# 2.A.52.1.1; gi# 97180269), *H. sapiens *Stim1 (TC# 1.A.52.1.1; gi# 17368447) and the *Bacillus subtilis *CDF antiporter, CzcD (TC# 2.A.4.1.3; gi# 16079718) as query sequences. Protein sequence alignments were performed using ClustalX version 1.83 [26]. Redundant and partial sequences were removed so that only unique, full length, representative Orai, Stim and CDF homologues were analyzed further. For this purpose, a modified CD-HIT program [27,28] was used; for Orai proteins, the cutoff point was 90% sequence identity, while for CDF sequences, it was 50%. Multiple alignment files adjusted by ClustalX [26] were exported to files in Clustal format. The TREEVIEW program [29] was used to display the phylogenetic trees.

### Establishment of Homology

To establish homology (common ancestry), either between two proteins or between two internal segments in a set of homologous proteins, the IC and GAP programs were initially used (our gold standard) [30-32]. For establishing homology among putative full-length homologues, or repeat sequences of greater than sixty amino acyl residues, a value of 9 - 10 S.D. is considered sufficient [33,34]. According to [35], 9 standard deviations corresponds to a probability of 10^-19 ^that this degree of similarity arose by chance, and 10 S.D. corresponds to a probability of 10^-24^.

The GAP program produces a binary alignment, randomizes the two input sequences, and then compares the native alignment with 100 randomly shuffled alignments. We run this program five times and average the results, which IC does automatically [28]. Quality as well as average quality, based on 100 randomizations (± standard deviations) is presented in the output file. The standard deviation values reported in this and other papers from our laboratory are designated SD units by the GAP program and are generated using the equation: SD_Units = (quality - average_quality)/standard_deviation (the number given after the ±). "SD units" are also called standardized scores or Z scores. They are frequently used to compare scores produced by different methods because they are independent of the scoring system. One can use Z scores to compare results from different programs even though the absolute scores obtained with these programs are on completely different scales.

As will be shown in the results section, comparison of Orai channel proteins with CDF carrier proteins gave a maximal comparison score of 14.6 S.D., a value much greater than required to establish homology [33,34]. As a negative control, the three Orai (1-3) paralogues of *H. sapiens *(TC# 1.A.52) were run against several 4 TMS homologues of TWIK-1 (TC# 1.A.1.8.1) obtained using the NCBI BLAST search tool. The comparison scores resulting were low, between -1 and 5.5 S.D. Nothing above 5.6 S.D. was obtained. This control provides further evidence that comparison scores reported (up to 14.6 S.D.) are highly significant.

The two proteins (or sets of domains) to be compared were subjected to PSI-BLAST searches of the NCBI non-redundant protein database with a second iteration [28] (criteria as described below). These criteria have successfully been used to demonstrate internal repeats within dozens of transport protein families (see [36] for a review). In no case have the conclusions obtained using these methods been shown to be in error.

We have found that using a cut-off value of e^-3 ^for the initial BLAST search, and a cut-off value of e^-4 ^for the second iteration, we reliably retrieve homologues with very few false positives. Nevertheless, all retrieved proteins giving e-values of e^-5 ^or larger were tested for homology using the GAP program with default settings, requiring a comparison score of at least 9-10 S.D. in order to conclude that these proteins share a common origin. All hits that satisfied these criteria were put through a modified CD-HIT program with 90% cut-off [27,28] to eliminate redundancies, fragmentary sequences, and sequences with similarities of >90% identity. A multiple alignment was generated with the ClustalX program [26], and homology of all aligned sequences throughout the relevant transmembrane domains was established using the IC and GAP programs [31,32]. Internal regions to be examined for repeats were excised from the full-length protein sequences based on the multiple alignment as described in Zhou *et al*. [31], and dissimilar sets of segments were compared with potentially homologous regions of the same proteins using the IC and GAP programs with default settings and 500 random shuffles.

### Derivation of Consensus Sequences

To derive consensus sequences for the members of both the Orai and CDF families, the HMMER package http://hmmer.janelia.org; [37]) was used. All sequences of both families included in these studies were used to derive the consensus sequences. The hmmbuild program was used to align the sequences and build the model. Then hmmemit was used to generate the consensus sequence for each family.

### Comparison of Programs for Homology Estimation

More extensive evidence for homology was obtained by comparing four distinct programs, (1) the IC/GAP program set described above, (2) GGSEARCH, (3) HMMER2 and 3, and (4) SAM [28,38]. HMMER2 and 3 gave similar e-values. The use of these last three programs (2-4) was as follows:

### HMMER2 [39-41]

A single sequence (Protein-2) was used to retrieve homologous target sequences to be used to screen the HMM profile generated with a similar NCBI-BLASTP search where Protein-1 was the query sequence. The reverse procedure was used where Protein-1 was used to retrieve target sequences while Protein-2 was used to generate the profile. NCBI-BLASTP searches against the nr protein database were used with a cutoff e-value of 0.001. The homologous sequences in FASTA format were checked for redundancies, fragments, and nearly identical sequences which were eliminated with a 90% identity cutoff value using a modified CD-HIT program [28]. The remaining sequences were aligned with ClustalX. The hmmbuild program was used to build the profile HMM. The profile was then calibrated with hmmcalibrate for more accurate e-values. The sequence (FASTA) file of the other protein (Protein-1) was then searched with the resulting HMM profile. hmmsearch was used to search the target sequence database, resulting in an output file with domain and alignment annotation for each sequence. HMMER2 commands used were:

hmmbuild <hmm file> <alignment file>

hmmcalibrate <hmm file>

hmmsearch <query or hmm file> <target or sequence file>

Essentially the same procedures were used for SAM and GGSEARCH, and the designations used for Proteins 1 and 2 were the same.

### SAM [42,43]

The homologous sequences from Protein-1 were trained to build the model. The model was then searched against the database consisting of homologous sequences from Protein-2. The homologues of both proteins were generated using NCBI-BLASTP searches with a cutoff e-value of 0.001, and the redundant sequences were removed with CD-HIT before building the model. The reverse was true for values provided in the bottom entries. The SAM commands used were:

buildmodel <model name> - train <training set> -randseed0

hmmscore <output> -i <model file> -db <target sequence file> -sw 2 -calibrate 1

### GGSEARCH

GGSEARCH of the FASTA package from the University of Virginia http://fasta.bioch.virginia.edu/fasta_www2/fasta_www.cgi?rm=select&pgm=gnw was used to compare the homologous FASTA sequences retrieved with Protein-1 to those obtained with Protein-2. The best hit from each comparison was from the resulting output file. Format of presentation was as described above.

### Hydropathy Plots

**Ave**rage **h**ydropathy, **a**mphipathicity and **s**imilarity plots for sets of homologues were generated with the AveHAS program [44] while **w**eb-based **h**ydropathy, **a**mphipathicity, and predicted **t**opology for an individual protein were estimated using the WHAT program [45]. These programs were updated as described in [28,46]. Sequences were spliced for statistical analyses as described by Zhou *et al*., [31].

## Results & Discussion

### CRAC channels

Cai [47] carried out phylogenetic analyses of Orai channel subunits, identifying potential Orai homologues in *Urochordata *and (incorrectly) in Archaea. They correctly reported two conserved apparent internal repeat sequences in TMSs 1 and 3, both of which were known to contain residues key to channel formation [47]. We here extend these results. Three multiple alignments upon which the results reported below were based can be found in Supplementary figures S1A, B and C [Additional files 1, 2 and 3], and the proteins presented are tabulated in Tables S1, S2 and S3 [Additional files 4, 5 and 6] (see our website: http://www.biology.ucsd.edu/~msaier/supmat/Crac. Table S1 presents representative homologues of CRAC channels. Three human and three mouse orthologues were identified, Orai1, Orai2 and Orai3, which can be found in clusters 1, 2 and 3 of the phylogenetic tree, respectively; [Additional file 7: Figure S2A] [see also [47]]. This tree is based on the multiple alignment shown in [Additional file 1: Figure S1A]. The chicken and frog have Orai1 and Orai2 but not Orai3, which seems to be specific to mammals. *Danio rerio *has only Orai1. Additionally, single copies of Orai proteins are found in sea urchins (cluster 4), insects (cluster 5), and roundworms (cluster 6) [Additional file 7: Figure S2A].

**Figure 1:**
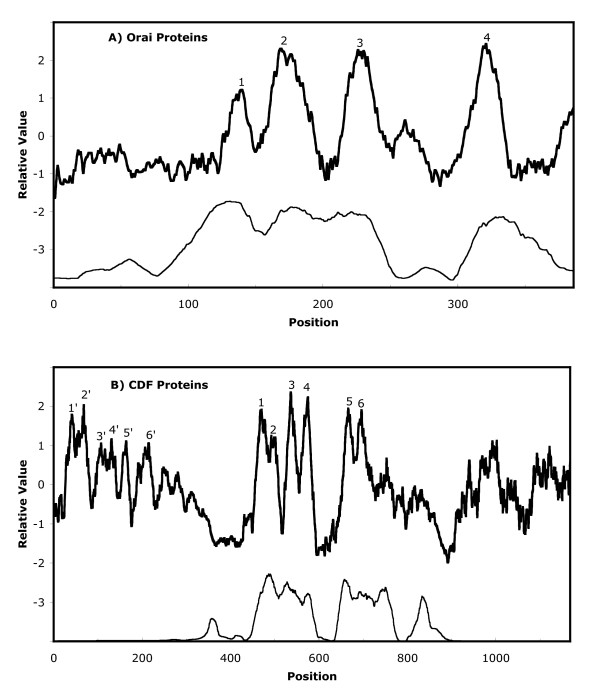
**Average hydropathy and similarity plots for Orai and CDF homologues**. (A) Topological analyses of Orai protein homologues (see Table S1). The AveHAS program [28] was used to depict average hydropathy (top dark line) and average similarity (bottom light line) based on a ClustalX multiple alignment. The four conserved peaks of hydrophobicity, believed to correspond to the four TMSs, are labeled 1-4. (B) Topological analyses of CDF protein homologues (see [Additional file 5: Table S2]). The majority of CDF proteins contain six TMSs and correspond to the six conserved peaks labeled 1-6. Two roundworm members contain twelve TMSs (see text). The N-terminal 6 TMSs of these latter proteins are labeled 1'-6'. Position: alignment position in the multiple alignment.

Average hydropathy and similarity plots for the Orai homologues are shown in Figure 1A. Four peaks of hydrophobicity and similarity coincide. These correspond to predicted TMSs 1-4. Between TMSs 3 and 4 is a region of low sequence similarity, not present in all Orai proteins. The N- and C-termini are predicted to be in the cytoplasm as documented previously [7].

The Stim proteins can be found in [Additional files 2, 8 and 5; Figures S1B, S2B and Table S2].Table S2 presents the corresponding Stim1 homologues. Mammals, as well as the chicken and the frog, possess Stim1 and Stim2 but not Stim3. *Danio rerio *and all other organisms represented have only one Stim homologue. The phylogenetic tree, based on the multiple alignment presented in Figure S1B, is shown in [Additional file 8: Figure S2B]. The phylogenetic patterns suggest that the Orai and Stim proteins evolved in parallel with a couple of potential exceptions. The average hydropathy and similarity plots [Additional file 9: Figure S3] revealed that the single large peak of hydrophobicity, corresponding to the predicted TMS in Stim proteins [48], occurs in a well conserved portion of the alignment.

### CDF antiporters

[Additional file 9: Table S3] presents 122 members of the CDF family. These proteins derive from every major domain and kingdom of living organisms for which sequence data are available in the NCBI database, suggesting that they are essentially ubiquitous. Montanini et al. [48], have analyzed the phylogenetic distribution of CDF homologues and established that these proteins fall into three major and two minor clusters. The major clusters segregate according to substrate specificity (cluster 1, Zn^2+^; cluster 2, Fe^2+^/Zn^2+^, and cluster 3, Mn^2+^). The proteins analyzed here are all in cluster 1 of Montanini et al. [48].

The average hydropathy and similarity plots for the ClustalX aligned CDF sequences (see [Additional file 3: Figure S1C] for the multiple alignment) are shown in Figure 1B, while the phylogenetic tree is shown in [Additional file 10: Figure S2C]. Six well conserved central peaks and six poorly conserved N-terminal peaks of hydrophobicity can be seen. The latter transmembrane domain is homologous to the central domain and represents an internal repeat sequence in just 2 orthologues, those from the roundworms, *C. elegans *and *C. briggsae*. One protein, from the β-proteobacterium, *Polynucleobacter *sp. QLW-PIDMWA-1, has a large hydrophilic C-terminal domain that proved to be homologous to the MhpC predicted hydrolase/acyltransferase of the α/β hydrolase superfamily [49]. In the studies reported below, only the homologous 6 TMS CDF proteins were analyzed.

### Internal repeats in 6 TMS CDF homologues

**Figure 2:**
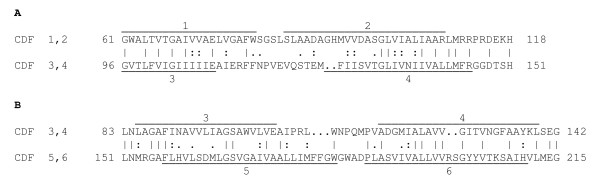
**Identification of three 2TMS homologous repeat sequences in the 6TMS CDF antiporters**. (A) Alignment of CDF TMSs 1-2 of Aod (*Actinomyces odontolyticus*, gi# 154507747) with CDF TMSs 3-4 of Ssa (*Staphylococcus saprophyticus*, gi# 73662044). (B) Alignment of CDF TMSs 3-4 of Ilo (*Idiomarina loihiensis*, gi# 56460742) with CDF TMSs 5-6 of Bsp (*Bacillus sp*. B14905, gi# 126652939). The IC program was used to identify internal segments exhibiting the greatest statistical similarity [29]. The GAP program [32] was used to generate the alignment with default settings and 500 random shuffles). Numbers at the beginning and end of each line indicate residue numbers in the proteins. |, identity; :, close similarity; ., more distant similarity as defined by the GAP program. This convention of presentation is also used in Figures 3 and 4. In all three figures, positions of the TMSs were predicated using the TMHMM program.

We examined 6 TMS CDF proteins for the occurrence of internal repeats. Three such repeats were found, each consisting of a two TMS hairpin structure with the N- and C-termini inside (see Introduction). Binary alignments are depicted in Figure 2A-2B, and the statistical analyses are presented in Table 1A. The results establish that the 6 TMS CDF antiporters consist of three 2 TMS hairpin repeats.

**Table 1:** Internal Repeats in CDF carrier and CRAC channel proteins.

**A**. Comparison of CDF 2TMS segments						
**TMS # of**	**TMS # of**	**No. of Residues**	**Comparison**	**%**	**%**	**No. of**
**CDF proteins**	**CDF proteins**	**Compared**	**score (SD)**	**Identity**	**Similarity**	**Gaps**

1 and 2	3 and 4	58	12.2	28.6	44.6	1
3 and 4	5 and 6	65	11.0	36.7	48.3	2
1 and 2	5 and 6	55	9.0	28.6	35.7	1

**B**. Comparison of CDF 2TMS segments with Orai 2TMS segments						

**TMS # of**	**TMS # of**	**No. of Residues**	**Comparison**	**%**	**%**	**No. of**
**CDF proteins**	**Orai proteins**	**Compared**	**score (SD)**	**Identity**	**Similarity**	**Gaps**

3 and 4	1 and 2	72	14.6	36.4	51.5	2
5 and 6	3 and 4	55	8.6	33.3	48.1	1
3 and 4	3 and 4	80	6.5	29.8	35.1	1
5 and 6	1 and 2	70	2.4	26.5	26.5	0
1 and 2	1 and 2	26	2.3	46.1	53.8	1
1 and 2	3 and 4	33	-0.1	20.0	26.7	0

When TMSs 1-2 (segment 1-2) of CDF proteins were compared with TMSs 3-4 (segment 3-4) of homologous CDF carriers, the highest comparison score was obtained (12.2 S.D.). This value corresponded to 28.6% identity and 44.6% similarity with a single gap. (see Figure 2A and Table 1). When segment 3-4 was compared with segment 5-6, a score of 11 S.D., corresponding to 36.7% identity and 48.3% similarity with two gaps was obtained (see Figure 2B and Table 1A). These values are sufficient to establish homology [33]. Finally, only short regions of segment 1-2 and segment 5-6 gave good scores (up to 9 S.D.). This score of 9 S.D. was based on an alignment with 28.6% identity and 35.7% similarity with one gap (Table 1). Because of the shortness of this sequence, this value is insufficient to establish homology. However the sequences compared and the values obtained in Table 1A are sufficient to establish homology. Thus, based on the Superfamily Principle [50], since TMSs 1-2 are homologous to TMSs 3-4, and TMSs 3-4 are homologous to TMSs 5-6, TMSs 1-2 must be homologous to TMSs 5-6.

### Homology of CDF antiporters with Orai channel proteins

A CRAC channel homologue of *Caenorhabditis elegans*, Orai1a (gi# 211593603; e^-33^; 42% identical, 63% similar to the mouse Orai2 (TC# 1.A.52.1.3; Q8BH10)) was used as the query sequence to screen the NCBI database. Three archaeal sequences that proved to be members of the CDF family of heavy metal:proton antiporters were retrieved below threshold. The best protein pair for establishing homology between these three similar archaeal proteins and established members of the CDF family was a *Pyrococcus furiosus *homologue (gi# 1876930) compared to the *Bacillus subtilis *CzcD protein (TC# 2.A.4.1.3). This pair gave a comparison score of e^-54 ^(39% identity and 61% similarity).

Each of these three archaeal homologues was compared with the conserved region of the *C. elegans *Orai1a homologues using BlastP http://blast.ncbi.nlm.nih.gov/Blast.cgi?PROGRAM=blastp&BLAST_PROGRAMS=blastp&PAGE_TYPE=BlastSearch&SHOW_DEFAULTS=on&BLAST_SPEC=blast2seq&LINK_LOC=blasttab&LAST_PAGE=blastn&BLAST_INIT=blast2seq&LAST_PAGE=blastn&BLAST_INIT=blast2seq. All three scores were similar with values of ~e^-6^. The best score was obtained with the *P. furiosus *sequence which yielded an e-value of 6e^-7 ^with 34% identity and 57% similarity with no gaps for a 48 residue comparison (residues 3-51 in the *C. elegans *Orai1a protein and 62-110 in the *P. furiosus *CDF protein. These regions correspond to TMSs 1-2 in the Orai protein and TMSs 3-4 in the CDF protein.

The *C. elegans *Orai protein sequence and the *P. furiosus *CDF protein sequence were used as query sequences in NCBI BLAST searches. Eleven of each of the retrieved sequences, each from a different species, were multiply aligned giving the multiple alignment shown in [Additional file 11: Figure S4]. As can be seen, there are three identities and many positions (28%) where only conservative substitutions occur. These results also support the conclusion of homology between the CDF carriers and CRAC channels.

**Figure 3:**
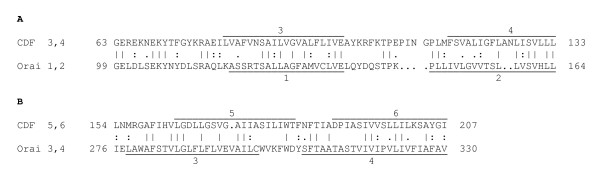
**Comparisons obtained when the 2 TMS hairpin segments from Orai homologues were compared with 2 TMS segments of CDF carriers**. (A) The maximal comparison score value was obtained when segment 3-4 of CDF was compared with segment 1-2 of Orai (14.6 S.D.). (B) When segment 5-6 of a CDF homologue was compared with segment 3-4 of an Orai homologue, the second largest score (8.6 S.D.) was obtained. Significant similarity was not found when TMSs 1-2 of CDF proteins were compared with CRAC Orai homologues (Table 1B), leading to the proposal set forth in Figure 5.

When the two 2 TMS hairpin segments from Orai1 homologues were compared with the three 2 TMS hairpin segments of CDF carriers, comparison scores were obtained as reported in Table 1. The maximal value was obtained with the GAP program when segment 3-4 of CDF was compared with segment 1-2 of Orai (Figure 3A; Table 1B; 14.6 S.D.). When the same segments were compared using the GLSEARCH program and the GGSEARCH program, e-values of e^-20 ^and x e^-19 ^were obtained, respectively. When segment 5-6 of a CDF homologue was compared with segment 3-4 of an Orai homologue (Figure 3B), the second largest score (8.6 S.D.) was obtained. All other values were much lower (see Table 1B). These results provide convincing evidence that segment 1-2 of Orai arose from segment 3-4 of CDF, that segment 3-4 of Orai arose from segment 5-6 of CDF, and that segment 1-2 of CDF was lost during evolution of the CRAC channels from CDF carriers (see also the Conclusions section).

**Figure 4:**
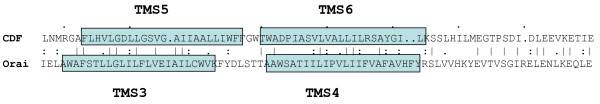
**Alignment of the Consensus Sequence for the CDF Family (TMSs 5 and 6 and flanking regions) with the Consensus Sequence for the Orai Family (TMSs 3 and 4 and flanking regions)**. The consensus sequences were generated using the HMMER package (see Methods). The two consensus sequences were aligned using the GAP program (GCG package). This alignment shows 30% identity and 46% similarity with 3 gaps. TMSs were predicted with the TMHMM program http://www.cbs.dtu.dk/services/TMHMM. Each amino acid shown in the consensus sequence is the highest probability amino acid at that position according to the Hidden Markov Model.

In order to gain confirmatory evidence for homology between CDF carriers and Orai channels, the HMMER package was used to derive consensus sequences for both families, and these were aligned using the GAP program (see the Methods section). The results are presented in Figure 4. Alignment of the two consensus sequences revealed 30% identity and 46% similarity with three gaps. In this alignment, TMSs 5 - 6 of the CDF family consensus sequence aligned with TMSs 3 - 4 of the Orai family as expected. These values qualitatively confirm the quantitative measurements presented above.

### Evaluation of four programs designed to detect and evaluate sequence similarity

In an earlier publication [38], five programs ((1) IC/GAP, (2) LALIGN, (3) GGSEARCH, (4) GLSEARCH and (5) PairwiseStatSig) were compared to evaluate the capabilities of these programs to detect sequences similarities in distantly related proteins. Based on the e-values obtained, GGSEARCH and GLSEARCH proved to be more sensitive than LALIGN and PairwiseStatSig [38]. In this section, we compare both closely and distantly related representative proteins from four different superfamilies as well as their internal repeat sequences, using IC/GAP and GGSEARCH as well as two additional programs, HMMER and SAM. The superfamilies include (1) the CRAC/CDF Superfamily described here, (2) the Drug/Metabolite Transporter (DMT) Superfamily [51], (3) the Bile acid/Arsenite/Riboflavin Transporter (BART) Superfamily [52] and (4) the Oligopeptide Transporter (OPT) Superfamily [53; K.M. Gomolplitinant & M. H. Saier, manuscript in preparation]. The results are presented in Table 2.

**Table 2:** Comparison of IC, GGSEARCH, HMMER and SAM: Results for Homologous Proteins in Four Superfamilies^1^.

Superfamily	Family TC#	Profile		Database		IC/GAP score (S.D.)	GGSEARCH (FASTA) e-value	HMMER e-value	SAM e-value
						
		**Protein-1**^**2**^	Acc#	**Protein-2**^**2**^	Acc#				
**CDF Vs Orai**	2.A.4.1	PfuCDF	AAL80682	CelOrai	NP_497230	14.6	0.0049	0.09	0.72
**Orai Vs CDF**	1.A.52.1	CelOrai	NP_497230	PfuCDF	AAL80682		5.4 e^-5^	0.22	0.29
									
**CDF 3-4 TMSs Vs Orai 1-2 TMSs**	2.A.4.1	PfuCDF	AAL80682	CelOrai	NP_497230	14.6	1.6 e^-18^	0.11	0.18
**Orai 1-2 TMSs Vs CDF 3-4 TMSs**	1.A.52.1	CelOrai	NP_497230	PfuCDF	AAL80682		4.7 e^-6^	0.11	0.02
									
**DMT**	2.A.7.20	PfCRT	Q86M68	AthCRT	Q8RWL5	16	0	9.2 e^-226^	0
**DMT**	2.A.7.20	AthCRT	Q8RWL5	PfCRT	Q86M68		0	2.6 e^-149^	3.26 e^-163^
									
**DMT**	2.A.7.12	SLC35A1	Q8BRW7	PfCRT	Q86M68	9	6.9 e^-10^	3.2 e^-6^	8.55 e^-9^
**DMT**	2.A.7.20	PfCRT	Q86M68	SLC35A1	Q8BRW7		3.8 e^-8^	9.5 e^-6^	2.17 e^-6^
									
**BART**	2.A.87.2 (P-RFT)	YpaA	NP_390186	Ade1	YP_464235	9	0.0002	0.007	5.4
**BART**	2.A.59.1 (Acr3)	Ade1	YP_464235	YpaA	NP_390186		0.02	0.11	0.2
									
**BART**	9.B.33 (SHK)	LytS	NP_847838	Rba2	NP_868846	8	0.04	0.08	0.03
**BART**	2.A.93 (UNK)	Rba2	NP_868846	LytS	NP_847838		0.09	0.007	0.28
									
**BART**	9.B.34 (KPSH)	Dge1	YP_604037	Rba2	NP_868846	9	0.004	2.2	0.3
**BART**	2.A.93 (UNK)	Rba2	NP_868846	Dge1	YP_604037		0.024	0.06	7.2
									
**OPT ABvsCD**	2.A.67.3	Spr1	YP_001477255.1	Lsa1	YP_394932.1	13	3.5 e^-8^	4 e^-4^	0.1
**OPT CDvsAB**	2.A.67.4	Lsa1	YP_394932.1	Spr1	YP_001477255.1		2.3 e^-6^	0.004	0.004
									
**OPT AvsB**	2.A.67.4	Ngo1	YP_208927.1	Sde1	YP_526125.1	11	7.1 e^-6^	0.06	0.5
**OPT BvsA**	2.A.67.4	Sde1	YP_526125.1	Ngo1	YP_208927.1		5.9 e^-5^	0.2	0.09
									
**OPT AvsC**	2.A.67.2	Zma1	NP_001104952.1	Chy1	YP_361078.1	12	5.9 e^-5^	0.03	0.002
**OPT CvsA**	2.A.67.4	Chy1	YP_361078.1	Zma1	NP_001104952.1		0.0015	0.03	0.02
									
**OPT AvsD**	2.A.67.1	Gze4	XP_389463.1	Sus1	YP_822933.1	12	0.0008	0.09	2
**OPT DvsA**	2.A.67.4	Sus1	YP_822933.1	Gze4	XP_389463.1		0.0001	0.03	0.2
									
**OPT BvsC**	2.A.67.1	Sco1	AAF26618.1	Mtu1	NP_216911.1	12	0.007	0.07	0.01
**OPT CvsB**	2.A.67.4	Mtu1	NP_216911.1	Sco1	AAF26618.1		0.003	0.08	0.003
									
**OPT BvsD**	2.A.67.2	Osa28	CAE02279.2	Asu1	YP_001343430.1	14	2.2 e^-8^	0.006	0.02
**OPT DvsB**	2.A.67.4	Asu1	YP_001343430.1	Osa28	CAE02279.2		7 e^-5^	0.007	0.001
									
**OPT CvsD**	2.A.67.4	Pgi1	NP_904744.1	Ani11	XP_658304.1	10	0.0002	0.2	2
**OPT DvsC**	2.A.67.2	Ani11	XP_658304.1	Pgi1	NP_904744.1		9.2 e^-6^	0.05	0.5
									

^1^The comparison scores obtained with the IC and GAP programs, other than the CRAC/CDF comparisons, are published as follows: DMT superfamily: Tran & Saier, 2004; see also Jack et al., 2001; BART superfamily: Mansour et al., 2007. The HMMER and IC/GAP values for comparison of the CRAC (Orai) and CDF families, as well as the OPT family, have not been published previously.

^2^Top entry in each set of comparisons: Protein-2 was used as the Blast query sequence to generate the target sequences; Protein-1 was used as the query sequence to generate the HMM profile. The opposite was used for the bottom entry in each set of comparisons.

The first two entries in Table 2 present comparisons between the CDF family and the CRAC (Orai) family. The first entry compares the complete sequences of both proteins, while the second entry compares TMSs 3-4 in the CDF protein with TMSs 1-2 in the Orai homologue. These are the regions showing the greatest sequence similarity (see Table 1B). These comparisons using the IC/GAP program set gave 14 S.D., a value far in excess of what is required to establish homology. GGSEARCH also gave values sufficient to strongly suggest homology (4.9e^-3 ^and 5.4e^-5^) for the full-length sequences, and 1.6e^-18 ^and 9.4e^-5 ^for the CDF TMSs 3-4 compared with Orai TMSs 1-2. According to the HMMER website, e-values smaller than 0.1 are significant. By this criterion, one value obtained with this program was borderline (0.09). Finally, SAM gave one value (0.02) that was suggestive of homology.

The DMT superfamily was next examined (Table 2). When 2 members of a single family within this superfamily were compared, all four programs predicted homology. The same was true for members of two distinct families within this superfamily (SLC 35A1 with PfCRT), and the degrees of sensitivity detected by the last three programs were GGSEARCH (G) >SAM (S) >HMMER (H).

For the BART superfamily, three different comparisons were run: the first between two families of known transport function, and the second two between families of unknown function where the transmembrane domain may serve as an "anchor" or "receptor" [52]. In the first comparison, the sensitivities of the three programs was G > H > S. In the second and third comparisons, the order was again G > H > S, but S did not give significant e-values.

OPT family members consist of 16TMS proteins that arose by two successive duplication events where a 4TMS encoding genetic segment probably duplicated internally to give an 8TMS product, and the gene encoding this duplicated product again duplicated internally to give the current 16TMS members of the family (K.M. Gomolplitinant & M.H. Saier, unpublished results).

The two 8TMS halves and the four 4TMS repeat units in members of this family were compared with each other using all four programs (Table 2; OPT; bottom). When the two halves were compared, IC/GAP gave 13 S.D., far in excess of what is required to establish homology (9-10 S.D.) The other three programs also detected similarity with scores that were G > H > S. When the 4TMS repeat units were compared, values of 10-14 S.D. were obtained with IC/GAP. Scores for detection of similarity by the other 3 programs were in three cases G > H > S and in three cases G > S > H.

When considering all fourteen comparisons (Table 2), eight showed G > H > S, five showed G > S > H, and one showed H > S > G. Thus, while we consider IC/GAP to be the "gold standard" for establishing homology, we suggest that of the three remaining programs, for the purpose of detecting sequence similarities, GGSEARCH is better than HMMER, which is better than SAM (the most time-consuming program to use). However, since SAM was better than HMMER in five cases, and HMMER was better than GGSEARCH in one case, we conclude that the use of all three of these programs is superior to the use of any one or two of them when time and effort are not limiting. We recommend IC/GAP and GGSEARCH as the two most sensitive programs for detection of significant sequence similarity between distantly related homologues. It should be noted that if one program detects significant sequence similarity, and any number of programs do not, the first program, giving positive results, is to be trusted over those that give negative results because only the first program is likely to have correctly aligned the sequences being compared so as to identify their common features.

## Conclusions

**Figure 5:**
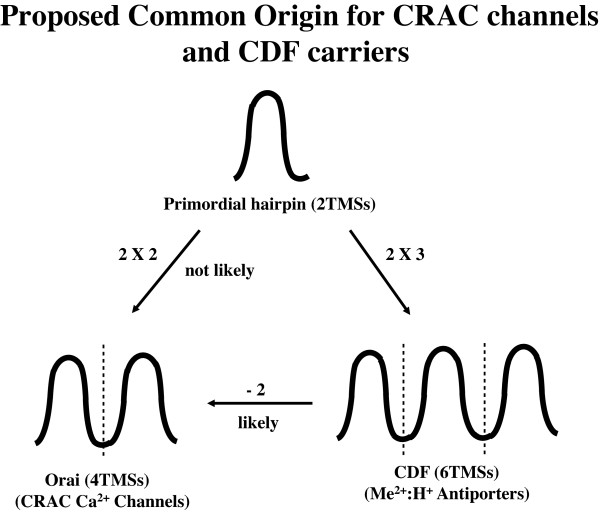
**Proposed Common Origin for CRAC channels and CDF carriers**. The figure illustrates two potential pathways: the likely pathway whereby triplication of the primordial hairpin structure gave rise to a 6 TMS CDF carrier, followed by loss of TMSs 1-2 to give 4 TMS Orai channels. See text for details.

We have shown that the Orai Ca^2+ ^channel proteins of animal CRAC channel complexes are homologous to the ubiquitous metal:H^+ ^antiporters of the CDF family. Our results lead us to suggest that the evolutionary process involved loss of TMSs 1-2 in the primordial CDF carrier, leaving TMSs 3-6 (TMSs 1-4 of Orai). The relative values for the comparison scores when hairpin structures of Orai channels were compared with corresponding hairpin structures of CDF carriers lead to a single preferred prediction for the evolutionary pathway taken, namely that the pathway by which the Orai channel arose from a CDF carrier involved genetic deletion of the first hairpin structure of CDF carriers. The alternative route, direct duplication of the primordial 2 TMS hairpin structures is not favored by the data (Figure 5). Using a total of seven programs for constructing sequence comparisons [38], we conclude that overall, the order of sensitivities and reliabilities for these programs is: IC/GAP = GGSEARCH and GLSEARCH > HMMER, LALIGN and PairwiseStatSig > SAM.

Table 3 compares the properties of CDF carriers (left) with CRAC channels (right). (1) While the former are carriers, the latter are simple channels. (2) While the former are ubiquitous in all domains of life and are found in both plasma and intracellular membranes of eukaryotes, the latter occur specifically at the plasma membrane/endoplasmic reticular junction of animal (and possibly a few other eukaryotic) cells. They presumably arose late in eukaryotes and have not been detected in prokaryotes. (3) Although CDF carriers have 6 TMSs while Orai channels have 4, both consist of 2 TMS repeat units, and both have their N- and C-termini inside; they thus have the same orientation in the membrane. (4) While CDF carriers exhibit tremendous size and sequence variation, suggestive of an ancient origin, CRAC channels show relatively little variation, consistent with a more recent origin. Their restricted organismal distribution compared to the ubiquitous CDF carriers is in agreement with this conclusion. (5) Finally, a pair of acidic residues in both proteins appears to function in cation binding. All of these observations are consistent with the proposed evolutionary pathway.

**Table 3:** Comparisons of CDF Carriers with Crac Channels^1^.

CDF (2.A.4)	Crac-C (1.A.52)
1. Secondary Carriers: catalyze Me^2+^:H^+ ^antiport.	Channels: catalyze bidirectional Ca^2+ ^flux.
2. Ubiquitous; in plasma and intracellular membranes of eukaryotes.	Present only in eukaryotes; at the plasma membrane/endoplasmic reticulum junctions.
3. 6 TMSs; N- and C-termini inside; dimeric.	4 TMSs; N- and C-termini inside; tetrameric.
4. Much size and sequence divergence.	Little size and sequence divergence.
5. Two aspartates are critical for Me^2+ ^binding.	Two glutamates are critical for Ca^2+ ^binding.

^1 ^Abbreviation: Me^2+^, divalent heavy metal ion;

The consequences of our observations are of great importance. For the first time, structural modeling of CRAC channels, based on the known 3-d structure of CDF carriers [24], is possible. Moreover, limited extrapolation of functional and mechanistic data is now feasible. We hope that the bioinformatic analyses reported will greatly accelerate our understanding of the structure-function relationships of CRAC and CDF proteins.

## Competing interests

The authors declare that they have no competing interests.

## Authors' contributions

MGM conducted studies leading to the principle conclusions of this paper under the direction of MHS. KMG and DGT provided extensive confirmation of the results using multiple programs. All authors contributed to manuscript preparation and correction.

## Supplementary Material

Additional file 1 **S1A - Multiple sequence alignment of all Orai proteins included in this study**. The multiple alignment was generated using the CLUSTAL X program (see Methods section). Click here for file

Additional file 2 **S1B - Multiple sequence alignment of all Stim proteins included in this study**. The multiple alignment was generated using the CLUSTAL X program (see Methods section). Click here for file

Additional file 3 **S1C - Multiple sequence alignment of all CDF proteins included in this study**. The multiple alignment was generated using the CLUSTAL X program (see Methods section). Click here for file

Additional file 4 **Table S2 - List of Orai protein sequences from the CRAC-C family included in this study**. Proteins are listed according to cluster number as indicated in Figure S2A. Within each cluster, proteins are presented according to their position in the cluster. Click here for file

Additional file 5 **Table S2 - List of Stim protein sequences from the CRAC-C family included in this study**. Proteins are listed according to cluster number as indicated in Figure S2B. Within each cluster, proteins are presented according to their position in the cluster. Click here for file

Additional file 6 **Table S3 - List of Cation Diffusion Facilitator sequences included in this study**. Proteins are listed according to cluster number as indicated in Figure S2C. Within each cluster, proteins are presented according to their position in the cluster. Click here for file

Additional file 7 **S2A - Phylogenetic tree of Orai proteins.** Protein abbreviations are as indicated in table S1. Clusters are labeled 1-6. The tree was drawn using the TreeView (neighbor joining) program, based on the multiple alignment shown in Figure S1A. Click here for file

Additional file 8 **S2B - Phylogenetic tree of Stim proteins**. Protein abbreviations are as indicated in table S2. Clusters are labeled 1, 2 and 4-6, corresponding to the clusters in figure S2A. The tree was drawn using the TreeView (neighbor joining) program, based on the multiple alignment shown in Figure S1B. Click here for file

Additional file 9 **S3 - Average hydropathy (dark line, top) and average similarity (light line, bottom) plots of Stim protein homologues (see Table S2)**. This plot was generated with the AveHas program (Zhai and Saier, 2001) based on the multiple alignment shown in figure S1B. Click here for file

Additional file 10 **S2C - Phylogenetic tree of CDF proteins.** Protein abbreviations are as indicated in Table S3. Clusters are labeled 1-11. The tree was drawn using the TreeView (neighbor joining) program, based on the multiple alignment shown in Figure S1C. Click here for file

Additional file 11 **S4 - Multiple alignment of eleven Orai homologues with eleven CDF homologues**. The two proteins used as query sequences in BLAST searches were the C. elegans Orai homologue (NP_497230) and the Pyrococcus furiosus CDF homologue (AAL80682). These two proteins plus 10 homologues retrieved from each search with good scores, each from a different species, were included in the ClustalX multiple alignment (see Methods section). With 116 positions shown, there are three positions where only identities (a single residue) are present (asterisks), eighteen positions where only close similarities are present (colons), and eleven positions where only more distant similarities are present (dots) as defined by the ClustalX program (see Methods). Thus, 32 positions, or 28% of all positions, show identities and similarities in all twenty two proteins included in the alignment. The species name, the protein genbank ID#, the starting residue number, the sequence included in the alignment, and the final residue number for each protein are shown in the figure. Click here for file
