# Supplementary material for: Animal Ca2+ release-activated Ca2+ (CRAC) channels appear to be homologous to and derived from the ubiquitous cation diffusion facilitators
Source: BMC Res Notes. 2010 Jun 3;3:158. doi: 10.1186/1756-0500-3-158 (PMC2894845; doi:10.1186/1756-0500-3-158)
Supplement: Additional file 1 — S1A - Multiple sequence alignment of all Orai proteins included in this study. The multiple alignment was generated using the CLUSTAL X program (see Methods section). [file 1756-0500-3-158-S1.PDF]

```
CLUSTAL X (1.82) multiple sequence alignment


Mmu1            ---------------------------MHPEPAPPPSHSNPELPVSGGSSTSGSRRSRRR
Hsa1            ---------------------------MHPEPAPPPSRSSPELPPSGGSTTSGSRRSRRR
Gga1            ------------------------------------------------------------
Xla1            ---------------------------MYPECG----------------VETKSRPCSKQ
Dre1            ------------------------------------------------------------
Mmu3            ---------------------------MK-----------------GGEGDTGEQAPLNP
Hsa3            ---------------------------MK-----------------GGEGDAGEQAPLNP
Mmu2            -------------------------------------------------MSAELNVPMDP
Hsa2            -------------------------------------------------MSAELNVPIDP
Gga2            -------------------------------------------------MSSELNVPVDP
Xla2            -------------------------------------------------MSSELNIPVDP
Spu1            ------------------------------------------------------------
Dme1            ---MSVWTTANNSGLETPTKSPITSSVPRAARSSAVITTGNHQQHHFQHVVAAAVAAATS
Ame1            ------------------------------------------------------------
Aae1            -----------------------MHLHPSCSQSNLCLNSPYYKQFN-KPVSAAAAASKHS
Aga1            ------------------------------------------------------------
Tca1            -------------------------------------------------------MSVWS
Nvi1            MSNNSNNGSSAQLDGAEPSEKAGKRPSAKSLNSKVASVEDLLPSRSSKSTQIDASAWRFQ
Cbr1            -----------------------------MPRSHDPSRVELLRQEGSTMNEKRVSISVED
Cel1            -----------------------------MPRSHDPSRVELLRKEGG-LTEKRVSISVED


Mmu1            SGDGEPSGAPP---------------------------------------LPPPPPAVSY
Hsa1            SGDGEPPGAP-----------------------------------------PPPPSAVTY
Gga1            ------------------------------------------------------------
Xla1            -----------------------------------------------------LQEEVSY
Dre1            ------------------------------------------------------------
Mmu3            EVD-------------------------------------------------SPAGSATY
Hsa3            EGE-------------------------------------------------SPAGSATY
Mmu2            SAPACP---------------------------------------------EPGHKGMDY
Hsa2            SAPACP---------------------------------------------EPGHKGMDY
Gga2            STPACCS--------------------------------------------EPGTKGMDY
Xla2            STPAVS---------------------------------------------ERGNKGMDY
Spu1            ------------------------------------------------------------
Dme1            VATGHQFQQQFPLHAHPHPQHHSNSPTGSGSNSNNSAGFQRTSISNSLLQFPPPPPPSSQ
Ame1            ------------------------------------------------------------
Aae1            SAGG---------------------TGGSAGFAGHNNGIS--SIGATLLGAGSGSGPSHT
Aga1            ------------------------------------------------------------
Tca1            TSGG--------------------------------------------LDPGKTFKAKGD
Nvi1            TRTRPTSMGQLSRHSQFSELSYANYNSSGGGGGGGSPSYSFSTTQSEMVLAPPPSRQRLP
Cbr1            IRGAVATWKTTSGA--PITPYPLPQFFLQPPSSGGGSRNVGGGDGAAGNSKNGSMNSLRM
Cel1            IRGAVANWKNSGGAGDPITPYPLPQFFLQPPSTAGG----GSRNGVG--SKEGSVTSLRM


Mmu1            PDWIGQSY------------------------------------SEVMSLNEHSMQALSW
Hsa1            PDWIGQSY------------------------------------SEVMSLNEHSMQALSW
Gga1            -----------------------------------------------MSLNEHSMQALSW
Xla1            PEWISRSY------------------------------------VELMSLNEHSMQALSW
Dre1            ---------------------------------------------MKMSRSEHSLQALSW
Mmu3            REFVHRGY------------------------------------LDLMGASQHSLRALSW
Hsa3            REFVHRGY------------------------------------LDLMGASQHSLRALSW
Mmu2            RDWVRRSY------------------------------------LELVTSNHHSVQALSW
Hsa2            RDWVRRSY------------------------------------LELVTSNHHSVQALSW
Gga2            RDWVRRSY------------------------------------LELVTSNHHSVQALSW
Xla2            RDWVRRSY------------------------------------LELVTSNHHSVQALSW
Spu1            ------------------------------------------------MSNQHSAQALNW
Dme1            NQAKPRGHHRTA-----------------------------SSSMSQSGEDLHSPTYLSW
Ame1            --------------------------------------------MSQSGDGLHTPGYLSW
Aae1            LGEERRSH---------------------------------ISTMSQSGDEYNSPNYLSW
Aga1            -----RSN---------------------------------LSTMSQTGDDLHTPNYLSW
Tca1            NTLYKRNH---------------------------------QCRMSQSGDDLHSSQYLSW
Nvi1            NNQPSRYTALDMPRVLPSGVSASGGNCCCSCACKAGGPNLGPSTAHTSPENQDGPEGLSW
Cbr1            QAYAKKTD------------------------------------DDVDLGHRGELDLSEK
Cel1            P--LKKAG------------------------------------DDVDLGHRGELDLSEK
                                                                          .

Mmu1            RKLYLSRAKLKASSRTSALLSGFAMVAMVEVQLDTDHDYPP--GLLIVFSACTTVLVAVH
Hsa1            RKLYLSRAKLKASSRTSALLSGFAMVAMVEVQLDADHDYPP--GLLIAFSACTTVLVAVH
Gga1            RKLYLSRAKLKASSRTSALLSGFAMVAMVEVQLDAEHDYPR--GLLIAFSACTTVLVAVH
```

```
Xla1            RKLYLSRAKLKASSRTSALLSGFAMVAMVEVQLEPNHAYPP--GLLIAFSACTTVLVAVH
Dre1            RKLYLSRAKLKASSRTSALLSGFAMVAMVEVQLDTNHDYPP--GLLIAFSACTTVLVAVH
Mmu3            RRLYLSRAKLKASSRTSALLSGFAMVAMVEVQLENDHEYPP--GLLVAFSACTTVLVAVH
Hsa3            RRLYLSRAKLKASSRTSALLSGFAMVAMVEVQLESDHEYPP--GLLVAFSACTTVLVAVH
Mmu2            RKLYLSRAKLKASSRTSALLSGFAMVAMVEVQLETKYQYPQ--PLLIAFSACTTVLVAVH
Hsa2            RKLYLSRAKLKASSRTSALLSGFAMVAMVEVQLETQYQYPR--PLLIAFSACTTVLVAVH
Gga2            RKLYLSRAKLKASSRTSALLSGFAMVAMVEVQLEMQYKYPQ--MLLIAFSACTTVLVAVH
Xla2            RKLYLSRAKLKASSRTSALLSGFAMVAMVEVQLEMKYGYPQ--LLLIAFSACTTVLVAVH
Spu1            RKLYLSRAKLKASSRTSALLAGFAMVAMVEVQLSATKETNAYDPLMIAFSINTTILVVVH
Dme1            RKLQLSRAKLKASSKTSALLSGFAMVAMVEVQLDHDTNVPP--GMLIAFAICTTLLVAVH
Ame1            RKLQLSRAKLKASSKTSALLSGFAMVAMVEVQLNSDTKVPS--EMLIAFAVCTTLLVAVH
Aae1            RKLQLSRAKLKASSKTSALLSGFAMVAMVEVQLDEHTTVPP--SMLIAFAICTTLLVAVH
Aga1            RKLQLSRAKLKASSKTSALLSGFAMVAMVEVQLDENTKVPS--AMLIAFAVCTTLLVAVH
Tca1            RKLHLSRAKLKASSKTSALLSGFAMVAMVELQLQTPMTIPS--TVLVVFAIITTLLVAVH
Nvi1            RRLHMSRAKLKATATTSELLSGFAMVAMVELQINEPTAVPE--WLFVMFAVCTTVLVSVH
Cbr1            YNYDLSKAQLKASSRTSALLAGFAMVCLVELQYDDSTSKPL----LIVLGVVTSLLVSVH
Cel1            YNYDLSRAQLKASSRTSALLAGFAMVCLVELQYDQSTPKPL----LIVLGVVTSLLVSVH
                 .  :*:*:***:: ** **:*****.:**:* .           :: :.  *::** **

Mmu1            LFALMISTCILPNIEAVSNVHNLNSVKESPHERMHRHIELAWAFSTVIGTLLFLAEVVLL
Hsa1            LFALMISTCILPNIEAVSNVHNLNSVKESPHERMHRHIELAWAFSTVIGTLLFLAEVVLL
Gga1            LFALMISTCILPNIEAVSNVHNLNSVKESPHERMHRHIELAWAFSTVIGTLLFLAEVVLL
Xla1            LFALMVSTCILPNIEAVSNVHNLNSVKESPHERMHHHIELAWAFSTVIGTLLFLAEVVLL
Dre1            LFALMVSTCILPNIEAVSNVHNLNSVKESPHERMHRHIELAWAFSTVIGTLLFLAGVVLL
Mmu3            LFALMVSTCLLPHIEAVSNIHNLNSVHQSPHQRLHRYVELAWGFSTALGTFLFLAEVVLV
Hsa3            LFALMVSTCLLPHIEAVSNIHNLNSVHQSPHQRLHRYVELAWGFSTALGTFLFLAEVVLV
Mmu2            LFALLISTCILPNVEAVSNIHNLNSISESPHERMHPYIELAWGFSTVLGILLFLAEVVLL
Hsa2            LFALLISTCILPNVEAVSNIHNLNSISESPHERMHPYIELAWGFSTVLGILLFLAEVVLL
Gga2            LFALLISTCILPNVEAVSNIHNLNSISESPHERMHPYIELAWGFSTVLGILLFLAEVVLL
Xla2            LFALLISTCILPNVEAVSNIHNLNSVNESPHDRMHPYIELAWGFSTALGILLFLAEVVLL
Spu1            MAALLISTCILPNIEAVSNVHNVNAVQESPHNSLAFYIEMSWIFSTVLGIFLFLVEIILL
Dme1            MLALMISTCILPNIETVCNLHSISLVHESPHERLHWYIETAWAFSTLLGLILFLLEIAIL
Ame1            MLALMISTCILPNIEAVCNLHSISLVHESPHERLHWYIEVAWAFSTLLGLLLFLLEIAIL
Aae1            MLALMISTCILPNIETVCNLHSISLVNESPHERLHWYIETAWAFSTLLGLILFLFEIAIL
Aga1            MLALMISTCILPNIETVCNLHSISLVHESPHERLHWYIETAWAFSTLLGLILFLLEIAIL
Tca1            MLALMISTCILPNIEAICNLDAIGLVHESPHERLHWYIETAWAFSTLLGLLLFLAEIAIL
Nvi1            IFALMISTYLLPNVEAISKLQVSRLVTESPHERMRGFIELAWAFSTVLGLFLFLVEVAIL
Cbr1            LLALMMSTCILPYMEATG------CTQDSPHLKLKFYIDLSWLFSTCIGLLLFLVEIGVI
Cel1            LLALMMSTCILPYMEATG------CTQDSPHIKLKFYIDLSWLFSTCIGLLLFLVEIGVI
                : **::** :** :*:           :***  :  .:: :* *** :* :***  : ::

Mmu1            CWVKFLPLKRQAGQPSPTKPPAESV-----IVANHSDSSGIT------------------
Hsa1            CWVKFLPLKKQPGQPRPTSKPPASG-----AAANVS-TSGIT------------------
Gga1            CWVKFLPLKKNPLDPAENS------------------NSSIT------------------
Xla1            CWVKFLPVN----SPKISSNE----------------TSAVS------------------
Dre1            CWVKFLPIK-----PKGQK------------------NGTVS------------------
Mmu3            GWVKFVPIGAPMGKPAPVVPMSQVP----PVTVSLSLASNLTPSSASITTSQQPSKACPP
Hsa3            GWVKFVPIGAPLDTPTPMVPTSRVPGTLAPVATSLSPASNLPRSSASAAPSQAEP-ACPP
Mmu2            CWIKFLPVD---AKDQPGS----HSH----------------------------------
Hsa2            CWIKFLPVD---ARRQPGPPPGPGSH----------------------------------
Gga2            CWIKFLPVDS-ILKNDTTTIQKPSGH----------------------------------
Xla2            CWIKFLPIDSALTRNETATTQKPRGN----------------------------------
Spu1            AWVKFGMAHYN-------------------------------------------------
Dme1            CWVKFYDLS---------------------------------------------------
Ame1            CWVKFYDFS---------------------------------------------------
Aae1            CWVKFYDLN---------------------------------------------------
Aga1            CWVKFYDLN---------------------------------------------------
Tca1            CWVKFYDIN---------------------------------------------------
Nvi1            CWVKFWDYS---------------------------------------------------
Cbr1            FYVKFTAVG---------------------------------------------------
Cel1            FYVKFTAVG---------------------------------------------------
                 ::**

Mmu1            -----------PGEAAAIASTAIMVPCGLVFIVFAVHFYRSLVSHKTDRQFQELNELAEF
Hsa1            -----------PGQAAAIASTTIMVPFGLIFIVFAVHFYRSLVSHKTDRQFQELNELAEF
Gga1            -----------SGQAAAIASTSIMVPFGLIFIVFAVHFYRSLVSHKTDRQFQELNELAEF
Xla1            -----------SGQAAAITSTAIMVPFGLVFIVFAVHFYRSLVSHKTDRQFQELNELAEL
Dre1            -----------AGVAAAITSTSIMVPFGLVFIVFAVHFYRSLVSHKTDRQFQELEELED-
Mmu3            RQVCDS--AHGPGWQAAMASTAIMVPVGLVFMAFALHFYRSLVAHKTDRHKQELEELSR-
Hsa3            RQACGGGGAHGPGWQAAMASTAIMVPVGLVFVAFALHFYRSLVAHKTDRYKQELEELNR-
Mmu2            -----------TGWQAALVSTIIMVPVGLIFVVFTIHFYRSLVRHKTERHNREIEELHK-
Hsa2            -----------TGWQAALVSTIIMVPVGLIFVVFTIHFYRSLVRHKTERHNREIEELHK-
Gga2            -----------AGWQAALVSTIIMVPVGLIFVVFTIHFYRSLVRHKTERHNREIEELHK-
```

```
Xla2            -----------AGWNSALVSTIIMVPVGIIFVIFTIHFYRTLVRHKTERHHQEIEELHK-
Spu1            --------------SAALAATIITAPVLVILIVFAFHFYRRLVSHKTESQTKGLEELES-
Dme1            -------------PPAAWSACVVLIPVMIIFMAFAIHFYRSLVSHKYEVTVSGIRELEM-
Ame1            -------------QVAAWSACIVLIPVLIIFLAFAIHFYRSLVAHKYEVTVSGIRELEL-
Aae1            -------------TTAAWSACIVLIPVLVIFVAFALHFYRSLMTHKYEVTVSGIRELEM-
Aga1            -------------TTAAWSACIVLIPVLVIFVAFALHFYRSLMMHKYEVTVSGIRELEI-
Tca1            -------------KTAAWSASVVLIPILFVFLAFAIHFYRSLVAHKYEMTVSGIRELER-
Nvi1            -------------FTAATASTVIVIPVLIVFIAFAVHFYHSLVVYKCEASVSDMKELEN-
Cbr1            ------------YPTAGYITTAMLIPVGIVFVLFSYLIHKNRVSHSLGRFKDKVDTMKQF
Cel1            ------------YPTAGYITTAMLVPVGVVFVVFSYLIHKNRVSHSLGRFKHKVDTMKQF
                               :.  :  :  *  .::: *:  :::  : :.       :  :

Mmu1            ARLQDQLDHRGDH--SLTPGTHYA--------
Hsa1            ARLQDQLDHRGDH--PLTPGSHYA--------
Gga1            AWLQDQLDHRGDA--ISPAVTHFA--------
Xla1            AQLQDQLDHRGDP--VQSP-VHYA--------
Dre1            --LQNELDHREEVSTLQSPGSLYP--------
Mmu3            --LQGELQAV----------------------
Hsa3            --LQGELQAV----------------------
Mmu2            --LKVQLDGHERSLQVV---------------
Hsa2            --LKVQLDGHERSLQVL---------------
Gga2            --LKVQLDGHDRGMQVV---------------
Xla2            --LKVQLDGHDRGINVV---------------
Spu1            --MNNQLDNGQLQIV-----------------
Dme1            --LKEQMEQDHLEHHNNIRNNGMNYGASGDIV
Ame1            --LKEQIESTDVEGRNGV--NLLQTAGVTHVV
Aae1            --LKEQIEQDHLD-HHHHH--GPPYQS-VQIV
Aga1            --LKEQMEQDHFDQQQHHH--GILHPSGLQIV
Tca1            --LKEQIEQGDIEHNRVNG--VNDLLSGVHIV
Nvi1            --IKRNLDNASLGHSRV---------------
Cbr1            LDVEANLQKSTIAPSTIRDI------------
Cel1            LDVEANLQKSTLAPSTIRDI------------
                  :: :::
```
