# Supplementary material for: Animal Ca2+ release-activated Ca2+ (CRAC) channels appear to be homologous to and derived from the ubiquitous cation diffusion facilitators
Source: BMC Res Notes. 2010 Jun 3;3:158. doi: 10.1186/1756-0500-3-158 (PMC2894845; doi:10.1186/1756-0500-3-158)
Supplement: Additional file 2 — S1B - Multiple sequence alignment of all Stim proteins included in this study. The multiple alignment was generated using the CLUSTAL X program (see Methods section). [file 1756-0500-3-158-S2.PDF]

```
CLUSTAL X (1.82) multiple sequence alignment


Hsa1            ------------------------------------------------------------
Mmu1            ------------------------------------------------------------
Gga1            ------------------------------------------------------------
Xla1            ------------------------------------------------------------
Dre1            ------------------------------------------------------------
Mmu2            ------------------------------------------------------------
Hsa2            ------------------------------------------------------------
Gga2            ---------------------------------------------------MSGLRAPR-
Xtr1            ---------------------------------------------------MNGRKKKRR
Spu1            -------------------------------------------------MASVSVNRKQR
Ame1            -----------------------------------------------------MRSSVIT
Nvi1            ------------------------------------------------------------
Tca1            ----------------------------------------------------------MH
Aae1            ------------------------------------------------------------
Aga1            ------------------------------------------------------------
Dme1            MRKNTIWNYSLIFFCCVLKSISTLDHGPHTVSVDSNRHNTQHQYKQNPNVASQRHSSHES
Cbr1            ------------------------------------------------------------
Cel1            ------------------------------------------------------------


Hsa1            --------------MDVCVRLALWLLWGLLLHQGQSL----SHSHSEKATGTSSGANSEE
Mmu1            --------------MDVCARLALWLLWGLLLHQGQSL----SHSHSEKNTGASSGATSEE
Gga1            ------------------------------------------------------------
Xla1            ---------------MLWCRTLLWALCASFLQPSRPE----SPTES----------TEED
Dre1            --------------MEFSGLVTFWIACICFLLQCRAD----KLNPVN------TDPLPAD
Mmu2            ---------MLLFGLLVAGVADG-CDLVPRHLRGRRA----SGSAGAAASPSAAAAGERQ
Hsa2            ---------MLVLGLLVAGAADG-CELVPRHLRGRRA----TGSAATAASSPAAAAGDSP
Gga2            -GVPAAPGCLLLLGLLLVGAAAAECELEAADVPGGRR----GARGSTAASSSSSSAADSA
Xtr1            LTPPLLPQWVCACGAVERRYPGVCLALLLAVLLLLPP----VSVCTLCEAGGPRRRRDTP
Spu1            LSNRAYRDIYAIPVFIMFVWTTCVCLCVVGQFPVDSTRLTPASGTAKKGGGGTSAPLTDR
Ame1            NVIVLFGLHFLYWCCNTVDASGGALDASSNFQSGSTGSSHSKVTAFSATLTDGLAQAVAH
Nvi1            ------------------------------------------------------------
Tca1            TRGLIFWLILSFYRSAAEDAATEKLKSRDNFKSTYSAN-----------------DLLLS
Aae1            ----------------------------------------------------TYGS----
Aga1            -----------------------------------RNSYTVLSQAMSQAVHHEFGIGAMA
Dme1            GQSLHNSQSEHVTHIAASHAGSGGEHSTHLAQNLHRSSYNLLSEAMSQAVSNEFSSMG-S
Cbr1            ----------------MGNHRTLWNAFAFFCL-------------------------IHI
Cel1            ----------------MG--RVSWIIALYLTIN------------------------VVI


Hsa1            STAAEFCRIDKPLCHSED---EKLSFEAVRNIHKLMDDDANGDVDVEESDEFLREDLNYH
Mmu1            STEAEFCRIDKPLCHSED---EKLSFEAVRNIHKLMDDDANGDVDVEESDEFLREDLNYH
Gga1            -----------------------------------MDDDANGNVDVEESDEFLREDLNYH
Xla1            PMLSEFCRIDKLLCHSTD---ELLSFEAVMSIHKQMDDDANGSVDVEESDEFLREDLNYH
Dre1            NGVSELCRIDEPLCQDEN---AILSFEAIRSIHKQMDDDDNGNVDVLETDGFLREDLNYH
Mmu2            ALLTDPCMSLSPPCFTEE---DRFSLEALQTIHKQMDDDKDGGIEVDESDEFIREDMKYK
Hsa2            ALMTDPCMSLSPPCFTEE---DRFSLEALQTIHKQMDDDKDGGIEVEESDEFIREDMKYK
Gga2            AVVTDPCSSLSPPCFTEE---DRFSLEALRMIHKQMDDDKDGGIEVDESDEFLREDMQYK
Xtr1            ALLSDPFNSLSPPVLTED---DRSSLEAICTIHKQMDDDKDGGIEVEESDEFLREDLNYK
Spu1            SFEYPECSTKDVACIDDHREKDRLGKEGIMLLHRQMDDDHDGDVEPSESDEFLRDELKYE
Ame1            EAGSDTCNDDLACLTTMA-SHDRLGLEAIKSLHSQLDDDANGNVDLSESDDFLREELQYE
Nvi1            ----------------MA-SNDRLGLEAIKSLHSQLDDDANGNIDLSESDDFLREELQYE
Tca1            DSSINTCSIDDFACLTMA-ANDRLGLEAIKTLHQKLDDDANGNVDLSESDDFLREELQYD
Aae1            GSADGACSADDLDCIAH---HDHLGMEAIRSLHQQLDDDDNGDIDLSESDDFLREELKYD
Aga1            GDAGSACTIDDIDCLAH---HDQLGMEAIRSLHQQLDDDDNGDIDLSESDDFLREELKYD
Dme1            GSADGACAADDFDCYSGS-VQDRFGMEAIASLHRQLDDDDNGNIDLSESDDFLREELKYD
Cbr1            VGGAERVTRNVEVTAEEEKIRDKLGYEAIRDIHRDMDDDHSGSIDRNESTGFMKEDMQMR
Cel1            VVNGDRVTRNVEVTAEEEKIRDKLGYEAIRDIHRDMDDDHSGSIDRNESTGFMKEDMQMR
                                                   :*** .*.::  *:  *::::::

Hsa1            DPTVK--HSTFHGEDKLISVEDLWKAWKSSEVYNWTVDEVVQWLITYVELPQYEETFRKL
Mmu1            DPTVK--HSTFHGEDKLISVEDLWKAWKSSEVYNWTVDEVIQWLITYVELPQYEETFRKL
Gga1            DPTVK--HSTFHGEDKLISVEELWKAWKTSEVYNWTVDEVVQWLITYVELPQYEETFRKL
Xla1            DPTAK--HSTFHGEDKLISVEDLWNSWKISEVYNWTVDEVAQWLITYVELPQYEETFRKL
Dre1            DPKGK--HNTFHGDDQFISVEDLWNAWKSSEVYNWTVDEVVKWLIDYVELTQYVEAIKKL
Mmu2            DATNK--HSHLHREDKHITVEDLWKQWKTSEVHNWTLEDTLQWLIEFVELPQYEKNFRDN
Hsa2            DATNK--HSHLHREDKHITIEDLWKRWKTSEVHNWTLEDTLQWLIEFVELPQYEKNFRDN
Gga2            DASNK--HSHLHREDKHITIEDLWRRWKTSEVHNWTQEDTLQWLSEFVELPQYEKNFRES
Xtr1            DASQK--HNHLHREDKHITVEDLWAQWKTSEVHNWTEEETLQWLLEFVELPQYEKNFREN
```

```
Spu1                DDFER--HSQLHRNDKEISAEELWKGWKYSEVYNWTVDKTVKWLEDSVDLPQYAERFQNN
Ame1                AGYERRQRAFHHNDDMHISVRELWEAWLRSEVHNWTIEQTSEWLSSNVDLPQYVPTFIQH
Nvi1                AGYERRQRAFHHNDDMHISVRELWDAWLRSEVHNWTIEQTSEWLSTNVELPQYVPTFIQH
Tca1                SGYERRQKAFHRNDDMHISVKELWEAWLKSEVHNWTVEQTTDWLVSSVDLPQYVPSFISH
Aae1                SGYEKRHRAFHFNDDMHISVKELWEAWLRSEVHNWTVDQTTEWLAQSVQLPQYVRLFRLH
Aga1                SGYEKRHKAFHFNDDMHISVKELWEAWLRSEVHNWTVDQTTEWLAQSVQLPQYVPLFRLH
Dme1                SGYEKRQKAFHFNDDMHISVKELWEAWLRSEVHNWTIEQTTDWLAQSVQLPQYVDLFKLH
Cbr1                GSERTRRENKFHGDDEAITVDDLWEAWFESIERTWTNERLVEWLINDVNIPSIIETVKLK
Cel1                GSERTRRENKFHGDDDAITVDDLWEAWFESIERTWTNERLVEWLINDVNLPSIVEAVKAK
                           .     :*  *:  :**  *  *   .** :   .**   *::..    .

Hsa1                QLSGHAMPRLAVTNTTMTGTVLKMTDRSHRQKLQLKALDTVLFGPPLLTRHNHLKDFMLV
Mmu1                QLTGHAMPRLAVTNTTMTGTVLKMTDRSHRQKLQLKALDTVLFGPPLLTRHNHLKDFMLV
Gga1                QLSGHAMPRLAVNNATMMGTVLKMTDRSHRQKLQLKALDTVLFGPPLLTRHNHLKDFMLV
Xla1                QLSGRDMPRLAIANATMTGTLLKMTDRSQRQKLQLKALDTVLFGPPLLTRHNHLKDFMLV
Dre1                NFSGTAMPRLAVKNNTLTQTVLKILDRSHVQKLQLKALDTVLFGAPMMNRHNHLKDFMLV
Mmu2                NVKGTTLPRIAVHETSFMISQLKISDRSHRQKLQLKALDVVLFGPLTRPPHNWMKDFILT
Hsa2                NVKGTTLPRIAVHEPSFMISQLKISDRSHRQKLQLKALDVVLFGPLTRPPHNWMKDFILT
Gga2                NVNGTTLPRIAVNEHAFMISHLKIIDRSHRQKLQLKALDVVLFGPLTRPPHNWMKDFVLT
Xtr1                SVKGTALPRIAVNEPVLMISQLKITDRSHRQKFQLKALDVVLFGPPTRPPHNWVKDFILT
Spu1                GIEGSALPRLATNNGHFMTAILGITDPIHRQKISLKAMDVVLFGPP-KPMHNYLKDWLLV
Ame1                RVTGATLPRLAVNNMHYLTNVLGIKDPIHKQKIALKAMDVVLFGPPKDTGH-SVKDLILI
Nvi1                RVTGATLPRLAVNNMHYLGNVLGIKDPIHKQKIALKAMDVVLFGPPKDTGH-SLKDLILI
Tca1                KVTGANLPRLAANNVNYLN-HLGIKDPIHKQKIVLKAMDVVLFGPPKDGQP-HWKDLTLI
Aae1                KVTGKVLPRLAVNNMHYVSNVLGIKDPIHKQKIALKAMDAVLFGPPRETGT-RWKDLLLV
Aga1                KVTGKVLPRLAVNNMHYVSNVLGIKDPIHKQKIALKAMDAVLFGPPRETGT-RWKDLLLV
Dme1                KVTGAALPRLAVNNLQYVGNVLGIKDPIHKQKISLKAMDVVLFGPPRETGT-RWKDYILV
Cbr1                KIDGKILPRFASPTSDYLNKELGIKSSVYRQKLRLNSLDVVLFGYKDNNNK--TKDILLA
Cel1                KIDGKILPRFASPNSDFLNKELGIKSSVYRQKLRLNSLDVVLFGYKDNNNR--TKDILLA
                     . *  :**:*          * : .    **: *:::*.****          **  *

Hsa1                VSIVIGVGGCWFAYIQNRYSKEHMKKMMKDLEGLHRAEQSLHDLQERLHKAQEEHRTVEV
Mmu1                VSIVIGVGGCWFAYIQNRYSKEHMKKMMKDLEGLHRAEQSLHDLQERLHKAQEEHRTVEV
Gga1                VSIVIGVGGCWFAYIQNRYSKEHMKKMMKDLEGLHRAEQSLHDLQERLQKAQEEHRSVEV
Xla1                VSIVIGVGGCWFAYIQNRYSEDHMKKMMKDLEGLHRAEQSLHDLQERLQKAQEEHHTVEV
Dre1                VSIVIGMGGCWFAYIQNRHSKDHMKKMMKDLDSLQRAEQSLHDLQQKLQIAQEEHRSVEV
Mmu2                ISIVIGVGGCWFAYTQNKTSKEHVAKMMKDLESLQTAEQSLMDLQERLEKAQEENRTVAV
Hsa2                VSIVIGVGGCWFAYTQNKTSKEHVAKMMKDLESLQTAEQSLMDLQERLEKAQEENRNVAV
Gga2                VSIVIGFGGCWFAYTQNRTSKEHITKMMKDLESLQTAEQSLLDLQERLEKAQEENRNVAV
Xtr1                ISIVIGVGGCWFAYTQNRTSKEHISQMMKDLEGLQKAEISLLELQDRLEKAQEENRTVAV
Spu1                ISLVVALGGCWFAFIQHRFSQSHVKQLLREMESLSSAEDSLKELQEKLNVAQEDHKSAVR
Ame1                TLLFGALIGCWYAYQQKKNSQKHLHRMMKDMESLHKAELALEDLQKELERARMEQESVTT
Nvi1                TLLFGALIGCWYAYQQKKSSQKDLHRMMKDMESLHKAELELKSLQKEFEIVKMEQESATT
Tca1                VLLIGGGIGVWYAFQQNKKFKNHLNRMNRDMDSLQNAEKALENLQKELEEAKQAQEDVIT
Aae1                TLLLTAIIGSWYAYQQNKNAKIHLKRMAKDVEGLLKAEVALKDMQKELERARLEQENVDK
Aga1                TLLLTAIIGSWYAYHQNKSAKIHIRRMAKDVEGLLKAEVALKEMQKELEQARIEQENVGK
Dme1                TLLLSAIIGCWYAYQQNKNAKRHLRRMAQDMEGLQRAEQSLQEMQKELERARMEQENVAT
Cbr1                FLALLLTSLIFLYVRQKQKAQHKLNELSNKLTELKSMETEFEDVQKMLSDERS-------
Cel1                FLALLLTSLIFLYVRQKQKAQQKVNELSNKLTELKCMETEFEDVQKMLNDERS-------
                       .      :    *::  : .: .: ..:  *   *  : .:*. :   :

Hsa1                EKVHLEKKLRDEINLAKQEAQRLKELREGTENE-----RSRQKYAEEELEQVREALRKAE
Mmu1                EKVHLEKKLRDEINLAKQEAQRLKELREGTENE-----RSRQKYAEEELEQVREALRKSE
Gga1                EKVHLEKKLQDEISIAKQEAHRLRELREGTENE-----LSRQKYAEQELEQVRMALKNAE
Xla1                EKVHLEKKLQNEISLAKQEAQRLRELREGTENE-----LSRQKYAEQELEQVRMALKNAE
Dre1                EKVNLEQKLRDEINTAKQEAQRLKELREGTENE-----LSRQKYAEEELEQVRMALKKAE
Mmu2                EKQNLERKMMDEINYAKEEACRLRELREGAECE-----LSRRQYAEQELEQVRMALKKAE
Hsa2                EKQNLERKMMDEINYAKEEACRLRELREGAECE-----LSRRQYAEQELEQVRMALKKAE
Gga2                EKQNLERKMMDEINDAKREAHRLRELREGAECE-----LSRLKYAEEELVQVRMALKKAE
Xtr1                EKQNLERKMMDEISDAKREAHRLRELREGAECE-----LSRLKYAEEELVQVRMALKKAE
Spu1                EKQSIEKQLTDEIETAKHEARRLERERIGDDTDGNSDLATQLSLAEEELVQVRSALDEAE
Ame1                EKQNLEKRLQDESVGLHAS-YSDLEVSQ----------------LKAEIEMLKVELQRAE
Nvi1                EKQNLEKRLKDESMGLNTS-SSDLEVSQ----------------LKAEIEMLKMELQRAE
Tca1                EKQNLEKKLQDSKTDLDSLPYSDLEVNQ----------------LKAEIEMLRTELQIAE
Aae1                EKMDLERRLREAPS-TSSS-NSDLMVQE----------------LKQEIEMLRSELSRAE
Aga1                EKMDLERRLREAPT-LSSS-SSDLELQQ----------------LKQEIEVLRSELNRAE
Dme1                EKLDLERRLKEAPT-LSSS-NSDLEVQQ----------------LKKEIEMLRNELSRAE
Cbr1                -----KRSISDGIVS-------------------------------KTEMENLRVQLEEAE
Cel1                -----KRSISDGVVN-------------------------------HTEMENLRVQLEEAE
                         :: : :                                  . *:  ::  *  :*

Hsa1                KELESHS-SWYAPEALQKWLQLTHEVEVQYYNIKKQNAEKQLLVAKEGAEKIKKKRNTLF
Mmu1                KELESHS-SWYAPEALQKWLQLTHEVEVQYYNIKKQNAERQLLVAKEGAEKIKKKRNTLF
```

```
Gga1            KELESHC-SWAAPEALQKWLQLTHEVEVQYYNIKKQNAEKQLLVAKEGAEKIKKKRNTLF
Xla1            KELESHS-NWSAPDALQKWLQLTHEVEVQYYNIKKQNAEKQLMLAKEGAEKIKKKRNTLF
Dre1            KELESRS-SWSPPEALQKWLQLTHEVEVQYYNIKKQNAERQLQVAKEGAEKIKKKRNTLF
Mmu2            KEFELRS-SWSVPDALQKWLQLTHEVEVQYYNIKRQNAEMQLAIAKDEAEKIKKKRSTVF
Hsa2            KEFELRS-SWSVPDALQKWLQLTHEVEVQYYNIKRQNAEMQLAIAKDEAEKIKKKRSTVF
Gga2            KEFELRS-NWSVPEALQKWLQLTHEVEVQYYNIKRQHAEMQLAIAKDEAEKIKKKRSTVF
Xtr1            KEFELRS-NWSVPETLQKWLQLTHEVEVQYYNIKKQNAEMHLAIAKDEAE----------
Spu1            RRLSEEI-NWSAPMTLQQWLQLTYEIEYRYYVSKRVAAAKQLQVAKEECEKLRRKRASLI
Ame1            GELEDR--CWSPPPGLQHWLQLTHEIENKAYTKKKISAEKQLQQAREACEKLRKKRSSLV
Nvi1            GELEDR--CYSPPVGLQHWLQLTHEIENKSYMKKKQSAEKQLQSAREACEKLRKKRSSLV
Tca1            GELKDR--CWSPPPGLQQWLQLTHEIENKAYMKKKASAEKQLQQAREACEKLRKKRSSLV
Aae1            VEINDH--CWTPPQGLQNWLQLTFELENKQHIRKRVMAEKQLMQAREACEKLKKKRSSLV
Aga1            VEIHDH--CWTPPQGLQSWLQLTYELENKHHIRKRIMAEKQLEQAREACEKLRKKRSSLV
Dme1            FELVDN--CWSPPPQLQSWLQYTYELESKNHQKKRTSAEKQLQSAREACEKLRKKRSSLV
Cbr1            RRLEANTNGSGTPLALQPLLRRTCENEMAFLEKQRQDCFKEMKEAIEMVDRLQKKQGSVL
Cel1            RRLEANSNGSQAPLALQPLLRRTCENEMAFLEKQRQDCFKEMKEAIEMVDRLQKKQGSVL
                 .:  .      *  **  *: * * *      ::  .  .:  * :  :

Hsa1            GTFHVAHSSS--LDDVDHKILTAKQALSEVTAALRERLHRWQQIEILCGFQIVNNPGIHS
Mmu1            GTFHVAHSSS--LDDVDHKILTAKQALSEVTAALRERLHRWQQIEILCGFQIVNNPGIHS
Gga1            GTFHVAHSSS--LDDVDHKILTAKQALSEVTAALRERLHRWQQIELLCGFQIVNNPGIHT
Xla1            GTFHVAHSSS--LDDVDHKILTAKQALSEVTAALRERLHRWQQIETLCGFQIVNNPGLHA
Dre1            GTFHVAHSSS--LDDVDHKILAAKQALGEVTAALRERLHRWQQIELLTGFTLVHNPGLPS
Mmu2            GTLHVAHSSS--LDEVDHKILEAKKALSELTTCLRERLFRWQQIEKICGFQIAHNSGLPS
Hsa2            GTLHVAHSSS--LDEVDHKILEAKKALSELTTCLRERLFRWQQIEKICGFQIAHNSGLPS
Gga2            GTLHVAHSSS--LDEVDHKILEAKKALSELTTCLRERLYRWQQIEKICGFQIAHNSGLPS
Xtr1            ------------------------------------------------------------
Spu1            GSFRIAHGTS--LDAVDQRIVSARTALHEVTAELKERTHRWNQIESVCQFAIMNNPGFQI
Ame1            GAFVSTHGKS--IDEVDKSIVEARTALNEVTAELQERVHRWKQIELLCGFNIINNNGLSY
Nvi1            GAFVSTHGKS--IDEVDKSIVEARTALNEVTAELQERVHRWKAIELLCGFNIINNNGLSY
Tca1            GAFVSTHGKS--IDEVDRSIVEARTALNEVTQELQERVHRWKQIEMLCGFSIINNNGLQF
Aae1            GAFVSTHGKS--IDDVDRSIVEARNALNDVTNDLQERMHRWKNIENLLGFSIVNNNGLAY
Aga1            GAFVSTHGKS--IDDVDRSIVEARNALNDVTNDLQERMHRWKQIETMLGFGIVNNSGIAH
Dme1            GAFVSTHGKS--IDDVDRSIVEARNALGDVTNELQERLHRWKQIETCLGLNIVNNNGLPY
Cbr1            SSLKLATGAASTSDQVDSKIFALKNRMEKIHTLTRETQERWMQIESLCGFPLLYLNETEH
Cel1            SSLKLATGAASTSDQVDSKIFALKSRMEKIHTLTRETQERWLQIESLCGFPLLYLNETEH


Hsa1            LVAALNIDPSWMGSTRP-----NPAHFIMTDDVDDMDEEIVS------------PLSMQS
Mmu1            LVAALNIDPSWMGSTRP-----NPAHFIMTDDVDDMDEEIVS------------PLSMQS
Gga1            LASALNIDPGWMGTPRP-----NPSHFIMTDDVDDLDEEIVS------------PMSMQS
Xla1            LMTALNIDPSLMGVSRP-----APTHFIMSDDLDDLDEDIVS------------PITMQS
Dre1            LASALNLDPSFMGG-RG-----TPQHF-MSDDMDDMDEDIVP------------PGTLQS
Mmu2            LTSSLYSDHSWVVMPRV-----SIPPYPIAGGVDDLDEDTPPIVPQFPGTVAKPAGSLAR
Hsa2            LTSSLYSDHSWVVMPRV-----SIPPYPIAGGVDDLDEDTPPIVSQFPGTMAKPPGSLAR
Gga2            LTSSLYSDHSWVVMPRV-----SIPPYPIAGGVDDLDEDTPPIVSQFHGSIVKPPSTLAR
Xtr1            ------------------------------------------------------------
Spu1            LMASMG----------------GIPNGADLPGMVNLNK----------------PSPLPL
Ame1            LETVLY-RGTPNGRGLG-------LRGRLS-SQDDLDDEASSVYSPSSCGAAG-------
Nvi1            LENILY-RGAPNGRSLG-------FRGRLS-SQDDLDDDVSSLYTPSVSGAAGRLSVKSC
Tca1            LENTLY-RNT-NGRALP-------VRGRIS-SQDDLDDDTASLY-----GHQG-------
Aae1            LENLLYNRNSVGGKSYRCEYYFLSLSTREVVSQDDLDDDS--------------------
Aga1            LENLLYNRNGVAGKTYRCKHPTAAVMSRLSSSQDDLDDDS--------------------
Dme1            LENVLYGRNG-GLQSSMGMSSTKGSRARITNSTEDLDDES--------------------
Cbr1            LNRAVNANSHFYNNSQEG----SSSSGSITNSANQQNLAKKP------------------
Cel1            INRSI-ASSHFYNKSHEG----SSSSGSISNAHSNPNAVN--------------------


Hsa1            PSLQSSVRQRLTEP--------------------------------------QHGLGSQR
Mmu1            PSLQSSVRQRLTEP--------------------------------------QLGLGSQR
Gga1            PALPSTVRQRLVDP--------------------------------------QHGHGSQR
Xla1            PNLS--LRQRHVDS--------------------------------------QLALGPQR
Dre1            PSMMS-LRQRHIDP--------------------------------------QMALGSQR
Mmu2            SSSLCRSRRSIVPSSPQSQRAQLPAHAPLAAHPRHPHHPQHPQHSLPSPDPDILSVSSCP
Hsa2            SSSLCRSRRSIVPSSPQPQRAQLAPHAPHPSHPRHPHHPQHTPHSLPSPDPDILSVSSCP
Gga2            SSSLCRSRRNVVPSSPQSQ------------------------HALHSPDPDILSVSSCP
Xtr1            ------------------------------------------------------------
Spu1            P-----------------------------------------------------------
Ame1            -----------------------------------------------TADNLTWKESSVP
Nvi1            NLCLNLRRRMTAIGTEEDV-------------------------YIGLPESLGWKESSIP
Tca1            ------------------------------------------------------KSISFD
Aae1            ---------------------------------------------------VQGKQLSFD
Aga1            ---------------------------------------------------VQGKPISFD
```

```
Dme1            ---------------------------------------------------IQGK-LNFE
Cbr1            --------------------------------------------------PSSITTTTST
Cel1            ---------------------------------------------------SNFVKKVSP


Hsa1            DLTHSDSESSL-HMSDRQR---------VAPKPPQMSR---AADEALN-AMTSNGRHRLI
Mmu1            DLTHSDSESSL-HMSDRQR---------VAPKPPQMGR---AADEALN-AMPSNGSHRLI
Gga1            DLTRCDSESSIPHLSEAQRLPSAA-PKLLAARPALLTR---SIEDAVP-GHTPNGGSRHA
Xla1            DLSRSDSESSIPYMIEQRVSG----HSSKIPSSKALPR---TLEEVPSGSQTPNGGNRHL
Dre1            DLNRSDSDSSLCISQTGEQLRLSYSSKGFPVKPTSLLHGPHSRSEEGAQSHTHNGGNRVH
Mmu2            ALYRNEEEEEAIYFTAEKQWEVPDTASECDSLNSSSGR---KPSPPSSLEMYQTLSSRKI
Hsa2            ALYRNEEEEEAIYFSAEKQWEVPDTASECDSLNSSIGR---KQSPPLSLEIYQTLSPRKI
Gga2            ALYRTEEEEEAIYFSADKQWEVQETGSECDSLNSSIGR---KQSPPSSLEMYQTLSPQKV
Xtr1            ------------------------------------------------------------
Spu1            ------------------------------------------------------------
Ame1            PDSSSSETG-KETPPESNVVHFTVGDAPDEPIRSSNKEKSGIVRSYSQDTNMLLPVEDKT
Nvi1            PDSSSSETGGKDTPPEA--VHFTLGDGLEESSPTSP------VAVPALAPTAAAPTTTTT
Tca1            DDSHDSD-----------------------------------------------------
Aae1            NFSVFSSE----------------------------------------------------
Aga1            NFSMFSSE----------------------------------------------------
Dme1            NFSLLATE----------------------------------------------------
Cbr1            SAPQATTSATIQFVPTG-------------------------------------------
Cel1            PIPPSQQTANLRFVPTE-------------------------------------------


Hsa1            EGVHPGSLVEKLPDSPALAKKALLALNHGLDKAHSLMELSPSAPPGGSPHLDSSRSHSPS
Mmu1            EGVHPGSLVEKLPDSPALAKKTFMALNHGLDKAHSLMELNPSVPPGGSPLLDSSHSLSPS
Gga1            EPPGP----ERPSESPSVMKK-MMMVNHGMEKSSSLGEIGHPAASKHS-HSDSSRSHSPS
Xla1            ELANVGAGHESVPESP-------QMSNKLSEKSPSLGEISTAG--TIQSHSDSSRSHSPS
Dre1            DGGPS---PDGVPDSP-----ILMKKLYGIEKSASMSEIHGSQ--AAMSMSESSRSLSPN
Mmu2            SRDELSLEDSSRGESP-VTADVSRGSPECVGLTETKSMIFSPASRVYNGILEKSCSMHQL
Hsa2            SRDEVSLEDSSRGDSP-VTVDVSWGSPDCVGLTETKSMIFSPASKVYNGILEKSCSMNQL
Gga2            SREELSLEESSTGDSSSLTADISRGSPDCVGMAETKSMIFSPASKVYNGILEKSCSMNQL
Xtr1            ------------------------------------------------------------
Spu1            ------------------------------------------------------------
Ame1            TSSFLSKTSYSENSLDSSNQDRGGQQRIGPNPCTVPASSVTSINSASGQSCSNNRKGASR
Nvi1            PMPLVPRQDRLLAAFDKIPEDK----VLGS---SSSSTMVRSVSQDTNLILADER-AVPA
Tca1            ------------------------------------------------------------
Aae1            ------------------------------------------------------------
Aga1            ------------------------------------------------------------
Dme1            ------------------------------------------------------------
Cbr1            --------------------------------KTDGSIHSEDVSPVVEDQITIPRSLTQD
Cel1            --------------------------------QSD-SIHSEDTSPIVED-VAISRSLTQD


Hsa1            SPDPDTPSPVGDSRALQASRNTRIPHLA--GKKAVAEEDNGSIGEETDSSPGRKKFPLKI
Mmu1            SPDPDTPSPVGDNRALQGSRNTRIPHLA--GKKAMAEEDNGSIGEETDSSPGRKKFPLKI
Gga1            STDPDTPSPISDCRPN-SSKSTRIPQLA--AKKSAGDEDGSLTGDEVDPGQSKKKFPLKI
Xla1            STEADTPSPGTESKHN-NSKGTRIPQLA--GKKAVAEDDSGSTGEDTDSVSGKKKLTLKI
Dre1            STEPDTPSPTG---LTGGKAGNRIPQIS--SKKSPLEEDSGSTGEDTDSAASRKKHTFKI
Mmu2            SSGIPVPHPRHTSCSSAGNDSKPVQEASNVSRVSSIPHDLCHNGEKSKKPSKIK----SL
Hsa2            SSGIPVPKPRHTSCSSAGNDSKPVQEAPSVARISSIPHDLCHNGEKSKKPSKIK----SL
Gga2            SSGIPVVKPRHTSCSSASSDSKPSQEVCSVPRISSIPQDLYQNGEKNKKPSKIK----SL
Xtr1            ------------------------------------------------------------
Spu1            ------------------------------------------------------------
Ame1            EGQATSAGDDAETLSTDSSSTMDNDDPKKRSRKILFAFKRNKPKEVSSLIFLAGNVYIYT
Nvi1            TTTASNTTSSFLTKTSYSENSLEQAASGSVEKLGRLSTLKKSLRDLPTVMSLDEETLSTD
Tca1            ------------------------------------------------------------
Aae1            ------------------------------------------------------------
Aga1            ------------------------------------------------------------
Dme1            ------------------------------------------------------------
Cbr1            LSE-DMQSIVSGSTNGTS--SGIKKRKGILPKLFRRNTSKSSSLGGTSN-----------
Cel1            LAEADMQSIVSGSTNGSGSVAALKKRKGIFPKLFRRNTSKSSSLGGTSN-----------


Hsa1            FKKPLKK-----------------------
Mmu1            FKKPLKK-----------------------
Gga1            FKEPRK------------------------
Xla1            FKKPKK------------------------
Dre1            FKKQKK------------------------
Mmu2            FKKKSK------------------------
Hsa2            FKKKSK------------------------
Gga2            FKKKCK------------------------
```

```
Xtr1          ------------------------------
Spu1          ------------------------------
Ame1          P--TEDIDILFEYSSRLS-DANSSSFETIS
Nvi1          SNSTADNDDMKRRRRKLHFPAFRKSKNKPS
Tca1          ------------------------------
Aae1          ------------------------------
Aga1          ------------------------------
Dme1          ------------------------------
Cbr1          ------------------------------
Cel1          ------------------------------
```
