# Supplementary material for: Animal Ca2+ release-activated Ca2+ (CRAC) channels appear to be homologous to and derived from the ubiquitous cation diffusion facilitators
Source: BMC Res Notes. 2010 Jun 3;3:158. doi: 10.1186/1756-0500-3-158 (PMC2894845; doi:10.1186/1756-0500-3-158)
Supplement: Additional file 3 — S1C - Multiple sequence alignment of all CDF proteins included in this study. The multiple alignment was generated using the CLUSTAL X program (see Methods section). [file 1756-0500-3-158-S3.PDF]

```
CLUSTAL X (1.82) multiple sequence alignment


Sus1           ------------------------------------------------------------
Aba1           ------------------------------------------------------------
Mca1           ------------------------------------------------------------
Tko1           ------------------------------------------------------------
Pfu1           ------------------------------------------------------------
Fba1           ------------------------------------------------------------
Nsp2           ------------------------------------------------------------
Fno1           ------------------------------------------------------------
Cac1           ------------------------------------------------------------
Lhe1           ------------------------------------------------------------
Lre1           ------------------------------------------------------------
Pmo1           ------------------------------------------------------------
Tme1           ------------------------------------------------------------
Sac1           ------------------------------------------------------------
Dol1           ------------------------------------------------------------
Orf1           ------------------------------------------------------------
Rba1           ------------------------------------------------------------
Oin1           ------------------------------------------------------------
Eli1           ------------------------------------------------------------
Sfr1           ------------------------------------------------------------
Pde1           ------------------------------------------------------------
Pcr1           ------------------------------------------------------------
Bli1           ------------------------------------------------------------
Sau1           ------------------------------------------------------------
Sha1           ------------------------------------------------------------
Ssa1           ------------------------------------------------------------
Bsp2           ------------------------------------------------------------
Ccu1           ------------------------------------------------------------
Ame1           ------------------------------------------------------------
Gka1           ------------------------------------------------------------
Emi1           ------------------------------------------------------------
Aae1           ------------------------------------------------------------
Nsp1           ------------------------------------------------------------
Msu1           ------------------------------------------------------------
Cdi1           ------------------------------------------------------------
Cje1           ------------------------------------------------------------
Tva1           ------------------------------------------------------------
Ath1           ------------------------------------------------------------
Vvi1           ------------------------------------------------------------
Clu1           MEEKYGGDVLAGPSGGGGGLGPVDVPSARLTKYIVLLCFTKFLKAVGLFESYDILKAVHI
Tca2           ------------------------------------------------------------
Cel3           --------MLAHDFVKKDDRNFTPTQSSRSASYFVVLVLTKILRCIGVFFVDILAKSSHI
Hma1           ------------------------------------------------------------
Jsp1           ------------------------------------------------------------
Xor1           ------------------------------------------------------------
Mpo1           ------------------------------------------------------------
Dra1           ------------------------------------------------------------
Asp2           ------------------------------------------------------------
Tfu1           ------------------------------------------------------------
Kra1           ------------------------------------------------------------
Ace1           ------------------------------------------------------------
Aod1           ------------------------------------------------------------
Bad1           ------------------------------------------------------------
Str1           ------------------------------------------------------------
Rer1           ------------------------------------------------------------
Nsp3           ------------------------------------------------------------
Rsp1           ------------------------------------------------------------
Csa1           ------------------------------------------------------------
Oal1           ------------------------------------------------------------
Mlo1           ------------------------------------------------------------
Lag1           ------------------------------------------------------------
Oan1           ------------------------------------------------------------
Pat1           ------------------------------------------------------------
Neu1           ------------------------------------------------------------
Gbe1           ------------------------------------------------------------
Pbe1           ------------------------------------------------------------
Rxy1           ------------------------------------------------------------
Sav1           ------------------------------------------------------------
Asp1           ------------------------------------------------------------
```

```
Mfe1          ------------------------------------------------------------
Sce1          ------------------------------------------------------------
Cbu1          ------------------------------------------------------------
Asp3          ------------------------------------------------------------
Mfl1          ------------------------------------------------------------
Cvi1          ------------------------------------------------------------
Psp1          ------------------------------------------------------------
Tca1          ------------------------------------------------------------
Aae2          ------------------------------------------------------------
Cel2          ------------------------------------------------------------
Tca3          ------------------------------------------------------------
Orf2          ------------------------------------------------------------
Bta1          ------------------------------------------------------------
Xla1          ------------------------------------------------------------
Cel1          ------------------------------------------------------------
Tth1          ------------------------------------------------------------
Ddi1          ------------------------------------------------------------
Ath2          ------------------------------------------------------------
Dsp1          ------------------------------------------------------------
Sau2          ------------------------------------------------------------
Cje2          ------------------------------------------------------------
Cfe1          ------------------------------------------------------------
Abu1          ------------------------------------------------------------
Kst1          ------------------------------------------------------------
Lbo1          ------------------------------------------------------------
Pae1          ------------------------------------------------------------
Aph1          ------------------------------------------------------------
Wen1          ------------------------------------------------------------
Mbo1          ------------------------------------------------------------
Mth1          ------------------------------------------------------------
Gbe2          ------------------------------------------------------------
Chu1          ------------------------------------------------------------
Lin1          ------------------------------------------------------------
Rpa1          ------------------------------------------------------------
Van1          ------------------------------------------------------------
Gox1          ------------------------------------------------------------
Afa1          ------------------------------------------------------------
Bph1          ------------------------------------------------------------
Lpn1          ------------------------------------------------------------
Rgr1          ------------------------------------------------------------
Lme1          ------------------------------------------------------------
Sde1          ------------------------------------------------------------
Ilo1          ------------------------------------------------------------
Asp4          ------------------------------------------------------------
Mma1          ------------------------------------------------------------
Pgi1          ------------------------------------------------------------
Bsp1          ------------------------------------------------------------
Efa1          ------------------------------------------------------------
Lla1          ------------------------------------------------------------
Sgo1          ------------------------------------------------------------
Mba1          ------------------------------------------------------------
Dsh1          ------------------------------------------------------------
Ecu1          ------------------------------------------------------------


Sus1          ------------------------------------------------------------
Aba1          ------------------------------------------------------------
Mca1          ------------------------------------------------------------
Tko1          ------------------------------------------------------------
Pfu1          ------------------------------------------------------------
Fba1          ------------------------------------------------------------
Nsp2          ------------------------------------------------------------
Fno1          ------------------------------------------------------------
Cac1          ------------------------------------------------------------
Lhe1          ------------------------------------------------------------
Lre1          ------------------------------------------------------------
Pmo1          ------------------------------------------------------------
Tme1          ------------------------------------------------------------
Sac1          ------------------------------------------------------------
Dol1          ------------------------------------------------------------
Orf1          ------------------------------------------------------------
Rba1          ------------------------------------------------------------
Oin1          ------------------------------------------------------------
```

```
Eli1            ------------------------------------------------------------
Sfr1            ------------------------------------------------------------
Pde1            ------------------------------------------------------------
Pcr1            ------------------------------------------------------------
Bli1            ------------------------------------------------------------
Sau1            ------------------------------------------------------------
Sha1            ------------------------------------------------------------
Ssa1            ------------------------------------------------------------
Bsp2            ------------------------------------------------------------
Ccu1            ------------------------------------------------------------
Ame1            ------------------------------------------------------------
Gka1            ------------------------------------------------------------
Emi1            ------------------------------------------------------------
Aae1            ------------------------------------------------------------
Nsp1            ------------------------------------------------------------
Msu1            ------------------------------------------------------------
Cdi1            ------------------------------------------------------------
Cje1            ------------------------------------------------------------
Tva1            ------------------------------------------------------------
Ath1            ------------------------------------------------------------
Vvi1            ------------------------------------------------------------
Clu1            VQFIFILKLGTAFFMVLFQKPFSSGKSITKHQWIKIFKHAVAGCIISLLWFFGLTLCGPL
Tca2            ------------------------------------------------------------
Cel3            VNLLWLCKIICTCILFPLQKPLSAGKSLKRGIMPMVLKLSLMNSVIEVFWFYGITLCGPL
Hma1            ------------------------------------------------------------
Jsp1            ------------------------------------------------------------
Xor1            ------------------------------------------------------------
Mpo1            ------------------------------------------------------------
Dra1            ------------------------------------------------------------
Asp2            ------------------------------------------------------------
Tfu1            ------------------------------------------------------------
Kra1            ------------------------------------------------------------
Ace1            ------------------------------------------------------------
Aod1            ------------------------------------------------------------
Bad1            ------------------------------------------------------------
Str1            ------------------------------------------------------------
Rer1            ------------------------------------------------------------
Nsp3            ------------------------------------------------------------
Rsp1            ------------------------------------------------------------
Csa1            ------------------------------------------------------------
Oal1            ------------------------------------------------------------
Mlo1            ------------------------------------------------------------
Lag1            ------------------------------------------------------------
Oan1            ------------------------------------------------------------
Pat1            ------------------------------------------------------------
Neu1            ------------------------------------------------------------
Gbe1            ------------------------------------------------------------
Pbe1            ------------------------------------------------------------
Rxy1            ------------------------------------------------------------
Sav1            ------------------------------------------------------------
Asp1            ------------------------------------------------------------
Mfe1            ------------------------------------------------------------
Sce1            ------------------------------------------------------------
Cbu1            ------------------------------------------------------------
Asp3            ------------------------------------------------------------
Mfl1            ------------------------------------------------------------
Cvi1            ------------------------------------------------------------
Psp1            ------------------------------------------------------------
Tca1            ------------------------------------------------------------
Aae2            ------------------------------------------------------------
Cel2            ------------------------------------------------------------
Tca3            ------------------------------------------------------------
Orf2            ------------------------------------------------------------
Bta1            -------------MTVAEATSGSPGTRGWEREVEIQPVTPDFNRSPEREELVLLERRGWA
Xla1            ------------------------------------------------------------
Cel1            ------------------------------------------------------------
Tth1            ------------------------------------------------------------
Ddi1            ------------------------------------------------------------
Ath2            ------------------------------------------------------------
Dsp1            ------------------------------------------------------------
Sau2            ------------------------------------------------------------
Cje2            ------------------------------------------------------------
Cfe1            ------------------------------------------------------------
```

```
Abu1          ------------------------------------------------------------
Kst1          ------------------------------------------------------------
Lbo1          ------------------------------------------------------------
Pae1          ------------------------------------------------------------
Aph1          ------------------------------------------------------------
Wen1          ------------------------------------------------------------
Mbo1          ------------------------------------------------------------
Mth1          ------------------------------------------------------------
Gbe2          ------------------------------------------------------------
Chu1          ------------------------------------------------------------
Lin1          ------------------------------------------------------------
Rpa1          ------------------------------------------------------------
Van1          ------------------------------------------------------------
Gox1          ------------------------------------------------------------
Afa1          ------------------------------------------------------------
Bph1          ------------------------------------------------------------
Lpn1          ------------------------------------------------------------
Rgr1          ------------------------------------------------------------
Lme1          ------------------------------------------------------------
Sde1          ------------------------------------------------------------
Ilo1          ------------------------------------------------------------
Asp4          ------------------------------------------------------------
Mma1          ------------------------------------------------------------
Pgi1          ------------------------------------------------------------
Bsp1          ------------------------------------------------------------
Efa1          ------------------------------------------------------------
Lla1          ------------------------------------------------------------
Sgo1          ------------------------------------------------------------
Mba1          ------------------------------------------------------------
Dsh1          ------------------------------------------------------------
Ecu1          ------------------------------------------------------------


Sus1          ------------------------------------------------------------
Aba1          ------------------------------------------------------------
Mca1          ------------------------------------------------------------
Tko1          ------------------------------------------------------------
Pfu1          ------------------------------------------------------------
Fba1          ------------------------------------------------------------
Nsp2          ------------------------------------------------------------
Fno1          ------------------------------------------------------------
Cac1          ------------------------------------------------------------
Lhe1          ------------------------------------------------------------
Lre1          ------------------------------------------------------------
Pmo1          ------------------------------------------------------------
Tme1          ------------------------------------------------------------
Sac1          ------------------------------------------------------------
Dol1          ------------------------------------------------------------
Orf1          ------------------------------------------------------------
Rba1          ------------------------------------------------------------
Oin1          ------------------------------------------------------------
Eli1          ------------------------------------------------------------
Sfr1          ------------------------------------------------------------
Pde1          ------------------------------------------------------------
Pcr1          ------------------------------------------------------------
Bli1          ------------------------------------------------------------
Sau1          ------------------------------------------------------------
Sha1          ------------------------------------------------------------
Ssa1          ------------------------------------------------------------
Bsp2          ------------------------------------------------------------
Ccu1          ------------------------------------------------------------
Ame1          ------------------------------------------------------------
Gka1          ------------------------------------------------------------
Emi1          ------------------------------------------------------------
Aae1          ------------------------------------------------------------
Nsp1          ------------------------------------------------------------
Msu1          ------------------------------------------------------------
Cdi1          ------------------------------------------------------------
Cje1          ------------------------------------------------------------
Tva1          ------------------------------------------------------------
Ath1          ------------------------------------------------------------
Vvi1          ---------------------------------------------------------MSW
Clu1          RTLLLFEHSDIVVISLLTVLFTSSGGGPAKTRGAAFFIIAVICLLLFDNDDLMAKMAEHP
```

```
Tca2            ------------------------------------------------------------
Cel3            RSVLVFELSSTVLLSAVVSFFKGGNMSTPSKTRGFFLLIVGFVALFLMDRDGTIEDPHHT
Hma1            ------------------------------------------------------------
Jsp1            ------------------------------------------------------------
Xor1            ------------------------------------------------------------
Mpo1            ------------------------------------------------------------
Dra1            ------------------------------------------------------------
Asp2            ------------------------------------------------------------
Tfu1            ------------------------------------------------------------
Kra1            ------------------------------------------------------------
Ace1            ------------------------------------------------------------
Aod1            ------------------------------------------------------------
Bad1            ------------------------------------------------------------
Str1            ------------------------------------------------------------
Rer1            ------------------------------------------------------------
Nsp3            ------------------------------------------------------------
Rsp1            ------------------------------------------------------------
Csa1            ------------------------------------------------------------
Oal1            ------------------------------------------------------------
Mlo1            ------------------------------------------------------------
Lag1            ------------------------------------------------------------
Oan1            ------------------------------------------------------------
Pat1            ------------------------------------------------------------
Neu1            ------------------------------------------------------------
Gbe1            ------------------------------------------------------------
Pbe1            ------------------------------------------------------------
Rxy1            ------------------------------------------------------------
Sav1            ------------------------------------------------------------
Asp1            ------------------------------------------------------------
Mfe1            ------------------------------------------------------------
Sce1            ------------------------------------------------------------
Cbu1            ------------------------------------------------------------
Asp3            ------------------------------------------------------------
Mfl1            ------------------------------------------------------------
Cvi1            ------------------------------------------------------------
Psp1            ------------------------------------------------------------
Tca1            ------------------------------------------------------------
Aae2            ------------------------------------------------------------
Cel2            ------------------------------------------------------------
Tca3            ------------------------------------------------------------
Orf2            ------------------------------------------------------------
Bta1            TQSRFQQAGVRRTSRPEAASLARGASGRRGGRKSKQQQGWAEAVSLSDAGQVGSSGSGDR
Xla1            ------------------------------------------------------------
Cel1            ----------------------------------------------------MTISMISP
Tth1            ------------------------------------------------------------
Ddi1            ------------------------------------------MENFKNNELESSPIINKN
Ath2            ---------------------------------------------MVTPKLHLDLSLTKK
Dsp1            ------------------------------------------------------------
Sau2            ------------------------------------------------------------
Cje2            ------------------------------------------------------------
Cfe1            ------------------------------------------------------------
Abu1            ------------------------------------------------------------
Kst1            ------------------------------------------------------------
Lbo1            ------------------------------------------------------------
Pae1            ------------------------------------------------------------
Aph1            ------------------------------------------------------------
Wen1            ------------------------------------------------------------
Mbo1            ------------------------------------------------------------
Mth1            ------------------------------------------------------------
Gbe2            ------------------------------------------------------------
Chu1            ------------------------------------------------------------
Lin1            ------------------------------------------------------------
Rpa1            ------------------------------------------------------------
Van1            ------------------------------------------------------------
Gox1            ------------------------------------------------------------
Afa1            ------------------------------------------------------------
Bph1            ------------------------------------------------------------
Lpn1            ------------------------------------------------------------
Rgr1            ------------------------------------------------------------
Lme1            ------------------------------------------------------------
Sde1            ------------------------------------------------------------
Ilo1            ------------------------------------------------------------
Asp4            ------------------------------------------------------------
```

```
Mma1            ------------------------------------------------------------
Pgi1            ------------------------------------------------------------
Bsp1            ------------------------------------------------------------
Efa1            ------------------------------------------------------------
Lla1            ------------------------------------------------------------
Sgo1            ------------------------------------------------------------
Mba1            ------------------------------------------------------------
Dsh1            ------------------------------------------------------------
Ecu1            ------------------------------------------------------------


Sus1            ------------------------------------------------------------
Aba1            ------------------------------------------------------------
Mca1            ------------------------------------------------------------
Tko1            ------------------------------------------------------------
Pfu1            ------------------------------------------------------------
Fba1            ------------------------------------------------------------
Nsp2            ------------------------------------------------------------
Fno1            ------------------------------------------------------------
Cac1            ------------------------------------------------------------
Lhe1            ------------------------------------------------------------
Lre1            ------------------------------------------------------------
Pmo1            ------------------------------------------------------------
Tme1            ------------------------------------------------------------
Sac1            ------------------------------------------------------------
Dol1            ------------------------------------------------------------
Orf1            ------------------------------------------------------------
Rba1            ------------------------------------------------------------
Oin1            ------------------------------------------------------------
Eli1            ------------------------------------------------------------
Sfr1            ------------------------------------------------------------
Pde1            ------------------------------------------------------------
Pcr1            ------------------------------------------------------------
Bli1            ------------------------------------------------------------
Sau1            ------------------------------------------------------------
Sha1            ------------------------------------------------------------
Ssa1            ------------------------------------------------------------
Bsp2            ------------------------------------------------------------
Ccu1            ------------------------------------------------------------
Ame1            ------------------------------------------------------------
Gka1            ------------------------------------------------------------
Emi1            ------------------------------------------------------------
Aae1            ------------------------------------------------------------
Nsp1            ------------------------------------------------------------
Msu1            ------------------------------------------------------------
Cdi1            ------------------------------------------------------------
Cje1            ------------------------------------------------------------
Tva1            ------------------------------------------------------------
Ath1            ------------------------------------------------------------
Vvi1            YRIECFPLSSMIVDKWGFS-LFPRENCVRVLPMLLPFLSGFLGCYERVSMNWG-------
Clu1            EGHHDSALTHMLYTAIAFLGVADHKGGVLLLVLALCCKVGFHTASRKLSIDVGGAKRLQA
Tca2            ------------------------------------------------------------
Cel3            THAHHSGLNHLFYHLIGLFGMADHKGGLCILLISLLLRTGYETSFRHLAVEVGGAKRLHT
Hma1            ------------------------------------------------------------
Jsp1            ------------------------------------------------------------
Xor1            ------------------------------------------------------------
Mpo1            ------------------------------------------------------------
Dra1            ------------------------------------------------------------
Asp2            ------------------------------------------------------------
Tfu1            ------------------------------------------------------------
Kra1            ------------------------------------------------------------
Ace1            ------------------------------------------------------------
Aod1            ------------------------------------------------------------
Bad1            ------------------------------------------------------------
Str1            ------------------------------------------------------------
Rer1            ------------------------------------------------------------
Nsp3            ------------------------------------------------------------
Rsp1            ------------------------------------------------------------
Csa1            ------------------------------------------------------------
Oal1            ------------------------------------------------------------
Mlo1            ------------------------------------------------------------
Lag1            ------------------------------------------------------------
Oan1            ------------------------------------------------------------
```

```
Pat1            ------------------------------------------------------------
Neu1            ------------------------------------------------------------
Gbe1            ------------------------------------------------------------
Pbe1            ------------------------------------------------------------
Rxy1            ------------------------------------------------------------
Sav1            ------------------------------------------------------------
Asp1            ------------------------------------------------------------
Mfe1            ------------------------------------------------------------
Sce1            ------------------------------------------------------------
Cbu1            ------------------------------------------------------------
Asp3            ------------------------------------------------------------
Mfl1            ------------------------------------------------------------
Cvi1            ------------------------------------------------------------
Psp1            ------------------------------------------------------------
Tca1            ----MHSDSSGT---------------------------------PKKVIFCVHGKPSAG
Aae2            ----------------------------------------------MKYITSYYEQFIS-
Cel2            ----MYDVFNTF---------------------------------TFHFCSCSLWGCHNK
Tca3            --METLCEHVDA---------------------------------DDENFLLDDENAIKS
Orf2            ---MEPSPAAGG---------LETTR--------LVSP-------RDRGGAGGSLRLKS-
Bta1            VLGSAPGPFAASYTLPLPCGLLRTTRRFTRGSRDLIKPAGAGRSCRRRGGSSGSDSWKTP
Xla1            ----MKGPEKAY----------------------LVSD---------KATKMYSLTMDS-
Cel1            SSIRLSSDKRDS----------------------SSSNLPANIEEDTQSVSSSDSGVSAD
Tth1            ------------------------------------------------------------
Ddi1            NSSHSINNEDNNYYSHNLEHNDNNNNDNNNTITNSHINNHDHKHNHEHEHKHKHNHDHNH
Ath2            VSYLFVSRQTLYRLSS-------------SCLASLFLSFDAFLIARSCFFFQMKDHIHEH
Dsp1            ------------------------------------------------------------
Sau2            ------------------------------------------------------------
Cje2            ------------------------------------------------------------
Cfe1            ------------------------------------------------------------
Abu1            ------------------------------------------------------------
Kst1            ------------------------------------------------------------
Lbo1            ------------------------------------------------------------
Pae1            ------------------------------------------------------------
Aph1            ------------------------------------------------------------
Wen1            ------------------------------------------------------------
Mbo1            ------------------------------------------------------------
Mth1            ------------------------------------------------------------
Gbe2            ------------------------------------------------------------
Chu1            ------------------------------------------------------------
Lin1            ------------------------------------------------------------
Rpa1            ------------------------------------------------------------
Van1            ------------------------------------------------------------
Gox1            ------------------------------------------------------------
Afa1            ------------------------------------------------------------
Bph1            ------------------------------------------------------------
Lpn1            ------------------------------------------------------------
Rgr1            ------------------------------------------------------------
Lme1            ------------------------------------------------------------
Sde1            ------------------------------------------------------------
Ilo1            ------------------------------------------------------------
Asp4            ------------------------------------------------------------
Mma1            ------------------------------------------------------------
Pgi1            ------------------------------------------------------------
Bsp1            ------------------------------------------------------------
Efa1            ------------------------------------------------------------
Lla1            ------------------------------------------------------------
Sgo1            ------------------------------------------------------------
Mba1            ------------------------------------------------------------
Dsh1            ------------------------------------------------------------
Ecu1            ------------------------------------------------------------


Sus1            ------------------------------------------------------------
Aba1            ------------------------------------------------------------
Mca1            ------------------------------------------------------------
Tko1            ------------------------------------------------------------
Pfu1            ------------------------------------------------------------
Fba1            ------------------------------------------------------------
Nsp2            ------------------------------------------------------------
Fno1            ------------------------------------------------------------
Cac1            ------------------------------------------------------------
Lhe1            ------------------------------------------------------------
Lre1            ------------------------------------------------------------
```

```
Pmo1            ------------------------------------------------------------
Tme1            ------------------------------------------------------------
Sac1            ------------------------------------------------------------
Dol1            ------------------------------------------------------------
Orf1            ------------------------------------------------------------
Rba1            ------------------------------------------------------------
Oin1            ------------------------------------------------------------
Eli1            ------------------------------------------------------------
Sfr1            ------------------------------------------------------------
Pde1            ------------------------------------------------------------
Pcr1            ------------------------------------------------------------
Bli1            ------------------------------------------------------------
Sau1            ------------------------------------------------------------
Sha1            ------------------------------------------------------------
Ssa1            ------------------------------------------------------------
Bsp2            ------------------------------------------------------------
Ccu1            ------------------------------------------------------------
Ame1            ------------------------------------------------------------
Gka1            ------------------------------------------------------------
Emi1            ------------------------------------------------------------
Aae1            ------------------------------------------------------------
Nsp1            ------------------------------------------------------------
Msu1            ------------------------------------------------------------
Cdi1            ------------------------------------------------------------
Cje1            ------------------------------------------------------------
Tva1            ------------------------------------------------------------
Ath1            ------------------------------------------------------------
Vvi1            ------------TIRQLGRKRVR--LISLFFTTVILFVPAVVSILMFEAEGDEFSIASLG
Clu1            LSHLISVLLLCPWVIVLCGTTES--KVESWFSLIMPFTTVIFFVMILDFYVDSICSVKME
Tca2            ------------------------------------------------------------
Cel3            LVTAGSAVCLTPVAVLSWALSSSSGASIGYFHYAALLVIIAVFVFVLDFYAESICFQHVA
Hma1            ------------------------------------------------------------
Jsp1            ------------------------------------------------------------
Xor1            ------------------------------------------------------------
Mpo1            ------------------------------------------------------------
Dra1            ------------------------------------------------------------
Asp2            ------------------------------------------------------------
Tfu1            ------------------------------------------------------------
Kra1            ------------------------------------------------------------
Ace1            ------------------------------------------------------------
Aod1            ------------------------------------------------------------
Bad1            ------------------------------------------------------------
Str1            ------------------------------------------------------------
Rer1            ------------------------------------------------------------
Nsp3            ------------------------------------------------------------
Rsp1            ------------------------------------------------------------
Csa1            ------------------------------------------------------------
Oal1            ------------------------------------------------------------
Mlo1            ------------------------------------------------------------
Lag1            ------------------------------------------------------------
Oan1            ------------------------------------------------------------
Pat1            ------------------------------------------------------------
Neu1            ------------------------------------------------------------
Gbe1            ------------------------------------------------------------
Pbe1            ------------------------------------------------------------
Rxy1            ------------------------------------------------------------
Sav1            ------------------------------------------------------------
Asp1            ------------------------------------------------------------
Mfe1            ------------------------------------------------------------
Sce1            ------------------------------------------------------------
Cbu1            ------------------------------------------------------------
Asp3            -----------------------------------------------------MFQLTIR
Mfl1            ------------------------------------------------------------
Cvi1            ------------------------------------------------------------
Psp1            ------------------------------------------------------------
Tca1            ------------------------------------------------------------
Aae2            ------------------------------------------------------------
Cel2            ------------------------------------------------------------
Tca3            ------------------------------------------------------------
Orf2            ------------------------------------------------------------
Bta1            RRPRRGLGGSGLAAGRAAGAARTALVAGERSSLESPAPDSREHVAGRAGPHESGREDASV
Xla1            ------------------------------------------------------------
Cel1            S-----------------------------------------------------------
```

```
Tth1            ------------------------------------------------------------
Ddi1            DHDHNHEEEYGHGN----------------------------------------------
Ath2            DHMVQICGEVSSG-----------------------------------------------
Dsp1            ------------------------------------------------------------
Sau2            ------------------------------------------------------------
Cje2            ------------------------------------------------------------
Cfe1            ------------------------------------------------------------
Abu1            ------------------------------------------------------------
Kst1            ------------------------------------------------------------
Lbo1            ------------------------------------------------------------
Pae1            ------------------------------------------------------------
Aph1            ------------------------------------------------------------
Wen1            ------------------------------------------------------------
Mbo1            ------------------------------------------------------------
Mth1            ------------------------------------------------------------
Gbe2            ------------------------------------------------------------
Chu1            ------------------------------------------------------------
Lin1            ------------------------------------------------------------
Rpa1            ------------------------------------------------------------
Van1            ------------------------------------------------------------
Gox1            ------------------------------------------------------------
Afa1            ------------------------------------------------------------
Bph1            ------------------------------------------------------------
Lpn1            ------------------------------------------------------------
Rgr1            ------------------------------------------------------------
Lme1            ------------------------------------------------------------
Sde1            ------------------------------------------------------------
Ilo1            ------------------------------------------------------------
Asp4            ------------------------------------------------------------
Mma1            ------------------------------------------------------------
Pgi1            ------------------------------------------------------------
Bsp1            ------------------------------------------------------------
Efa1            ------------------------------------------------------------
Lla1            ------------------------------------------------------------
Sgo1            ------------------------------------------------------------
Mba1            ------------------------------------------------------------
Dsh1            ------------------------------------------------------------
Ecu1            ------------------------------------------------------------


Sus1            ------------------------------------------------------------
Aba1            ------------------------------------------------------------
Mca1            ------------------------------------------------------------
Tko1            ------------------------------------------------------------
Pfu1            ------------------------------------------------------------
Fba1            ------------------------------------------------------------
Nsp2            ------------------------------------------------------------
Fno1            ------------------------------------------------------------
Cac1            ------------------------------------------------------------
Lhe1            ------------------------------------------------------------
Lre1            ------------------------------------------------------------
Pmo1            ------------------------------------------------------------
Tme1            ------------------------------------------------------------
Sac1            ------------------------------------------------------------
Dol1            ------------------------------------------------------------
Orf1            ------------------------------------------------------------
Rba1            ------------------------------------------------------------
Oin1            ------------------------------------------------------------
Eli1            ------------------------------------------------------------
Sfr1            ------------------------------------------------------------
Pde1            ------------------------------------------------------------
Pcr1            --------------------------------------MNPDHPNSKKNDHAHQSIPADT
Bli1            --------------------------------------------------------MAHS
Sau1            ------------------------------------------------------------
Sha1            ------------------------------------------------------------
Ssa1            ------------------------------------------------------------
Bsp2            ------------------------------------------------------------
Ccu1            -------------------------------------MSEQDHDERTLHECGDHILGEHA
Ame1            -------------------------------------MQLVPVFFRTNKQSDNWCTVKAD
Gka1            ------------------------------------------------------------
Emi1            ----------------------------------------------MTKKEEHLHVCACS
Aae1            ------------------------------------------------------------
Nsp1            ------------------------------------------------------------
```

```
Msu1          ------------------------------------------------------------
Cdi1          ------------------------------------------------------------
Cje1          -------------------------------MNDPASAAQQQPQDRGGADAHRHGSARSG
Tva1          --------------------------------MIVDNLYHMKVMNKNEAEPGHHHTHSEH
Ath1          ------------------------------------------------------------
Vvi1          WPLAN----------TVLFGVLLTENYSDEKLVSSRDFQREFLVTFFCTLVLELFYFPEL
Clu1          VSKCARYGSFPIFISALLFGNFWTHPITDQLRAMNKAAHQESTEHVLSGGVVVSAIFFIL
Tca2          ---------------------------------MLPLSHKDNLHRGFG------------
Cel3          DPVMAAARWSSVTMFSCAFG-----------LAYLWYGSQNLGDHALTGGVSITVVCFIL
Hma1          ------------------------------------------------------------
Jsp1          ------------------------------------------------------------
Xor1          ------------------------------------------------------------
Mpo1          ------------------------------------------------------------
Dra1          ------------------------------------------------------------
Asp2          ------------------------------------------------------------
Tfu1          ------------------------------------------------------------
Kra1          ------------------------------------------------------------
Ace1          ------------------------------------------------------------
Aod1          ------------------------------------------------------------
Bad1          ------------------------------------------------------------
Str1          ------------------------------------------------------------
Rer1          ------------------------------------------------------------
Nsp3          ------------------------------------------------------------
Rsp1          ------------------------------------------------------------
Csa1          ------------------------------------------MGHSSSGHDHAP------
Oal1          ---------------------------------------------MSDNHAHPS------
Mlo1          --------------------------------MARPGQFRRRRPAEATARGLSRRIRRLS
Lag1          --------------------------------MSDSG---HHDHAENAPHGHS-------
Oan1          ---------------------------------------------------MSP------
Pat1          ------------------------------------------------------------
Neu1          ---------------------------------------------MTVPQPHP-------
Gbe1          -----------------------------------------MTEAIITPASHSH------
Pbe1          ------------------------------------------------------------
Rxy1          ------------------------------------------------------------
Sav1          -------------------------------MQPLPISTAMRLTAVEAPDARGKAHDMSD
Asp1          --------------------------------------MSRGHSHGRGAHGRKHEAPHGE
Mfe1          ------------------------------------------------------------
Sce1          --------------------------------------MGVRDDHGSASDEHQHGR-GAR
Cbu1          ------------------------------------------------------------
Asp3          ILPSPVLASDRSNWRAATPVRRDNRGIPAPPPPCLRQPMSAKHAPHHHGTAYTRAAGPAR
Mfl1          ------------------------------------------------------------
Cvi1          --------------------------------------MTPSFHLHDE------------
Psp1          --------------------------------------MSGSHHFHSHS-----------
Tca1          ----------------------------CCKVLQSEVIDIKEGDSVDTPTKE-NQDATEM
Aae2          ---------------------------------QNYCICINETKQLFQTKLQ-LVALPHL
Cel2          ----------------------------TSTAQNPQCTRVPHIYNTDEGRKT-TVTYDNF
Tca3          ----------------------------CNKCKSPSNGFVTMDDHVDTLEDSPLLINDCY
Orf2          ------------------------------LFTEPSEPLPEES-----KPVEMP---FHH
Bta1          GRQACSRWLVLATARSGTPAPTPTRGTYTTLWQDGTSSIPPSSPGLALQPTELAPQSSHY
Xla1          -------------------------------SEKNNCGKPPLQ------DDENPHIKYHC
Cel1          ------------------------IDHHHHGHGHGHSHGGHGHSHTHNNDDSSSDCSGAG
Tth1          ------------------------------------MDKHNRLGSRYVELVDIENLHKAL
Ddi1          -------------------------ELEHNNDQEHNVGNKNLLTNNNNQSKKKKHGHSHG
Ath2          --------------------------------ETSLVG------------IKKTCGEAPC
Dsp1          ------------------------------------------------------------
Sau2          ------------------------------------------------------------
Cje2          ------------------------------------------------------------
Cfe1          ------------------------------------------------------------
Abu1          ------------------------------------------------------------
Kst1          ------------------------------------------------------------
Lbo1          ------------------------------------------------------------
Pae1          ------------------------------------------------------------
Aph1          ------------------------------------------------------------
Wen1          ------------------------------------------------------------
Mbo1          ------------------------------------------------------------
Mth1          ------------------------------------------------------------
Gbe2          ------------------------------------------------------------
Chu1          ------------------------------------------------------------
Lin1          ------------------------------------------------------------
Rpa1          ---------------------------------------------MSLSKGIELLQARTK
Van1          ------------------------------------------------------------
Gox1          ------------------------------------------------------------
Afa1          ------------------------------------------------------------
```

```
Bph1      -------------------------------------------------MKYTAHQTDSA
Lpn1      ------------------------------------------------------------
Rgr1      ------------------------------------------------------------
Lme1      ------------------------------------------------------------
Sde1      -----------------------------------------------------------M
Ilo1      ------------------------------------------------------------
Asp4      ------------------------------------------------------------
Mma1      ------------------------------------------------------------
Pgi1      ------------------------------------------------------------
Bsp1      ------------------------------------------------------------
Efa1      ------------------------------------------------------------
Lla1      ------------------------------------------------------------
Sgo1      ------------------------------------------------------------
Mba1      ------------------------------------------------------------
Dsh1      ------------------------------------------------------------
Ecu1      ------------------------------------------------------------


Sus1      -----------------------------------MHSHNHSHGHVHG------------
Aba1      ----------------------------------MPHAHSGDLNDAK-------------
Mca1      -------------------------------MPHDDHEHHHAADSAGA------------
Tko1      ------------------------------------MAHHHHG-----------------
Pfu1      ------------------------------------MEESGE------------------
Fba1      ------------------------------------MGHSHSHNHS--------------
Nsp2      ------------------------------------MAHHHHH-----------------
Fno1      -----------------------------------MSNHFYSHHEHSIPHSHHE------
Cac1      -----------------------------------MEKHSHEHHHH--------------
Lhe1      ------------------------------------MNKDHTT-----------------
Lre1      ------------------------------------MDEKVTG-----------------
Pmo1      ------------------------------------MDLNHEEKER--------------
Tme1      ------------------------------------MHS-HEHHHD--------------
Sac1      ------------------MKIDGSFHKIPGPKKRSVMTGHHPQHIS--------------
Dol1      ------------------MGHN----------------HSHSHETT--------------
Orf1      ------------------------------------MPHDHHHQHGHAHGHG--------
Rba1      -----------------------------------MGSCDQNHSENSADNHENCG-----
Oin1      ------------------------------------MAHDHAHID---------------
Eli1      ------------------------------------MAHSHDQAG---------------
Sfr1      ------------------------------------MSNNNHTHS---------------
Pde1      ------------------------------------MGHSHAHEG---------------
Pcr1      HGHVEEG-----------------EESGGKSNTAYRNQDSHDHDEHLEQTVATRD-----
Bli1      HGHT------------------------GHSHHGSRSAN---------------------
Sau1      ---------------------------------MSHSHHHHDH-MHSHVTTNN-------
Sha1      ---------------------------------MTNHSHTHSH-NHSHVHTNN-------
Ssa1      ---------------------------------MS-EHHEHNH-AHGHVHTNN-------
Bsp2      ------------------------------------MGHNHDH-GHDHTHGAN-------
Ccu1      QGECDCNKRIPTHEKLCCNENDNEKHVQKGQDCNEHDHEVHNHDAHAHSHAAN-------
Ame1      FAVSVCCPIANKDRK------RNTMHEHNHTHEHEHDEAHHHHHHHLGAHSAD-------
Gka1      ------------------------MHHHHHHHSHDHHGHHHHHGHHHDHSREGN------
Emi1      HG--------------------------HKQGHHAHALHEHGHRHHDHGAGAS-------
Aae1      ---------------------------------MEREKS---------------------
Nsp1      ------------------------------MFSLQWCECDCTDDPEIILQSQQK------
Msu1      ---------------------MNAQAKKDKQAHHEHHAHSQVHTEHEHSQVPKN------
Cdi1      -------------------------MSNHHHDHHNHHDHHHG-----------AP-----
Cje1      ASAAAVAQARPADARADAPAPAGASEGGHAHSHDSGHAHSHGHSHGHAHSHSHAP-----
Tva1      NESDTTVGLLEPENCEQFLQVAEKNNHFHVHDHDHHHDHDHDHN---------HA-----
Ath1      ------------------------------------MESPESFSTMFMKPIRHIL-----
Vvi1      SLWGLLLCG------LLLYISVRELDPVYSNSLELGMDSSESFTDSIVKPIRHVL-----
Clu1      SANILSSPSKRGQK-GTLIGYSPEGTPLYNFMGDAFQHSSQSIPRFIKESLKQIL-----
Tca2      ----------------------------------------SRIKEKLNGWMRLIF-----
Cel3      ASISLTHSNQPKHRGGHFVGISNTGLPLFTYGEAFLQRTSKSLMLFMKETLNEIL-----
Hma1      ------------------------------------MTLHEHTDEETAEHNLPSK-----
Jsp1      ----------------------------------MSAGHNHAL-----------------
Xor1      -------------MRASHET-----------TTSMGHDHNHAP-----------------
Mpo1      -------------MGHGH---------------AHGGGHGHSHGGGIPSG----------
Dra1      ------------MTGHNHPHEHDPHHHDRADTQEHGHAHGHAHGHDHAAHN---------
Asp2      ----------------------------------MGHDHSHAHGISA-------------
Tfu1      ----------------------------------MGHHHHH-------------------
Kra1      ----------MRHEHSDDPGHGPSGGSPGSPGDHGDHGDHGGRGDHGGSGGHGGHGGHGG
Ace1      -----------------------------------MHGEHG-RTPHP-------------
Aod1      ------MADNRPTPSPGAGSARESAPASTHIDEAVAHSHDHAYSPSGEHGGHS-------
Bad1      ----------------------------------MAHNHAGVDATTAASG----------
Str1      ----------------------------------MGATDGHQPGAVA-------------
Rer1      ------------------------MVDDREGQIEMGHGHGHGVAEQA-------------
```

```
Nsp3      ----------------------------------MGHGHGHSHSAAA-------------
Rsp1      ------------------------------MGVGHGHSHGHTQGSGE-------------
Csa1      ----HDHDHDHGHGHDH-----------------HHE-----HAPTVT------------
Oal1      ----HDHAHDHDHGDDH-----------------HHHGHGHSHAHPVD------------
Mlo1      PAGAHDRSCHLAHTHSH-----------------DHSGHDHEGAGHVHG-----------
Lag1      ----HGHSHDHGHGHGN-----------------DH-GHGHDHTPEVT------------
Oan1      -----EMSHSHSHGHSH-----------------DDHSHDHSHAPTVT------------
Pat1      ------MAHNHSH---------------------------------TE------------
Neu1      ------HPEHGKNGHDD-----------------RDRNHDHNHKQVIT------------
Gbe1      ------THHDHGHAGHD-----------------H-GALGHSHSAGVK------------
Pbe1      ------MHHGHQCAGHS-----------------QEGGFGGHHHHGSD------------
Rxy1      ---------------MT-----------------HANGHAEGHGREADR-----------
Sav1      HGHDHGPHGHTGHSGHS-----------------GHAGHSHGVSADADR-----------
Asp1      RCDGHGH-GHRHPEHEH-----------------DHGEHVHGMGGHHAG-----------
Mfe1      -----------MENHAH-----------------DHHGHSH---GHHG------------
Sce1      RGEGHDHRHHHAPGHDH-----------------DHG-HDHGDPQRHDHSHDLR------
Cbu1      -------------------------------------MHTHEHHHHKHH-----------
Asp3      CGHDHDHGHDHDHSHDHGHEHHPAPGHDHGSAPAAAHGHEHAHGHHHHH-----------
Mfl1      -----------------------------------MSAHHHHDHSHAHG-----------
Cvi1      NGVDHAHDDDHDANHPH------------------LHRHGGPAGGEGRR-----------
Psp1      KPIKPHSQSGVDP---------------------SLHAHKKGDARHSHTKQ---------
Tca1      INK-HCHRG-------------------------KSPEIDRKARRKL-------------
Aae2      IVT-HCHRP-------------------------RMDGVDKIARKKL-------------
Cel2      HCHDEADST-------------------------DSHDSNRRATRIL-------------
Tca3      HDEFHCHNI-------------------------LQQSEDKKAWKKL-------------
Orf2      CHRDPLPPP-------------------------GLTPERLHARRQL-------------
Bta1      CHSQKGPGS-------------------------HGDPKK-RAWRQL-------------
Xla1      HNNNTKAYD-------------------------ARQREQTSAKKKL-------------
Cel1      GGAHKHSHD-------------------------EKYQKGRRAEKVL-------------
Tth1      KDEAVYKTP-------------------------DLQSNKVKALRTL-------------
Ddi1      GGEDGSSSGGGGGRHGHGHSHGGGSGSDHNHGSSDEDDEESKPLNQLRNLDSKKKAR---
Ath2      GFSDAKTS-------------------------SIEAQERAASMRKL-------------
Dsp1      ----------------------------------MSLISSKKTAPDN-------------
Sau2      --------MVNYGSININLRSYKELFLKATILNTTHLFRSGKRMNGKKAN----------
Cje2      --------------------MSSKKGYKMYKFLSHEPLAN-KSCHHNHEE----------
Cfe1      --------------------MQC-----VYGHITHKPLE--QSCHDHASD----------
Abu1      --------------------MAN----CGFGLNEHKPFLNKESEHHHHHDNKHKLSNKNL
Kst1      ------------------------------------------------------------
Lbo1      -------------------------------------MDDFFQLHHVERS----------
Pae1      -------------------MAKRQAIPSRVSINAVKHMEHNEQVLSERTF----------
Aph1      ------------------------------MWCVMTSCDS-QYSPSMHRR----------
Wen1      ----------MLDVQAVAGSNPVLPTFLFMKRFYMTAVHNGKHSAAQSKR----------
Mbo1      -----------------------------MDCPDHTHDHSQDHSHTHNSRK---------
Mth1      -----------------------------------MIAHEVRVIER--------------
Gbe2      -----------------------------------MHAHADTHLDQSITG----------
Chu1      --------------------------------MEHNHTHHHDHSIILTH-----------
Lin1      ----------------------------MGHHHSHNHSHDHSHNPHSEN-----------
Rpa1      RAPRHSPGPLLRAILSTTAFAPHRNEVVAPHPSPMTQIHDHDHHASASEQGHNHGHAHGP
Van1      ------------------------------------MAHSHEHHVSN-------------
Gox1      ----MTPDPHHDCACDHDHHG---TTLPHEHHAHDHADHDHDDHHHDHDHCDGHSHGFGF
Afa1      ---------------------------------MLQMAHTHDHSHEPN------------
Bph1      SVTHQPKAHVHTDACDHAGHDDADHAHSHGDGHTHDQAHDHADASGGKDAHAHHGHGHGH
Lpn1      -----------------------------MSRHSHDGAHHHEHGEVT-------------
Rgr1      -----------------------------MS-------HNHQHHILR-------------
Lme1      ------------------------------------MKKPMEQLKMK-------------
Sde1      AHQDLPQSHSHPKPHSQEAEPAQADKGITDHSQRNTGSHSHNAKHSKKHKHSHSHQHGDF
Ilo1      -----------------------------------MG-HHHNHDHSK-------------
Asp4      ------------------------------------------------------------
Mma1      ------------MDKTIGENTAHGHQAN-DHTHVHEPHHTHDHHSCQHDHASGHSH----
Pgi1      ----------------METKQSHAHACCSEHKHEHEAHHHGHHHHHHHHGESG-------
Bsp1      ----------------------------MGGREMA------DKQSS--------------
Efa1      -----------------------MEVFKISKSEKQ------ENHSHQN------------
Lla1      ----------------------------MSLKNKKALKLEHEHDHLDK------------
Sgo1      ----------------------------MNSKT---------------------------
Mba1      -----------------------MNYLWMIDKFMGDR-----------------------
Dsh1      -----------------------------------MASHSHAGHMPSS------------
Ecu1      ---------------------------------MHGRKHPAQCSHVTCSN----------

Sus1      -----------------------------------------TGRILGWSLLATTVFVLIE
Aba1      -----------------------------------------TSRVLRISLGVTVLYIVLL
Mca1      -----------------------------------------QGRLV-WALILTGGFVVFE
Tko1      ---------------------------------------ELKGR-MLFSFALNIVITLAE
```

```
Pfu1            ---------------------------------------RLKRR-MIISFALNMGIALVE
Fba1            -----------------------------------HSHNDLNGRNLLISIVLNIAITAAQ
Nsp2            ----------------------------------------VKGKNLFITIILNIIITLSQ
Fno1            -------------------------NDDHHAHEHFNDIENLSGKKLFYVVLLNFGITLAE
Cac1            -----------------------------------HDISSISKNKLFFVIIFNFIIALAE
Lhe1            -------------------------------------------KRYVFVTLLNVIITIAE
Lre1            -------------------------------------------KRFLAVTLLNVLITIVE
Pmo1            ----------------------------------------ALDFRLLFSVILNFGITITE
Tme1            ----------------------------------------VVERKLIFSVIFNFLITASE
Sac1            -----------------------------------------WGNRLLMTMMLNLVIPVVQ
Dol1            -----------------------------------------MGSRLLMAMAINLLIPAAQ
Orf1            -------------------------GHHHHHDT-----ESMGDGRLAWAVAVNVVLTVVQ
Rba1            -------------------------GLHHHGLTSAGQFDSVSDSRLLWSVLLNQALTVAQ
Oin1            ---------------------------PESG-----------DRRVSLAIWANALLTVAQ
Eli1            ---------------------------HSHGEE------NLSDRQLILAVAINVLLTLAQ
Sfr1            ---------------------------HAHN----------HDGKLSMAVFINILLTVAQ
Pde1            --------------------------EHGHSHLPG----GVGDGTILIAVVVNLALTGAQ
Pcr1            --------------------------------------TKGYQRTLLISFLIITGYMFVE
Bli1            ------------------------------------------QKALFISFGLIFTFMMIE
Sau1            ------------------------------------------KEVLFISFLIIGLYMFIE
Sha1            ------------------------------------------KKILAISFIIIGLFMVIE
Ssa1            ------------------------------------------KKILLISFLIIGSFMIVE
Bsp2            ------------------------------------------KKVLIVSFIIITSYMVVE
Ccu1            ------------------------------------------KTTLRNSFLIIFGFMLVE
Ame1            ------------------------------------------KKVLSISLLIIGAYMVVE
Gka1            ------------------------------------------KKGLTIALLITAGIMLLE
Emi1            ------------------------------------------KKALLISFILIIIFMVVE
Aae1            ------------------------------------------LKVLAFSFLLIFLFAFIE
Nsp1            ------------------------------------------TKILWLAAGLLTCFFVVE
Msu1            ------------------------------------------KMILGISLAIISCYMVVE
Cdi1            --------------------------------------SSS--RALLLVAGLTFAFFIVE
Cje1            --------------------------------------TDAPLRALLIVLGVTGTVFFAE
Tva1            --------------------------------------AEGVTWRLILMISLTGIFFFAE
Ath1            --------------------------------------SEKKSRKIALFLLINTAYMVVE
Vvi1            --------------------------------------SERKSRKIALFLLINTGYMVVE
Clu1            --------------------------------------EENDSRQIFYFLCLNLLFTFVE
Tca2            --------------------------------------SDRNSRNLFLFLLLNLSFAFVE
Cel3            --------------------------------------MNNDSRRIFWFLCVNLGFCGVE
Hma1            --------------------------------------SSGSTRRLALVAVVNFFGFVIE
Jsp1            -------------------------------------PKGQNQKYLLIAFVLTSTFLVAE
Xor1            -------------------------------------SEIRHETPLWWALGLTATFLLAE
Mpo1            ------------------------------------SAAARNKGRLAWALGLTLTYMFAE
Dra1            ------------------------------------HAAGAGERQLTGALVLTGAFLVLE
Asp2            ------------------------------------TGTGKHRKRLIAVLGITLAVVLIQ
Tfu1            ------------------------------------TAAGAHRGRLLLVLALTLSVMAFQ
Kra1            HGG------------------------------HDHGALTSNRRRLGIALGVTVTILLVE
Ace1            ------------------------------------ADQVIQRRHLRIALALISAFLVAE
Aod1            ---------------------------------HDHSHAGASTKRLGWALTVTGAIVVAE
Bad1            ---------------------------------------AEHQKRLMMSLGLTSTVFIAE
Str1            -----------------------------------DTG-QRHRWRLWTAFTLLGTLMVAE
Rer1            -----------------------------------ASASGKHVSKLWIAVGLGLVTFVTQ
Nsp3            -----------------------------------GHAGGRYRWRLAVTFAMVATFLVVE
Rsp1            -----------------------------------VGPSRGRIRRMVIALVILLAFLVLE
Csa1            ---------------------------------------ADSAKRVKLAMYLTAGFMLAE
Oal1            ---------------------------------------GRNETRTLIAAALTGGFMLAE
Mlo1            ---------------------------------------ATDKKRVLIAACLTAGFMVAE
Lag1            ---------------------------------------DRNARAVALAGLLIAAFMFAE
Oan1            ---------------------------------------SDNERKVLLSFFLILTFMIVE
Pat1            ---------------------------------------SGNSKRLLAAFIITATFMVAE
Neu1            ---------------------------------------AGNERRIRFVFFFTAGYALIQ
Gbe1            -----------------------------------------DERRLALAGMILTVFLGAE
Pbe1            -----------------------------------------DMMRLRIALAIIVLFAAVE
Rxy1            -------------------------------------------RSLAAALAITASYTVAE
Sav1            -------------------------------------------RWLGIALALITLFMAAE
Asp1            ----------------------------------------ASLRRLAASLAITGGVMIAE
Mfe1            -------------------------------------------HKLDLALGLTLLFAIVE
Sce1            ---------------------------------------RAPLRRLIAAFALTTSFLVVE
Cbu1            ----------------------------------------TSHRVLGWAILLTLGFALVE
Asp3            ----------------------------------------TASRWLPLALALTLGFAAVE
Mfl1            ----------------------------------------GGQPSLLLPLLLIAGFAVVE
Cvi1            ----------------------------------------LSSRALAKVLALTCGFALVE
Psp1            ---------------------------------------VSNQNLILIALVLTLGFSGVE
Tca1            ----------------------------------------------IVASVLCVLFMIGE
```

```
Aae2            ----------------------------------------------ILASVLCVIFMIAE
Cel2            ----------------------------------------------WLTVVLCLFFMVCE
Tca3            ----------------------------------------------LIAAILCFFFMITE
Orf2            ----------------------------------------------YAACAVCFVFMAGE
Bta1            ----------------------------------------------CVASAFCLLFMIGE
Xla1            ----------------------------------------------CIASLICFVFISAE
Cel1            ----------------------------------------------WAVAALSAVFIAAE
Tth1            ----------------------------------------------KIVCCISTSFMCIE
Ddi1            -------------------------------------------YSLILALTLTTIFMVGE
Ath2            ----------------------------------------------LIAVLLCAIFIVVE
Dsp1            --------------------------------------QMPVSNKLKLGILFSGIILLAE
Sau2            ----------------------TINRYKYFHHVNHQKIQQSSKKTLWASLIITLLFTVIE
Cje2            ------------------HSHEH--HHSH------ADVRSVDKKILKISLLMTFSMMLVQ
Cfe1            ------------------HHRAH--SHSH------SSAN-TNKKVLKISLFITVAAMIFQ
Abu1            EQNIEQKFVFSAKQENHIHSHEHGCGHDHGVAGHTHDHRGTDKRVLKWALGITMITMFLE
Kst1            -----------------------------------------------MASAITGVIFIVE
Lbo1            -------------------------------------QEKGLKRSILLAILVSFSIFLVE
Pae1            -------------------------------------KGRKAKKGLRFAVSVTGLIFLVE
Aph1            ---------------------------------------------MVMAIVVVACTLVVE
Wen1            ---------------------------------------------LIYSIIIVAITMFME
Mbo1            -------------------------------------------KPLKLAIALTALIFVAE
Mth1            --------------------------------------------RFSLAMILTISILIAE
Gbe2            --------------------------------------------RFRYAIILTAITLVAE
Chu1            -----------------------------------------VNTAFIVGIILNLLFVVIE
Lin1            -----------------------------------------AGKSLVIAMIFNLVYAGIE
Rpa1            -GGHVHAP-------------------------------KDFGRAFALGIGLNIAFVITE
Van1            -----------------------------------------YNSAFAIGIVLNLLYVIVE
Gox1            GHQHVHAP-------------------------------ASFGMAFAVGITLNTAYVAGE
Afa1            ----------------------------------------SYGFAFGFGILLNLAFVAIE
Bph1            GHHHHHHVP-----------------------------AAGHGRAFAIAVALNVAIVVVQ
Lpn1            -----------------------------------------YDKAFIIAITANGLFVVMQ
Rgr1            -----------------------------------------LNVSFALAVLLNFGFVFTE
Lme1            ------------------------------------------QMPYLIGISLNMIFVLSE
Sde1            --------------------------------------TDMSSNRIGWAFVLNVSFTIIE
Ilo1            ---------------------------------------DMPSNRLGWAFLLNFVFTIIE
Asp4            ---------------------------------------------------MNLSFTILE
Mma1            --------------------------------------VHPVTSNLKVAFFLNFCFTIIE
Pgi1            -------------------------------------------SNLLFAFAMNLFFAIVE
Bsp1            ------------------------------------------VKNTKVAFFINLLFAIVE
Efa1            ----------------------------------------QPNKNIKIAFFTNFIFSMIE
Lla1            ----------------------------------------LVSQNVTIVFALNLFFAILE
Sgo1            --------------------------------------------SVWIAFFLNLSYAIVE
Mba1            ------------------------------------------EKNVGFAAVLNILFTFIE
Dsh1            ------------------------------------------GKALVISAWLTGVYFVIE
Ecu1            ----------------------------------------DADIRKISKVLLIILMFMLE


Sus1            VVAGLQAHSLALISDAAHNFTDALAIGLAWVGLRLQTKP-ADETKTYGYHRASVLSAFVN
Aba1            VVMGIRSHSLALLSEAGHNLSDFLALLLTWVAVYVQSRP-PSATKTFGYQRAGVLAAFIN
Mca1            ALAGWKANSLALLTDAVHNLSDVLALGLSWYAMRLTAQP-AHAAKTFGYHRAGILAALAN
Tko1            VIGGILSGSLALLSDSLHNFSDSMSILASYLAIKIGERE-KNEKYTFGYKRAEILVAFVN
Pfu1            IIGGIVSRSLALFSDSLHNFSDSMSILTSYIAIKIGERE-KNEKYTFGYKRAEILVAFVN
Fba1            VVGGLISGSLALLSDALHNFSDVISLIISYIANKLVKRK-ASIKRTFGYKRAEILAAFIN
Nsp2            IVGGIYANSLSLLSDAMHNLSDVLSLVIAWAANTIAAKP-STKSYTFGLKRAEIIAALFN
Fno1            FIGGMLAGSLALVSDSMHNLSDALSIIILAYFAHKISLRG-ANKGKTFGYGRAKIISAFVN
Cac1            VIGGSISGSLSLISDALHNLSDTASLILSYVSIKISEKP-KNKAKTYGYKRANILAAFIN
Lhe1            FLGGILSGSLALLSDAVHNLSDVGAIILSFVAHLISRRN-RNQRKTFGYDRAETLAAFTN
Lre1            ILGGILSGSLALLSDAFHNLGDSLSIVLGYFAQHIGGQP-ENRQRTYGYRRAEILSALTN
Pmo1            ILGGIFSNSLALLSDAIHNLNDTTAILISYIARVLSKKK-RDSKRTYGYKRVETLAAFVN
Tme1            IIGGIVSGSLALISDALHNLSDTGALLISYFARKISKKP-VDNKYTYGYKRAELVASVIN
Sac1            IIGGILAGSMALISDALHNLSDFTSLLISYVALRMGERQ-PTVSQTFGYKRIEVLAALVN
Dol1            IAGGIVAGSVALISDAVHNLGDFASLVVAYGAHRAGRRG-PSLRHSFGLQRMEILAAVIN
Orf1            IAGGVVSGSLALVADAIHNLSDAAALGIALFARKVARRG-ADERMTYGYRRAEIIAALIN
Rba1            VIAGILSGSVALLSDAAHNFSDANSLLIAYIARRIARKE-ANQRYTFGYRRAELIGATIN
Oin1            IVGGVFAGSLALIADALHNFSDMASLVIAFVARKIARRP-ADARMTFGYGRIEIVAALIN
Eli1            IVGGILSGSLALIADALHNFSDAASLGLAWFARRIGRRP-ADKLMTFGYAQGEVVAALIN
Sfr1            VIGGVLSGSLSLIADALHNLSDAGAIVIAIVARKIARKP-ANSQMTFGFKRAEIIGALIN
Pde1            ILGGYVADSTALIADGVHNLSDALALVLAFGARRLARRP-ASPGMSYGWGRAEVVAAFVN
Pcr1            AIGGWLTGSLALLSDAGHMLSDAIALGATLMAFKIGE-KAATHQKTFGYKRFEILVASVN
Bli1            VIGGILTNSLALLSDAGHMLSDAAALGFSLLAFKIGE-KAASASKTFGYRRFEILAAFIN
Sau1            IIGGLLANSLALLRDGIHMSSDTFSLGVALVAFIYAE-KNATTTKTFGYKRFEVLAALFN
Sha1            IVGGFIANSLALLSDGLHMFSDTVSLGVALVAFIYAE-KNATSSKTFGYKRFEVLAALFN
Ssa1            IIGGFLANSLALLSDGLHMFSDTISLGVALLAFIYAE-KYANKNKTFGYKRFEILAALFN
```

```
Bsp2            AIGGFLTNSLALLSDAGHMLSDSISLAIALLAFVFGE-KAASYSKTYGYKRFEILAAVLN
Ccu1            IAGGLATNSLALLSDAGHMFSDAAALALSLFAFKFGE-KKGTLQNTFGYKRVEILAAAIN
Ame1            IIGGWLTNSLALLSDAGHMFSDALALALSLIAFHLSE-RPVNGRQTFGYKRFEIIAAAVN
Gka1            FFGGLITNSLALLSDSGHMLSDASSLVLSLVAIWFAT-KPASPNKTYGFYRFEILAALFN
Emi1            LAGGLISGSLALLSDAGHMFSDAFALGLSLTAVIAGQ-RAATKTKTYGYRRFEVLAAFFN
Aae1            FLGGLLTNSLALLSDAGHMLTDAVSLSIALVAQYLAL-KVKTKRTTYGLYRLEVLAALVN
Nsp1            WTVGLWSQSLSLQADAGHMFSDIAALVISLFSTYLAQ-QPAKGKATFGNQRVEVLAALLN
Msu1            FIGGYLFNSLTLMADAGHMANDSLSLFLALVALFLSA-KAQK------------WFALLN
Cdi1            LVGGALAKSLALMSDALHMLSDSTGLIIALIAVVIGR-RKATSQATYGYKRIEVLAALVN
Cje1            LIGGLITGSVALLADAMHMLSDAAGLIIAVVAIFIGR-RSATAQATFGYRRVEVLAALVN
Tva1            LITGIITKSLSLQSDAWHMLSDEMSLIIGLVAHEKAK-KPPTKKYTFSLARAEVIGGFTN
Ath1            FVAGFMSNSLGLISDACHMLFDCAALAIGLYASYISR-LPANHQYNYGRGRFEVLSGYVN
Vvi1            FVSGFMSNSLGLISDACHMLFDCAALAIGLYASYISR-LPANSQFNYGRGRFEVLSGYVN
Clu1            LFYGVLTNSLGLISDGFHMLFDCSALVMGLFAALMSR-WKATRIFSYGYGRIEILSGFIN
Tca2            LVYGVWSNSLGLISDSFHMFFDCTGLLAGLAASVITK-WKANEKYSYGYVRAEVLAGFVN
Cel3            FLYGFWTNSLGLISDGFHMLFDCSALVMGLVASVMAR-WPPTRHFTFGFGRVEILSGFIN
Hma1            LAGGLLFGSVALISDALHMLFDMLAYAMAFGASYTAERFEGGEAWSYGLHRLEPVAAFLN
Jsp1            VIGGLVTGSLALLSDAAHMLTDASALAIALIAIQIAKRA-ADSRRTFGYHRFEILAASFN
Xor1            IIGAFVTNSLALLSDAAHMATDTVGLMIALVAVRLSRRP-ADARRTYGYVRLEALGALAN
Mpo1            VVGGLLTGSLALLADAAHMVTDAGGLALALLAIHYAAKA-PTAGKSFGYMRFEILAALAN
Dra1            VAYALSSRSLALLSDAGHMLTDVAALALALFAIRMGRRP-ADRQRTFGYRRTEVLAAALN
Asp2            IIGAVISGSLALLADAGHMLSDAAGVFIALLAAWIATRP-ASDQRTYGYQRAEVLAALAN
Tfu1            VVGGLAAGSLALLGDAGHMAVDAFGILVALFAIWIANQP-STDERTFGLKRAEILAAALN
Kra1            AVGAWLSGSLALLADAGHMLTDSAGLAIALIAASLAARP-ATPQRTWGYRRAEVLAATLQ
Ace1            VVAAIAGRSLALLADAGHLLVDVAALAASLWAAHLAARP-AGGVWTYGLKRAEILAAAVN
Aod1            LVGAFWSGSLSLAADAGHMVVDASGLVIALIAARLMRRP-RDEKHTWGWARSEVLAATLQ
Bad1            VVSAIVTGSLALLVDAGHMLTDMSVLIASTVTAVLMQRK-PSNKRTWGWARLEVLTAAVG
Str1            AVTAVRTGSLTLLGDAGHMFTDVLGIGMSLAALTLTSQRGKNPQRTFGLYRVEVLAALTN
Rer1            VIVGLSTSSLALLSDSAHVFTDVFGILMALTAILLAQRARSRPDRTFGMYRAEVFAALFN
Nsp3            LVVGLWSHSLALISDAGHMTADVVALGAALVATRIATRKDTSGRRTYGSYRAEVFASGLT
Rsp1            VVVGLTVNSLSLLADAGHMLTDVVGMSMGLVALVLARKGSATAARTFGWHRAEVLTAMAN
Csa1            AIGGWISGSLALLADAGHMFSDSFSLGLALFAFYMGDKA-PDKRRTFGYQRFQVLAAFIN
Oal1            VIGGLLTGSLALLADAAHMLTDFAALILAWGAFRLSRRP-ATDKRSYGYDRLQILAAFAN
Mlo1            ALGGLFTGSLALLADAGHMLADAIALGLAWYAFHLAGRP-ATGQLTYGFGRVKTLVAYTN
Lag1            LVGGWLAGSLALMADAVHMVTDAASLGLAWWAFQQSKKP-ADARLTYGRDRLPVLIAFAN
Oan1            VIGGLISGSLALLADAGHMLSDAAALALAYAAFRFGRRA-ADSKRTFGYLRFEVLAGFIN
Pat1            VIGGLLSGSLALLADAGHMLTDAAALFVALVAVRFAQRK-PNARHTFGYLRLTTLAAFVN
Neu1            AIGGWLSGSLALIADSGHMISDAAALLLALVAYRIARKP-ADSTRTYGFHRVRVLAALAN
Gbe1            IVGGLLSGSLALLADAGHMVSDVLSLLMGWAALRIGRRP-SSTRHSYGFRRLEVLAAFVN
Pbe1            IVGGVLSGSLALLADAGHMVTDAVALALALGAKYLAQRP-ASDRFNFGHQRTQVLAAFLN
Rxy1            AVGGFLTGSLALLADAAHMLSDNFSLGLALFALWLSSRP-PTPERSFGYKRAEILAALFN
Sav1            VVVGVVASSLALISDAAHMLTDAASIVLALIAMRLAARP-AKGGFTYGLKRAEILSAQAN
Asp1            AIGGWLSGSLALLSDAAHMLTDAGALGLALVAAYLATRP-ANDKRTYGYRRAEVLAAQLN
Mfe1            LIGGLISNSLALLADAAHMSSDVIALALAAFAGRLALKP-AHSGMTYGYGRARVLSAQLN
Sce1            AAVGWWSKSLALLADAGHMLADAAALGLAIIAQRIAAQA-RTRERTYGFRRAEVLAAFAN
Cbu1            ALGGYFAHSLALLGDAGHMASDAVALGIAAFASWIALRP-PSHKHSYGFGRAEVIAAWAS
Asp3            ALAGLWSGSLALLGDAGHMVTDAMALGLAALAARIALRP-ASTRHSFGLPRVEALAALAN
Mfl1            AVGGWWTGSLALLSDAGHMVSDAFALALAWLGSWIARKP-ATRKHNFGLVRAEVIVALVN
Cvi1            ALGGWWSGSLALLSDAGHMLTDSLSLLLALWAARIGRRP-ATERLSFGHGRAEVLGALLN
Psp1            GAAAYFANSLALISDAGHMVTDAAALGLALLAQIIARRP-PSPRHSFGFGRAEALAAFVN
Tca1            VIGGYLSNSLAIASDAAHLLTDFASFMISLFALWMATRP-STKTMHFGWYRAEVIGALTS
Aae2            IVGGIYSNSLAVATDAAHLLADLASFMISLFALWVAARP-STKRLSFGWHRAEVIGALTS
Cel2            VIGGVLAGSLAIVTDAAHLLTDFASVLISLFSLYIARRP-PSQKMSFGFHRAEVLGAFFS
Tca3            LIGGFIAGSLAVMTDAAHLFSDFIGFLISLLSIWVARKA-PTRNMTFGYYRAEVLGAFLS
Orf2            VVGGYLAHSLAIMTDAAHLLADVGSMMGSLFSLWLSTRP-ATRTMTFGWHRSETLGALAS
Bta1            VIGGYLAHSLAIMTDAAHLLTDFASMLISLFSLWVSSRP-ATKTMNFGWHRAEILGALLS
Xla1            IVGGYIAGSLAVVTDAAHLLVDLSSFFISLGSLWLSSKS-STMRLTFGWYRAEILGALMS
Cel1            FVGGFWAQSLAIMTDAGHMLSDLLSFIISIFAIRCARLP-ASKRLSFGYERAEVLGALTS
Tth1            FVGGYLSNSLAIMTDAVHLLSDVVGFLVTILAIYISRLK-ANSTMTYGYHRAEIIGAMIS
Ddi1            IVGGYFANSLAIMTDAAHLLTDIGAMFLSLFAMWISQHP-PTSSMSFGFHRAEILGALVS
Ath2            VVGGIKANSLAILTDAAHLLSDVAAFAISLFSLWASGWK-ANPQQSYGFFRIEILGALVS
Dsp1            FVGGLASNSLALLSDAGHVLTDVLALSLSYFALMQAKRP-SNFKMTFGYHRIGVLVAILN
Sau2            FVGGLVSNSLALLSDSFHMLSDVLALGLSMLAIYFASKK-PTARYTFGYLRFEILAAFLN
Cje2            FIYSILSNSLALLSDTLHMFSDVFALALSFLAIIAVEKW-QDHQKTFGYFRLEVLVAFIN
Cfe1            FVYALITNSLALMSDTLHMLSHVIALGLSWFAIWAASSF-SSEQKTFGYHRLETIAAFIN
Abu1            FFYGFLSNSLALISDAIHMFTHSFALIVSLFAIIIASKT-APISKTFGYYRAEVLAAFIN
Kst1            VIGGIITNSLALLSDAGHMLTHLFALLISLFAIMFASKP-PTQRKTYGFYRLEILAALLN
Lbo1            LFGGIQSGSIALLADAGHIITDVIALSLSLIAVLLASQK-PNHRFSFGYYRIEILTSLLN
Pae1            LIGGWVSGSLALMADAGHMATDLFALSISYLAIRLSARP-STKKRSYGYFRIEIIAALIN
Aph1            TIGGFVSHSLVLLSDAGHVFIDLVALILSFAAYRLATKE-ADEQRSYGYHRFQVMAAFVN
Wen1            IVGGIISHSLALLSDAGHMLTDLFALVLSWLAHRFSAKK-SDLQRSYGYHRLQIIAAFVN
Mbo1            IIGGYLSGSLSLLGDGAHMLQDVVALGLSLGAMTMAERL-PTPTRTFGYHRLEIAAAVIN
Mth1            LLGSWWTGSLALLSDAAHVFLDLFALGLSWFAIRISALP-ASERYSYGFHRFEVIAALAN
```

```
Gbe2            LAGGLWTNSLALLSDAAHVFLDLFALLLSLGAIKLASYP-VSETRTFGWHRMEVFASFIN
Chu1            AIVGLSVHSLSLLSDAGHNLADVASLALSLLAFRLLKIK-SNESYTYGYRKTSILVALFN
Lin1            AGIGLTSGSLALLSDAGHNLMDVSSLLLAWIAIKLQEKK-PFPGFTYGWKKSTILVSLLN
Rpa1            AAFGWLGNSMALLADAGHNLSDVLGLAVAWIAAELSKRP-PSQRYTYGLRGSSILAALFN
Van1            ATYGFITDSLALLADAGHNLSDVVTLALAWGAFVLAKKA-ATEQRTYGFRKVTILASLTS
Gox1            VLWGVWAHSLSLLADAGHNLSDVLGLAGAWLAQVLATRP-SSARFTYGLRRSTILSALAN
Afa1            GFYGWRANSLALLADAGHNLSDVGGLVLAWVAYGAAKMR-PNQRHTYGWRKASILASFVN
Bph1            GMYGVLANSTALLADAGHNLSDVLGLLLAWGATWLATRR-PSARYTFGLGGSSILASLLN
Lpn1            IIFAYLANSTSLLADAFHNLGDVLGLILAWVATGLMKRK-PTHKATYGFKKTSILAALAN
Rgr1            VVYGFIAHSVSLLSDAVHNFADVLGLLMSWGASVLIKRR-ATQRYSYGYKKLTILAALAN
Lme1            LVFAKLAHSTALFADAFHNLSDVLALMIAWLAVVVFGLQ-ATKRHTYGWHNMSILASVFN
Sde1            FIGGWLTNSTAIMADAVHDLGDSLSIGSAWVLNKLSNKQ-ANRKFSYGYKRFSLLGALIN
Ilo1            FIGGFLTNSTAILADAVHDLGDSLSLGLAWILNKLGKKQ-ANQHFTYGYKRLNLAGAFIN
Asp4            IIGGIWINSVAIISDAVHDLGDSVSLGLAWYLDKKSKKS-ANHQFSFGYTRFSLLGALIN
Mma1            LIGGVLTNSMAIMSDAIHDLGDTIAIGSAWFMERYSEKG-CDDRYNYGYKRFSPLSAFIT
Pgi1            LVGGIFTGSVAILSDALHDFGDSISLGIAWRLQKLSEKG-RDRSFSYGYKRFSLLGALLI
Bsp1            FIGGVMTNSMAILSDAVHDLGDSLSLGSSWYLEKLSGKG-RNSRYSFGHRRFSLLGALIN
Efa1            FFFGSLFNSVSIMSDAVHDLGDSISIGFAWFFQGYSEKQ-QDEQFTFGYNRFSLLGALIT
Lla1            FIFGVIFNSTAILSDAVHDTGDAVAIGLAWFFQKFSKRR-EDKHFSFGYQRFSLLGASLT
Sgo1            FIAGGIFGSSAVLADSVHDLGDAIAIGMSALLETISNRE-EDGKYTLGYKRFSLLGAILT
Mba1            LIGGFLTNSLALIADALHDFADSFALIIAWYAEKKAKKP-ATSKMTFGYRRLSLLSATFT
Dsh1            LAVGLWTGSIAVLSDAFHTLSAVGGVLVAITAQRIARRP-ADSTRSFGWYRAEIIGALVN
Ecu1            LWGHWKTNSLSLLADSLHLLVDIFGFIVSLLSLSWAKKP-SNKRMTFGYHRIEIIGSMVS
                        *  :  :  *                                       .

Sus1            ALTLVALSAWILYESVLRLRNP----EAVQESVMMAVAGLGLVLNGGIMLALRAS-----
Aba1            AASLVLIAFFIFYEAGRRLYAP----SDVEPRTMMWVAACGVVMNGAIALMLLR------
Mca1            SAGLVGVALYILYDAYVRLLSP----APVQADILIGVGGAALLVNAFTAWLVHHG-----
Tko1            SAVLVGVALFLLVEAYKRFKNP----EPIDGPLMLGVALIGLFANLISVLLLHEH-----
Pfu1            SAILVGVALFLIVEAYKRFKTP----EPINGPLMFSVALIGFLANLISVLLLHEH-----
Fba1            ASTLIIVAILLIFEAVERFQNP----QEIESNLVIWLSIVAILGNGFSVLLLKRN-----
Nsp2            ASLLLGIAIFLVIEAFHKFFHP----QSVDSLWVIWLGGLSILLNTLSVLLVKKD-----
Fno1            SVTLMVITVLLIKEAVLRLIKP----EFVHSDTVIIVGTIGLIANILSMIFLKGH-----
Cac1            SAALIGISIFLGVQALEKLTSL----KKINANIVIVVALIGLLGNFLSVIILKKG-----
Lhe1            GVVLIVISVVLFVEAIQRFWKP----EHIHGGIMLVVSIIGLVANIISMLAMHHD-----
Lre1            SIFLIVISVFLIIEAIKRLEHP----QHINGGIMLTVAVIGLLANFISAALLHAG-----
Pmo1            TEILMVIAVYLLIEGINKLSNP----SIIQGNIMLIVAFIGLAGNLATAYLLHSG-----
Tme1            ITVLLSISFSLILEGIKKILSP----VQINTSIMLIVAYVGLVGNLLTAILLSSH-----
Sac1            VSLLYGVAIFIAIEGWQRLLAP----QVIKGQLVVWIALAGLAGNAFSAVLLHAG-----
Dol1            SALLGGAALFIATEALDRLANP----QPIRLGIVAWLALAGLAGNGLSAWLLHRD-----
Orf1            LTTLIVIGVYLVYEAILRFFAP----EPIVGWIVVVVAGVALAIDLVTAALTYAG-----
Rba1            LTLLAVVGCYLVYEAVHRFFDP----QPIIGWLMAVTAAVALVVDLGTAFLLWAM-----
Oin1            YTTLILVGIYLIYEGGMRMIDP----PAVAGWTVVILGGVALVVDTLTALLTYSM-----
Eli1            LTTLLIIGFYLIVEAINRFAAP----QPVEGWTVIWVAGIALVIDLVTAFIVYRG-----
Sfr1            STTLIILGIYLIYAAVEKYFNP----QPIDGWIVVWIATIALVIDSITAWLTYQAG----
Pde1            YIALIAISVWLGIEALGRMVEP----PQVAGGLVMALAGFALLVDMATAALVWRA-----
Pcr1            GATLVIIALMIFYEAIKRFNSP---PEIATQGMLIVATIG-MLINILVAWLMHRGSRSGD
Bli1            GITLLLISLYIFWEAYNRFFSP---PEVAGRGMLAIATVG-LFVNIAAAWVIMKGDTSG-
Sau1            GVTLFVISILIVFEAITRFFVP---SEVQSKEMLIISIIG-LIVNIVVAFFRFKGGDTSH
Sha1            GVTLFVISIVIIVEAIKRFFAP---PEVQSTEMFIISLIG-LIVNIVVALIMFKGGDTNH
Ssa1            GVTLFVIGIIIIIEAIERFFNP---VEVQSTEMFIISVTG-LIVNIIVALLMFRGGDTSH
Bsp2            GVTLIGIALFIFYEAIERFANP---PEVATTGMLIISTIG-LLVNILVAWIMMRGSDTKD
Ccu1            GIALIAIAVLVVIEAIRRLQNP---PEVASLGMLVISAVG-LAVNIVVALYMARGADTKE
Ame1            GLALMVIAVMIVYEAIGRFVNP---PDIATRGMLIISTIG-LL-----------------
Gka1            GVTLFVIAGFIIWEAIERFYNP---PTVASGSMMLIASIG-LFANLLSAWALMKKGDVKN
Emi1            AITIFLIAVFILKEAVVRIQNP---APILSGYMFIIAVIG-LLVNIAVLMILRRREIKD-
Aae1            GVFLLGLIGYIILEAIHRFENP---EPVKP-QMIYIAFAG-LIVNLVVGYILLKHSEEN-
Nsp1            GLSLLAIATFITWEAIQRWQHP---GVILGLPMLVVAVMG-LIVNLLNISLLHPHSHND-
Msu1            GTSLVFVAVMILIEAFKRWQAP---TEMAALPMMTVAIIG-LLVNILVAWIMLKSDQEN-
Cdi1            ALSVTFITGWIVLEAIRRLSSH---TVIDT-RTTMVIAIIGLVFNIVGAVVLHGHSHEGV
Cje1            AVTVLGISAWIVVEAFQRLSEP---VEIMA-GPMMIVAVIGLLANIISAWILNRQREHSV
Tva1            AIFLLAVCMTILFEAIERFIKV---EEIVEPKAFLIVGILGLLVNAIGIFIFHDH-HHSD
Ath1            AVFLVLVGALIVLESIERILDP---QEIST-NSLLVVSVGGLLVNIVGLIFFHEEHHHAH
Vvi1            AVFLVLVGALIVLESFERILEP---QEIST-NSLLTVSIGGLLVNVVGLIFFHEEHHHAH
Clu1            GLFLMVIAFFVFVESVARLIDP---PELDT-HMLTPVSVGGLIVNLIGICAFSHAHNHNH
Tca2            GLFLLFISFFIMSEAVERAIEP---PEVKH-ERLFLVSVLGLLVNLVGIYAFQHGHGHSH
Cel3            ALFLCVIALFILIEALERLFDP---PNINT-DRLLFVAISGLIVNLFGMYSLG-EHGHSH
Hma1            GVLLLPMVGYIVWESYQRFLEP---VAINP-ELTLIIATGGLLVNIGSVYVLQGGEMSLN
Jsp1            AIMLFFVAMYILYEAYRRFKS--P-IEIQSM-GMLIIASLGLIINLISMKLLSSGK---D
Xor1            GALLFAVGGYILWEAAQRVRA--P-QDIAYG-GMLLIAGFGLVINLIAMKLLHAGS---G
Mpo1            AVVLLGVTAYILYEAYRRFVE--P-TEILGW-PMMLVALVGLGVNLASMKLLSGGS---S
Dra1            AGALFAIGLYILWEAVQRFRQ--P-VEVQTT-SMLVVAVAGLVVNLLSARLLAGG-----
Asp2            ALVLIVISVLIFTEAVRRIGS--A-PEVQTD-VMLYAAVLGAVANLVSLLILRSA---QK
```

```
Tfu1            ALLLFALCGFIGYEAVRRLLD--P-QPVSAS-TMVVFSTVGLLANLIALGLLRRG---QA
Kra1            AAILLAVGVFIIVEGIRRLLQ--P-PEVAST-AMIVFGVVGLLGNAASILVLTGGQKGQN
Ace1            GVTLVALALVIVVEAVRRLTS--A-PSVVGS-IVLLTAVAGLIVNAVATAVLARG---QH
Aod1            AGMLLIICAIVAWEGAWRLLS--P-PEVEAG-PMLLVGIIGLASNVVSLAILAGG---RD
Bad1            ALVLLIVGIYALVEAGMRLFGGAA-DEVHDVRLLLFMGILGLAANVGSIFILAGQ---SE
Str1            ALMLSAVSIYVLVEAIRRFSG--P-PEVDTG-PMLVVAVLGLLANIVAFVLLRPG---AK
Rer1            ALLLFGVAGWVLYEAAGRLSD--P-PEVPGL-PVSIVAVVGLVMNIAAFLLLRSG---AK
Nsp3            VLIMLGVAVYVVVEAVGRIGA--E-PEVQTG-AMLVVGAIGLAVNIASMLLLREG---ST
Rsp1            AAMLLAVAVWVFYEAISRIGD--A-PELPGM-ALILTATVGLAANLVVMLMLRAD---SK
Csa1            GLTLLGIAVWILIAAVQRFFQP---VEVMAA-PMLAIAVLGLLVNVVVFRILHGG---DH
Oal1            GVTLSILVVWIACEAVMRLFDP---NEIEAT-GMMVIAVLGLIVNIAAFGVLHGA---DT
Mlo1            GIAIFVIALWIVYEAWQRLLTP---APVLGG-PMLVVAILGLLVNIGSFLVLHGG---DR
Lag1            SIFLLVVTGWICVEAAGRFMEP---ETVLAG-PMFIIAVIGFLVNIGAFFILQRG---GD
Oan1            AITLFAIVVWIVYEAWQRFQEP---HVILAG-PMLGVAIAGLLINILVFWILTRG---DT
Pat1            ALTLILITAFIFWEAIQRFYDP---QPVAGV-PMLLVAIAGLLANIVAFWLLHHGS--EE
Neu1            GVTLLLLVVWIILEAIGRMHTP---TEILAG-PMLIVAVIGLVINLVGAWVLWSG---NK
Gbe1            GSSLLIVSIWIVIEAVRRLLNP---QPIGGG-LMLAVAAVGLVANAISLLILNEG---RE
Pbe1            GVGLFLLIAYLLVESVHRMAEP---ATIEAS-TMLNVAVIGLLANIAAFWVLQNG----T
Rxy1            GVTLVAVSLWIFYEAYRRLLEP---REVMGG-WVAAVAAAGLLVNAAAAWVLLR-P--RS
Sav1            GLTLLLLGVWLAYEAVRRLVEP---PDVEGG-LMLITALAGIVVNVAAAWCISK-A--NR
Asp1            VGALLVLSVWIVWEAVERLRQP--HAAIDLR-LMASVAVVGLAANLAILWFLHE-----E
Mfe1            GFALWFLSGWITWEATGRLSAP---PAVHGG-MVIAIGAIGLIVNLVILTWLHG-----E
Sce1            GVALALTAIWIFIEAAHRWRAP---QAVHAE-ALAITAALGLLVNVVAALLLSTGE--HG
Cbu1            SLLMVIIALAVIVEAIKRIQQP---SSVHSV-PVMLIAVFGILLNVLLAGLISR----SE
Asp3            SLFMLALVAALLWQAAARLAAP---RDISGE-TVTLVALAGLALNLAVAWLLTR----GE
Mfl1            ALLMLAVIGGIAYEAVQRIADP---RPVQGG-EVMLIAFIGMLVNIVVALMLHK----GH
Cvi1            SLLLFGLSAFIVAEAVERLLRP---HAVNGM-GVMLIAAIGLAVNVLAARVLSR----GA
Psp1            SIAMLALVLWIVIEAISRFANP---HKVDGL-TVTVVAAIGLLMNVVVAWVLSR----DT
Tca1            VLLIWVVTGILVYIAVQRVIHK---SFDIDSKVMLITSGIGVIVNLIMGLTLH-----QH
Aae2            VLMIWVVTAILFYLAVLRTINQ---DFEVDGQAMLITSGLGILVNVIMGATLHG----GH
Cel2            VFLIWIVTGVLVVLAIMRIVSG---DYEVEGGIMALTAALGVVVNLVMLALLYF--G-GH
Tca3            VLTVWLLAGVFAVLAINRLLKK---EYDIEANTMMLVASLGLVVNIVMGAVLFG--F-CH
Orf2            VVSLWMVTGILLYLAFVRLLHS---DYHIEGGAMLLTASIAVCANLLMAFVLHQ--A-GP
Bta1            VLSIWVVTGVLVYLAAERLISG---DYEIKEETMLITSGCAVVVNIIMGLILHQ--S-GH
Xla1            IITIWLVTGVLVYLAIERIIRP---DYTIDGTVMLITSACALGANVVLALILHQ--S-GH
Cel1            VIILWVLTTVLVVVAIQRIVNN---EHEVDADVMLITAGVGVLFNIVMGLVLHFGTG-GH
Tth1            ILIIWVLIAWMFVEGIERFFK----PPEIQGTTMLILACCGLFFNLVLMKILET----KL
Ddi1            VLMIWALTGVLVYEAIQRILYP---PDAVDGKIMFIIASCGLFINIIDAIILHWGSG-GH
Ath2            IQMIWLLAGILVYEAIVRLNNG---SGEVEGSLMFAVSAVGLLVNIAMAILLGHDHGHGH
Dsp1            SISLIGIAVFIFYESFQRFQSP----PEINAWLVLPVALLGLATNLAVALILHS--E---
Sau2            GLALIVISIWILYEAIVRIIYP----QPIESGIMFMIASIGLLVNIILTVILVRSLK---
Cje2            ALTIILSALFIIYEAIEKFINP----KEIDAKTMIIVAILGFLVNGINALMMFKG-----
Cfe1            SILIAVFGLFIVYEAIVKLINP----EPIEIGTMLIVAAIGLGVNITTGLIMLKG-----
Abu1            GITIVLSIIWIIYEAIERFLNP----QIIDIKTAMIVAIIGLIVNIITGVILMQG-----
Kst1            GIILLLITVWIFYEAYHRFVTP----EAISSGMMFFLAIIGLLANISCVYILKSG-----
Lbo1            SILIFGISFYIFYEATERFQNQ----KEVLSFPMIFYSVSGIVLNLISAWILFRF-----
Pae1            GVILCITALFITIEAWKRISLP----KEIDSAQMLVFGIIGLTANIASAIMLHRE-----
Aph1            GISWFVIAALIVIESIKRFVNP----VEVHGHLMLPVAVVGFAANIFVFCMMYRR-----
Wen1            GLTLFLIAVIIIIESIKRFIFP----VNVEWKVMLIIATLGLVSNIIVFFILHSK-----
Mbo1            GLLLIGVSALIILEALARFSHP----SPVNSTLMLAVALVGLVANAISAFVLHG------
Mth1            GLTLGFVALVILIEAYRRLLQP----EPVKGLDLLLIATFGLIVNLIVAFVLSRD-----
Gbe2            GSTVFLIAGIISYEALVRFIHP----EEVKSLEMLIIAFVGLVMNLIAAGALHSH-----
Chu1            SMILIASIGAIFYEAAHRFFNPE----PLPGQTIAVVAGIGILINAATALMFLRHK----
Lin1            SILIFGTVGLIVYESIEKLSKPS----SVPGGMIAAVAFVGVLVNFSSSFLFHKSK----
Rpa1            AVFLLIAVGAIGWEAIARLFAPE----PVGSVTMMVVAGIGILINGATAWLFFSGR----
Van1            ALLLLFALGAIAWEAVLRFSEPR----PVAGTTVMVVAFIGVIINAATAMLFAKGQ----
Gox1            ATILLLVTGGIVWESVLRLFSHQ----NVQGEVISWVALVGIAVNAVTALLFMKGA----
Afa1            AVILLVAMGSLASEAISRLGDPG----TPNAQTIIIVAGVGVLINAVTAWLFMAGS----
Bph1            AGLLLFACGVIVAEAVGRLIHPS----PVAGFDVFIVACVGMVVNGVSAWLFMRGQ----
Lpn1            GALLVFTCGIIATDAMYKLFSPT----EIQAVSVMIVAAIGIVVNFSTALLFLRG-----
Rgr1            ALLLVLTSALIIYESVNRLYNPK----EINEMIIMIVAFIGMVLNGSTALLFLREN----
Lme1            TILLIFAVITIFYESIRNLILPEN--TVTTGWLVMVVAAVGIVINFATAMLFKASGVPDE
Sde1            GLVLVLGSVWVLTEAIPRLSSPQ----MPVTEGMFVLAIFGIMVNGVAAYKLSSG-----
Ilo1            AVVLIAGSAWVLVEAIPRLWNPQ----MPVADGMIALAVVGITVNGFAAYKLSEG-----
Asp4            ALILIIGSIFVITEAVTRILNPE----VSNAQGMIIFALIGVAVNGYAAWKVSHG-----
Mma1            SVILIVGSVFIFIETIPRLIHPE----AVNAKGMLLLAILGVTMNGLAVLRLKSGN----
Pgi1            SIILLVGSVFVIIAAVQRFSMPG----EPKAGGMLILAIFGLVVNGLAALRLKG------
Bsp1            GIILIVGSLFVLYEAIPRLFHPQ----HPNAQGMVWLAILGIILNGIAAYRLHKG-----
Efa1            GVVLIGGSFFMIYRSIPRLIDPQ----PVNYSGMFWLSLVAIGLNGYAAWILSKG-----
Lla1            SVILITGSFIVLFEALPRFFNPQ----PVETTGMLGLAIFAIVANGFGAWLLARG-----
Sgo1            AVILMIGSVLVILENITKIVHPQ----PVNEEGVLWLGIIAVSINVLASLVVRKG-----
Mba1            IIVLVAGSLFVLTQAIPRLISPE----PVNAEGMVLIAVIGVTINSLGYFRLKKG-----
Dsh1            GGFLLGMALLVIWMGAMRLGDP----IHLATGPMLWVAFGGLVTEVVSLALMWQSS----
```

```
Ecu1            IGLIWAAVGYLVIESFHKYLHP----AEIDGGMFFGIAVVGFFVNCICIYVLHYDEYQHK
                                 .

Sus1            ------------------------------------------------SKNDINIRSAFV
Aba1            ------------------------------------------------TRRDLNIRSAFL
Mca1            ------------------------------------------------SHDDLNMRSAFL
Tko1            ------------------------------------------------AHESMNVRSAYL
Pfu1            ------------------------------------------------SHENINVRSAYL
Fba1            ------------------------------------------------AEDNMNMKSAYL
Nsp2            ------------------------------------------------SEKNINIKAAYL
Fno1            ------------------------------------------------SEKDINIKSAYL
Cac1            ------------------------------------------------AEKSLNVRSSYL
Lhe1            ------------------------------------------------AKGNLNVRSTFI
Lre1            ------------------------------------------------SEDSLNVKATYL
Pmo1            ------------------------------------------------SKTNLNVRATFV
Tme1            ------------------------------------------------SKENLNLKSAFL
Sac1            ------------------------------------------------AKTNINIRSSFL
Dol1            ------------------------------------------------SARNLNARGAFL
Orf1            ------------------------------------------------AKESMNIRAAFL
Rba1            ------------------------------------------------SKGSLNVRAAFI
Oin1            ------------------------------------------------QKGSVNIRALFL
Eli1            ------------------------------------------------AHDSINMKAAFL
Sfr1            ------------------------------------------------AKNNLNLRAAFI
Pde1            ------------------------------------------------AKESVNIRAAFL
Pcr1            THGHDHDHGANEGTATVKTSDD-------------------------KEPVNLNMQSAYL
Bli1            ---------------------------------------------------NLNMRSAFL
Sau1            ---------------------------------------------------NLNMRGAFL
Sha1            ---------------------------------------------------NLNMRGAFI
Ssa1            ---------------------------------------------------NINMRGAFL
Bsp2            ---------------------------------------------------NLNMRGAFL
Ccu1            ---------------------------------------------------NVNMRGAYL
Ame1            ------------------------------------------------------------
Gka1            ---------------------------------------------------NVNLRSAYL
Emi1            ---------------------------------------------------NINVKGALL
Aae1            ----------------------------------------------------INIKSALL
Nsp1            ----------------------------------------------------LNLQGALL
Msu1            ----------------------------------------------------LNIKAAYL
Cdi1            N-----------------------------------------------------VKGAYL
Cje1            N-----------------------------------------------------VQGAFL
Tva1            N-----------------------------------------------------IQGIFL
Ath1            GGS-----------------------------------------------------GIFL
Vvi1            GGSCSHSHSQSHSHSDLHHHSHACGGHD--------------HTKRNHKHIDHNMEGIFL
Clu1            GASQGSCHSSDHSHSHHMHGHSDHGHGH--------------SHGSTGRGMNANMRGVFL
Tca2            G---GGGHGHSHGHNHNSHGHS-HDHTL--------------DIDIAASGNSQIMKGVFL
Cel3            GGGSSHGHSHGGGGSHGHSHGG---------------------------GGNANMQGVFL
Hma1            E------------------------------------------------------RGAFY
Jsp1            --------------------------------------------------ASLNIKGAYL
Xor1            --------------------------------------------------ESLNVKGAYL
Mpo1            --------------------------------------------------ESLNVKGAYF
Dra1            --------------------------------------------------EGLNLRAAYL
Asp2            --------------------------------------------------ESLNVRGAYL
Tfu1            --------------------------------------------------ESLNLRGAYL
Kra1            --------------------------------------------------SNLNMRAALL
Ace1            --------------------------------------------------RSLNIRGAFA
Aod1            --------------------------------------------------ANLNMKAAFL
Bad1            --------------------------------------------------DNMNMKAAFL
Str1            --------------------------------------------------ESINLKGAYL
Rer1            --------------------------------------------------ESINVRGAYL
Nsp3            --------------------------------------------------ESLNVKGAYF
Rsp1            --------------------------------------------------DSLAVRGAYL
Csa1            --------------------------------------------------ENLNLRGALL
Oal1            --------------------------------------------------DNLNIRGALA
Mlo1            --------------------------------------------------ESLNMRGAIL
Lag1            --------------------------------------------------GSLNMRSAIL
Oan1            --------------------------------------------------EHVNIKGAIL
Pat1            --------------------------------------------------KNINVRAAAL
Neu1            --------------------------------------------------EDGNLRGALL
Gbe1            --------------------------------------------------SSLNMRGVWL
Pbe1            --------------------------------------------------DDVNMRGAVL
Rxy1            --------------------------------------------------ESLNLQGALR
Sav1            --------------------------------------------------TSLNVEGAYQ
Asp1            --------------------------------------------------HTLNARAAFL
Mfe1            --------------------------------------------------HDINSRAAFW
```

```
Sce1            --------------------------------------------------HNINTRAALA
Cbu1            --------------------------------------------------KTLNIRAVLL
Asp3            --------------------------------------------------GDLNTRGALL
Mfl1            --------------------------------------------------SSLNSRAALL
Cvi1            --------------------------------------------------HSLNSRAALL
Psp1            --------------------------------------------------KSVNTRAALV
Tca1            GHSHG-GGSTH-------------------------------------SHDNINVRAAFI
Aae2            GHSHSSAGSAHGHAHGGP------------------------------SEENINVRAAFI
Cel2            SHSHG-GGSSHGHSHGGG------------------------------NGDNINVRAAFI
Tca3            NHSHG--LSDQ-------------------------------------SNSNINVRAAAA
Orf2            PHSHGSRGAEYAPLEEGPE--------------------------EPLPLGNTSVRAAFV
Bta1            GHSRNPEHSHNASQEQG----------------------------------SPSVRAAFI
Xla1            GHSHAGGKHEHMASEYKP-------------------------------QTNASIRAAFI
Cel1            GHTHG-GHSSHGHAHDGK---------------------------------NVNVRAALI
Tth1            ETVTEESQKEELLSEFSIK--------------------------QDVEKENMSMRAAQI
Ddi1            GHSHGGGHGHSHGIGGGTQKKKSKKNRLLNNQGQDIEDLGGENGKNKKGVRNINVHSAYI
Ath2            GHSHDNGHGHSHDHGHGIAATEHH-----HDSGHDESQLSDVLIEQKK-QRNVNIQGAYL
Dsp1            ------------------------------------------------QKENLNIKSAFW
Sau2            ------------------------------------------------QEDNINIQSALW
Cje2            -----------------------------------------------ANLENVNMKSAFL
Cfe1            ------------------------------------------------DMGNLNIKSSFA
Abu1            ------------------------------------------------DKDNINLKSAFI
Kst1            -----------------------------------------------EHGHSLNVKSAFL
Lbo1            ------------------------------------------------SAENINIKSAYI
Pae1            ------------------------------------------------QKNSVNVKAAYI
Aph1            -----------------------------------------------GE-HNLNIRSAVL
Wen1            -----------------------------------------------CE-SNINIKSAVL
Mbo1            ------------------------------------------------S-HDLNTRSAFL
Mth1            -----------------------------------------------GHMDDLNLRSAIL
Gbe2            -----------------------------------------------SH-DDLNVHSAFL
Chu1            -------------------------------------------------EKDLNVKSAYL
Lin1            -------------------------------------------------DQDINMKGAYL
Rpa1            -------------------------------------------------DNDLNIRGAYL
Van1            -------------------------------------------------KHDLNMKGAFL
Gox1            -------------------------------------------------SSDLNVRGAFL
Afa1            -------------------------------------------------RNDLNIRGAFL
Bph1            -------------------------------------------------GDDLNIRGAFL
Lpn1            -------------------------------------------------SDDLNIRGAYL
Rgr1            -------------------------------------------------CSDLNIKSAFL
Lme1            HGHHH--------------------------------------------EQDLNAKTAYI
Sde1            --------------------------------------------------TSINERVLNW
Ilo1            --------------------------------------------------KTLNERVINW
Asp4            --------------------------------------------------STQNEKVVSW
Mma1            -------------------------------------------------NDSINQKAVML
Pgi1            -------------------------------------------------SSSLNERAVML
Bsp1            --------------------------------------------------SSMNESILSW
Efa1            --------------------------------------------------SSKNEGMLNL
Lla1            --------------------------------------------------SSRNESILNL
Sgo1            --------------------------------------------------KTKNESILSL
Mba1            --------------------------------------------------MSQSEKVLSW
Dsh1            -------------------------------------------------KDDLNARGALW
Ecu1            -----------------------------------------------LKHKNLNIRATYV


Sus1            HMLGDALGSVAII-AGAVAIRYTGWL---RVDPVLSIVIALLIVWTAWDIIRESLNILLE
Aba1            HELGDTLSTAAVI-VGGWVILETGQS---WVDPALSFGIGVLVLWSSFGVIRDSLNILLE
Mca1            HLLGDVVSTLGAI-AAGVAIRYTGLY---WLDPAVSVLIALLILWNAKGLMAEVMDILLE
Tko1            HLLSDTLSSVAVVIGGIAIIRWDVL----WIDPLVTVLISVYILREGYEILKESVEVLME
Pfu1            HLLSDTLSSVAVVLGGIAIIKWNAI----WVDPLLSALISIYILREAYEILKESVEVLME
Fba1            HLLTDMMASVAVLVGGLLMQFYQIW----WIDSVLTFAIALYLIWMGFDLLKASTKVLML
Nsp2            HLLTDVFTSIAVVVGGLLMQFYHIY----WVDPAVSLLIAFYLIYASIDIVKESIAILME
Fno1            HMLGDAFSSVAVVLGAILIKYLNIY----TIDPILTIFIALYIGKESFEILIKSLNVLMQ
Cac1            HMLSDAMASLAVIISGVFIKYFAIY----WIDSVLTIFINMLILKSSYNILKESINILMQ
Lhe1            HMLSDALSSVAVVIGAIFIYFWNVT----WLDPVLTILVSIFVLHEAYEITMKAANVLME
Lre1            HILSDALSSVAVIIGGIILTFVNVP----WLDPALTIGVALYIAYEAWPIINQTIKILMQ
Pmo1            HILSDTFSSIFVIIGAFLIIYQKLY----IVDAIFTLMISGYIFVESVPLLKNTVNILLQ
Tme1            HILSDMFSSVGIIITGHVMSYYNIW----ILDPIITFVISGYIIIESIKILKESIRVVMQ
Sac1            HMLTDALTSLGVVVLGIIWLYRPWY----RLDTLVSWGIVALIFYGGWGILKETYQILMN
Dol1            HMVGDTLTSAAVLTGALVMRVADMP----WLDPALSLAIVAYIAWNAVLLLKEATHVLMN
Orf1            HNVADALGSVAVIVAGTLVIWFGWN----WVDPAVTLLIAGYILWHGLVEIRACIYILMA
Rba1            HNLVDAAGSVAVLIGAAAVIYLKWL----WVDSILTLLIATYILYQVWQMLPEATRILME
Oin1            HNLSDALASVAVIFGGTLIILYDLR----WVDPAITIGIALYILYLALTEIGGPIRMLML
Eli1            HNVSDALASIGVIVAGVLIILYDLV----VADLIITLIIAAYVIWQGATLLPRTVRLLMG
```

```
Sfr1            HNLSDAFASVVVIVAGSLIILYQWY----VVDLLATILISVYVVYHGIILAKQSIRILMQ
Pde1            HNLADAAVSVAVILGGALIWAFGWH----LADPVLTLLISGVILWHVGQEIGPVMRMLML
Pcr1            HVLSDLMGSVAAI-IAALLMMSFGWV---WADAVASVIVAILILFSGYRVIRDSVHILME
Bli1            HIIGDMLGSFGAI-IAGLLMLFFNWN---IADPIASAVVAALVLVSGWRVTKDSVHILME
Sau1            HVIGDLLGSVGAI-TAAILIWAFGWT---IADPIASILVSVIILKSAWGITKSSINILME
Sha1            HVLGDLLGSVGAI-IASILIWTFNFT---IADPIASIVVSLLILKSAYGITKSSINILME
Ssa1            HVMGDLLGSVGAI-IAAILIWTLNLT---IADPIASIIVSLLIIKSSWGITKSSLNILME
Bsp2            HVLSDMLGSVGAI-VAALLIMFFGWG---WADPLASVIVALLVVRSGYYVTKSAIHVLME
Ccu1            HVLGDALGSVGAI-VAAILIMSFGWG---WADAVASVLVSLLIAKSGYGLLKATFHILME
Ame1            ------------------------------------------------------------
Gka1            HVIGDALGSVGAI-IAGLVMWLFSWY---VADPIISILVALLILKGAWGVIKHTVHILME
Emi1            HVLGDILGSVGVI-IAAALIYFFGWY---IADPIISVIVAFLILYSAWKIFAETVNILLE
Aae1            HVATDTLGSVAAI-IAGIAIVFWKFY---LADPILSVAVALLILPSAYSVIKETVNVLLE
Nsp1            HIIADTASSVGVI-IAAVMIHLWDWW---WADVAISLVVATFTGVSALPLVQESIKIFLE
Msu1            HVLADLFGSVVAI-IAGLSAWLLDWQ---WVDVVASVILSALVLRSGLSVIKQAITALRS
Cdi1            HILVDLGGSVAVI-VSSLLIMTTGWM---WCDTAVSVLLAVIILPRSLSLVRSTLGILME
Cje1            HVLADMLGSVAVL-VAGGVIILTGWQ---YADVIASLVIAALVLPRAWQLMMQALRILLE
Tva1            HIIGDLLGSVAVV-ISAAVCQWTTWNGRFYLDPACSIFIFIILVYGSQGLLRRTGSILLE
Ath1            HVLADTMGSVGVV-ISTLLIKYKGWL---VADPASSIFISILIIASVIPLLRNSAEILLQ
Vvi1            HVLADTMGSVGVV-ISTLLIKYKGWQ---VADPACSIFISVLIVASVIPLLRNSAEILLQ
Clu1            HVLADTLGSIGVI-VSTVLIEQFGWF---IADPLCSLFIAVLIFLSVFPLIKDAYQVLLL
Tca2            HILADTLGSVGVI-ISAVLMQMFGWM---IADPICSMFIAILIALSVLALIKDSVVILMQ
Cel3            HVLADTLGSVFVI-ISTLLIQWFGWV---WVDPLCSLILSLLIIGSVYPLLVSSISTLLQ
Hma1            HLLGDAGGSVAVI-VSTAAVAVFDLP---IADPVAAVLIGLLVLASAGNVLRESTSILLE
Jsp1            EVWSDMLGSVGVI-AGALIIRFTGWT---WVDSVIAVGIGFWVLPRTWILLRESINVLLE
Xor1            EVWSDMLGSMAVI-IGALLIHWTSWQ---WIDPVLAVLIGLWVLPRTWVLLREAINVLLE
Mpo1            EVFSDMLGSVGVI-LAALVVMGTGWT---WVDPLIGAGIGLFIVPRTWRLLSEALHILLE
Dra1            EVLGDLLGSVAVI-AGALLIRLTGWS---WVDPLLGAGIGLWVLPRTWSLLKTSVNVLLE
Asp2            EVLGDLLGSFAVI-AAAVVIMVTGFQ---AADTIASMVIAVMILPRAWHLLRDVVDVLLE
Tfu1            EVLGDLLSSAGVV-VTALIIWLTGWH---WADSVVSIAIALFIAPRAWHLLNEALRILLE
Kra1            EVINDALGSVAVL-IAAVVIATTGWL---RADALVSLLIGALILPRTIKLLRETIEVLLE
Ace1            HLLTDVYAFAGTA-AAGLVILITGWG---RADAIASLLVAALMGKAAYRLLRDAGRILLQ
Aod1            EVANDALGSVAVI-VAAGAEWAFGWT---RADAIASLLIAALMAPRALTLLRRSVAILME
Bad1            EVMNDALGSVAVI-VSAIVLISTGWA---GFDAVAGGIIALMMIPRAFVLLRNAVRVLLE
Str1            EALSDLLGSLSVI-AAALIIAGTGWQ---LADPLIAVALGLFRLPRIWLLGRAATRILVQ
Rer1            EVMADMLGSVGVL-ISGLVTLIFGWR---YADPVIGVAIGLFVLPRAYNLGRHALRILLQ
Nsp3            EVVADAVGSVGVI-LAGLLVATTGQT---WWDTGVAVAIGLFVAVRAIVLGRQVLAVLGQ
Rsp1            EVLADAVGSVGVL-IAGGVVLAFDWA---YADIIVGVLISLWVVPRAMKLAAASLRILTQ
Csa1            HVMGDLLGSVAAI-VASLVIMATGWM---PIDPLLSMLAAALILRGAWKIVRRSGHTLLE
Oal1            HVMGDLLGSVAAI-IAAGVIWWTGWT---PIDPLLSLLIAGLIGISAWRLVRDAGRVLLE
Mlo1            HVLGDLLGSAAAI-VAAVVILVTGWT---PIDPILSVLVSLLILSTAWSLMRAAAHVLLE
Lag1            HVLGDLLGSVAAI-AAAVVIYFTGWY---PIDPILSVFVALLIVRSAVSVMRQSAHILLE
Oan1            HVLGDLLGSVAAV-SAAIVIMYTGWT---LIDPILSVFVSLLILRAAWNLLGKSIHILLE
Pat1            HVLGDLLGSVGAI-AAAIIILYTNWT---PIDPILSILVSCLVLRSAWALLKESIHELLE
Neu1            HIIGDLLGSVGAV-AAAVGIMFTGWT---ILDPILSVLVALLIIRSAWVLVKDSVHVLLQ
Gbe1            HVLSDLFGFAAAI-LAALIIMITGWV---IADPLLSLIFAAMILRGAVSVVKGSAHVLLQ
Pbe1            HVIGDLLGSVAAI-VSAIIILFTGWV---VVDVLVTLLVCGLIARSAYALVKETGVVLLE
Rxy1            HVLADLLGSLGVL-VSGMVVLLTGWY---PADPLVSAAIGLLILASSWRLLRDSVNILLE
Sav1            HILNDLFAFIGTA-IAGLVVVVTGFA---RADAIATLVVVVLMVKAGYGLLRDSGRIFLE
Asp1            HVLSDTVSSVAILGGAGAMALRPDLR---WLDPVLSLAIAALILWGALRLIFEITDILME
Mfe1            HVVGDALGSVAAV-IAGVVIYFTGWM---PIDPILSFLVAAILAWGGWRLIRETTAELME
Sce1            HVLSDALGSVGAI-TAGVLILAFGWT---RADPVISAAIGALVLWGGWRLVRDTSRVLME
Cbu1            HVVSDIVGTCAVL-LSGIVIFFSHWA---LIDPLLSVLIGILIIISSVRLWRESMTVLME
Asp3            HVLGDLLGSVAAL-ASGLVIQFTGWV---TIDPLLTMLICGLILASTLSLLKQVVHTLLE
Mfl1            HVLGDLLGSVAAI-LAGAVIYFTGWM---PIDPILSIFISVLILVSTLRLLGEVLHVLME
Cvi1            HVLGDLFGSAAAI-ASGVVIYLTGWM---AADPLLSMLVALMLLAAAWRLIRQAVMVLME
Psp1            HVMGDLLGSVAAL-IAGIVIQLTGWM---PIDAILSILVSLLILKSTISILHESYHFLME
Tca1            HVIGDFLQSFGVF--VAAAVIYFNE-QWVIIDPICTFIFSVLVMFTTFNIIKDTLTVLME
Aae2            HVLSDFVQSLGVF--IAALVIYFKP-EWNIVDPICTFLFSILVLITTLAIMKDALMVLME
Cel2            HVLGDLLQSLGVL--VAALFIYFQP-SWVIIDPICTLVFSVIVLCTTIYILRDAMIVLLE
Tca3            HVLGDLLQSLGVL--IASIIIKVFP-SAKVADPICTLIFSAVVIFTTAKVAKDSIWLLLE
Orf2            HVLGDLLQSFGVL--AASILIYFKP-QYKAADPISTFLFSICALGSTAPTLRDVLRILME
Bta1            HVIGDFLQSLGVL--VAAFILYFKP-EYKFIDPICTFLFSILVLGTTLTILRDVILVLME
Xla1            HVIGDLFQSISVL--ISALIIYFKP-EYKIADPICTFIFSIFVLITTVTVLRDLLNILME
Cel1            HVIGDLVQSIGVL--IAALIIRFT--GWTLADPICTFLFSIIVLFTTVTVMRDIFFVLME
Tth1            HILGDTIQSAGVI--IGALFVFFGGEDYFIADPIITIVFTIVVTFTTLPVMKDSIKVLME
Ddi1            HVLGDCFQSIGVM--VASCIIWVHP-HWKIADPITTLIFSVIVLGTTIKLLRESLGVLME
Ath2            HVLGDSIQSVGVM--IGGAIIWYKP-EWKILDLICTLVFSVIVLGTTIGMLRNILEVLME
Dsp1            HAAGDTLASVGVI-ISAVVVIYSGNY---LADIIVSVIIGLIISLGGINIFRQGLRVILE
Sau2            HFMGDLLNSIGVI-VAVVLIYFTGWR---IIDPIISIVISLIILRGGYKITRNAWLILME
Cje2            HMMSDLLGSLAVI-IGGIVVYFSGIV---YIDTILAIVLSILLLRWAIILLKQSANVLLE
Cfe1            HMMSDLLSSVAII-LGGIIVYFTNLW---WIDSALALFIAIIIAKWSFSLARDSMNILLE
Abu1            HMLTDALSSVAII-IGYIVIYFTSWY---FIDIILAVIVALVIAKWAIDILKSSTNTLLE
```

```
Kst1            HMLGDTISSVGVI-IGAVIIYYTNWY---IIDPIISIMLCILILIWSYKLIMESVDILLE
Lbo1            HVLSDLLSTAGVL-IGSILIYLTNWN---WLDPLISILISILILRSAWGIFQESISILLE
Pae1            HILSDLAGSVGVV-AGSILISLTRIT---TIDSIISFIIAILIVQSALKIIREAVDVLME
Aph1            HVIGDLLCSVAAI-ASSIIIRFSGWQ---IIDPMLSLLVSTVMLISAFSIVKNSSNILLE
Wen1            HVIGDILGSVAAI-LASIIIMLTGWQ---IVDPILSVFVSVIILNSGYKILKNSCHILLE
Mbo1            HVIGDLLSSLAVI-VAALWIALTGQT---VVDPLLGLAISVLILFSSFSILAESFRILLQ
Mth1            HVLGDAAASLGVI-AAALVIWRTGWY---PADPIISLLISVMIFAGSYRLIKDSFRLIME
Gbe2            HVIGDAAASVGVI-IGGIIMYFTNWY---LLDAVISIGIGFIIFWGSWRVMRESVHILLE
Chu1            HLMSDALVSLGLV-IGGIIMFYTQWF---WIDSVLSMIVAIIILFSTWKLLKDSLRLSLD
Lin1            HLLADGLVSLGVI-ISGLLIQWLGFF---WIDPVVGLLVGLVILYGNIPLFRQSLRLSLD
Rpa1            HMVADAGVSAAVV-VAGVVILFTGWN---WIDPAVSLAVAFVIIWGTWGLLRDSTAMSLD
Van1            HMAGDAAVSLGVI-ITAFIINITGWD---WLDSATSLAIVVVILIGTWGLLKDSINYSLD
Gox1            HMAADAVMAFSVV-IAGLLIAFTGYT---IIDPIMSLIVSVSIVIGTWSLLRSSLNLALD
Afa1            HMAADAAVSLGVV-ASGCLALWQSWV---KLDPVVSLVIAVIIIFATLSLFKRSLHMLFD
Bph1            HMLADAAVSAAVA-VSGLAIVFTGWT---WLDPVMSLVVVAVIVYGTWGLLRDSLSLALN
Lpn1            HLFYDALISVGVV-LSAGVLYLTGWV---WIDPVVGLLIALVILKGTWSLFADSFRLIID
Rgr1            HLTYDALIALGVV-LAGAVIYFTKWL---WIDPIVGLLIVGIITWGSWNLLRRSVELILG
Lme1            HLLADAGVSVGVI-IAGLLIQVTGWH---VIDPIVSMIIGVIIAVTSWPVMKSTFNLALN
Sde1            HLLEDVLGWVAVL-IVSVVLMFVEWP---ILDPILSILFTLFILVNVLKNLTQTLQIFLQ
Ilo1            HLLEDVLGWVAVL-IVGIVLLFVDWP---ILDPLLSIGFTLFILVNVLRNLWATLKLFIQ
Asp4            HLIEDVLGWVAVL-IAGTILYFVYIP---WLDPGLSLAISIFILWNVFKNLKETLYIFLQ
Mma1            HLMEDALGWVAVL-VGSIVMLFADVP---IIDPILSLLIAVYILYNAFKNLRAILNVFLQ
Pgi1            HLLEDVLGWAAVL-VVSIVMLFVNAP---ILDPILSLCITAWILYNVYGNLRETFKVLLQ
Bsp1            HMLEDILGWIGVL-IVSIVLLFKEIH---ILDPILAISIAGFVLFNAFKKTVKTMRIFLD
Efa1            HMLEDVLGWIGVL-VVSIVMNFTEAY---RLDPILSILIALYILYKTVPEFLSTMKILLN
Lla1            HALEDVLGWLGVL-IVSIVLHFVDWY---WLDPLLSILIALFILSKAIPKFWGTLRILLE
Sgo1            HFLEDTLGWLAVI-LMAIILRFTDWY---ILDPLLSLVISIFILTKALPRFWSALKIFLD
Mba1            HLLEDILGWVVLL-TGSIIIRFWNKP---VIDPIMTIGFTVFVLWGVSKNAKEIFNLLLE
Dsh1            HIIQTFVGSLLII-VTALVIEFTGFL---AIDPILGMAFGVVLLWASVGVIKEAVHILME
Ecu1            HVVGDLIQSVGVIIAGMVTYFYPSKA---IVDVICTMFFSVLVLISTGFVFRDGVYILAE


Sus1            GLPRGIHLVDVA--KAMGGVTGVLGVHDLHIWSLGSKTHALSCHVLIEDVPPSAS-----
Aba1            GTPRGMNVQRLA--ERMCSIDGVIDVHDLHVWSLGSETHALSCHIRIADLRASES-----
Mca1            STPRDVDMGSMV--RDMMQVEGVHGVHHLHVWSISRQLRMLSAHIVTDDIRLSEG-----
Tko1            AAP-DLDLDEIK--REIESIPGIRNAHHFHVWRIGEKEIHFECHVEV-NDMPIS------
Pfu1            ASP-NLDFNEIK--REIESIPGVKNAHHFHAWRVGEKEVHFECHVEV-NDMLIS------
Fba1            FTPEDIPVDEII--KEINAFESIKNVHHIHVWQLNEDETHFEAHVDFESNITLS------
Nsp2            FAPKSIDIEKIA--DEVKKIEKVENIHHIHIWRLGEHDIFLEAHIDFKENLPLQ------
Fno1            STPHNIDVDEII--KRLENISEIENVHHVHIWSLDGKTNFFEGHINLKEDVTVS------
Cac1            TTPVNLDMDDVK--EQLLKIKEIKGVHHFHIWTLDENNIVLEGHIEI-DDILVS------
Lhe1            SNP-NIDLNSVN--DIVLSFPEVKNIHHVHVWRYSDDFIMMDAHINVDRNLHAD------
Lre1            SSP-DLDYDSIE--NDLKQIDGVTAVHHVHAWMMDEHRIIFSAHLNCD-DLPLS------
Pmo1            GTPTDIEIEKIK--AKLEKFDFVKDVHHIHIWTTDGKDKYMEAHIRLQESLDQNNY----
Tme1            GIPKGVDLDKVK--KIIQNFSFVKDVHHIHVWSLDGIELYLEMHVTVDG------K----
Sac1            ATPPGISVKEIK--RAVEAIEGIREIHHIHVWTLSPDRAALAAHIIVDDQMLSQ------
Dol1            GTPRGLDLEKVR--ADMEAVDGVLSVHYLHAWAMGGCSVAMTGHVVVTDQMVSA------
Orf1            GVPAGVELAALS--TAVRAVDGVVGVHHLHVWQLDERAPYFEAHVVIDRVDADRL-----
Rba1            GAPANFHFDEMM--ADLGGMDGVSGIHHVHLWELDESHRALEAHVVIEPTRFEDL-----
Oin1            GSPPDMDAGAVV--EAMTKVAGVRDVHHVHLWQMQEHEVALDCHVVLEGQDWTDL-----
Eli1            AVPDEVEFDQMV--ENLRALGGVQDIHHVHVWNLGEHHRALEAHLTLVDYSQTAF-----
Sfr1            AVPVDIDIDKIC--KDIESMENVEKVRHIHVWQLDDSEVYLETSVNLK--TMEQF-----
Pde1            AAPAGVDHAALI--GAMAGVEGVAGAHHLHLWQIDERRTALSVHLVMR--DGADA-----
Pcr1            GTPEGISLVNVE--EKLLAHPQVQKVHDLHVWSITSGLNALSCHVVVDGEMSIHE-----
Bli1            GKPKDIDAEALK--NGLLSIPSVREVHDLHIWSISSDMPSLSCHIVAD-ENSDRD-----
Sau1            GTPSDVDIDEVI--TTITKDSRIQSVHDCHVWTISNDMNALSCHVVVDHTLTMKE-----
Sha1            GTPSDIDLEQVI--YTIMGHEEIQNVHDYHVWTISNDMNALSCHAVVDKSLTIEA-----
Ssa1            GTPIDINMSEVI--ATIKEEDAIQSVHDCHIWTISNELNALSCHAVVPHEMSVAQ-----
Bsp2            GTPSNVDIQEII--GLIEQTDGIESIHDLHIWTITSGTNALSCHAVVNEHLKIAD-----
Ccu1            GAPANVDTSEIL--AAIGGTAGVRSTHDLHVWSITSGVNALSAHVVIEGDMSVSA-----
Ame1            ------------------------------------------------------------
Gka1            GTPITIDQNEVK--KALESIEGVINVHDLHIWTITSGLDSLSCHILIEDHQD--S-----
Emi1            GAPGHINIEALK--SSVCVIKGVVDAHDMHVWSISSGFLVLTAHITVSEDAD--R-----
Aae1            VAPSHINTEELE--KELLNLQGVKGVHDLHVWSITPGTEVLTVHVVVEDTSI--------
Nsp1            YAPKSIDPVEVE--KLIKSFAGVVQVEKLHIWTISSNKVMLCANVIVECATNQER-----
Msu1            DG------------------------EEFSMDTHSH------------------------
Cdi1            RVPKTVDVETIR--SRIAQIDGVGGVHDVHVWSIDGQQDIATVHVVVDENVNVK------
Cje1            QAPPGYRPAEVD--ALLRQVDGVLDVHDLHLWSLDGTSALASVHLVVPEGR--D------
Tva1            SCPEYIDVELIK--ADLMKIEGIVAVHELHVWELCKEIYLALLHVVVDSKE--R------
Ath1            RVPRAHRQDLKEAMRNILKTKGVCSIQRLHVWSFTNSDVVATLHLLVSADSDK-------
Vvi1            RVPRVHEHDLKDALKNVMKIRGVCGIQNLHVWSFTNTDVVGTLHLHISTEADK-------
Clu1            RLPPEYEKELHVALEKIQKIEGLISYRDPHFWRHSASVVAGTIHIQVTSDVLE-------
Tca2            RQPYALDNVLPQCYQKVVSLPGVYSVQEPHFWTLCSEVYVGALKLEVSKNVDP-------
```

```
Cel3            DVP--EEEEFEYHINEILEIEHVESYSNAHMWQHKSDINVASVHVQVKEEANA-------
Hma1            RSPVSSEELR----DELTTLDGVDQIEDLHVWQVCSQLTVATVRLTDTSTTLEE------
Jsp1            GVPEHIDLEKLR-KSV-SDIAGVRSIHDLHVWSITSGKISLTMHVVGDLEAFNR------
Xor1            GVPKGIDLAQVQ-QAL-TSHPGVEDVHDLHVWALASSTPALTAHIVVN-EATDR------
Mpo1            GTPPGVDLAALK-TEI-EAMPGVRRAYDLHAWTLTSGFDAMSGHVVVD-DVAGG------
Dra1            GVPEGLDLDALR-AEL-RALPGVQDVHDLHVWSVTGGVVNLTAHLVSD-RAP--------
Asp2            ATPKGVEVQMIR-EHI-LSVHGVVSVHDIHIWTITSGVPVFSAHVVVEDAVLSARGA---
Tfu1            ATPRGLDLSEVR-RHL-QTHPNVVDVHDLHAWTITSGMPVLSAHVVVEDSALADSG----
Kra1            ATPRGLDLDAVR-RHL-LELPHVRDVHDLHASQITSGLPVLSAHVVVDDGCFHDGHT---
Ace1            AAPESIDLAEVR-AHL-IDVPHVVDVHDLHVWTVTSGLPTLSAHVVLEDHCFASGHA---
Aod1            QAPANVDVTELR-AHM-LDVDGVLDVHDLHVAAVSSHLVTVTAHVTVSEHADGP-SR---
Bad1            ETPQELDLDEVR-EHL-EQVPHVVAVHDLHASTVSTGMPVVMAHVVVERGLTMD-DA---
Str1            AAPEHLQVTAVQ-DRL-IAVPGVTDVHDLHVWTLTSGIEVTSAHLTVDPGADIG------
Rer1            HAPAGVSITEVT-QAL-QGIAGVKDAHDLHIWTLTSGMEVASAHLTIDDESDPT------
Nsp3            HVPDGMQVDQVV-ADL-EALPGIEDVHDLHVWTLTSGMNVATAHLVVAPDADPQ------
Rsp1            ASPEDVDVESVQ-ADL-EALPGVTGVHDLHVWTLTTGMDVATVHLTTD--ADSS------
Csa1            GTPEGIDVDGIR-AAL-EDIEGVVSVHDLHVWGLTPQDPLLSLH--LVVRDDMSH-----
Oal1            AAPDHIDPSVLR-ADLTAEIEDVLDIHHVHVWSLTPDRPMATLH--ARIEPEADA-----
Mlo1            GVPPSLDRDLIA-TDLETTIQGVREVHHMHVWSIDGSSNMATLH--ACLDEGVDA-----
Lag1            GAPADIDRDTLK-TDLLNAVPGLVDVYHIHLWSLAEGKINATMH--ATLKPEDDA-----
Oan1            GAPDNATPEKIE-RHLVENIADVASVSHIHVWSITSGRTLATLH--VRPKADVDA-----
Pat1            GTPSQLSVEALQ-KDVTLNIPEVRNIHHVHLWQVGE-KPMMTLH--AQVVPPHDH-----
Neu1            VVPRGMDTRVVE-NGL-TQLPEIEEAGHFHAWILADETTIATVH--VSPARGVDP-----
Gbe1            GTPADIDLDAIA-SDLVHTIPGVQRVHHLHAWSLTGNDRLITLH--AVTGAGEETGE---
Pbe1            GAPQEIDPTDLR-DVIRRDVRGVTDVHNVRLWMLTPGANQATMH--VRVRDPYQG-----
Rxy1            AAPHGMDAAEIG-RRM-ASTSGVAEVHDLHVWTITSGFPALSAH--VLVAAGEDC-----
Sav1            AAPADVDPDALG-DKL-VAQAAVVEVHDLHVWQITSGQAALSAH--VLVQPGSDC-----
Asp1            GVPRHLDVAAVT-RQM-ESAHGVSAVHDLHIWTISSGMYALSAHLVVHAESIGRN-----
Mfe1            AVPDAIDTEVVE-ACL-KAVQGISDIHHIHLWKLPDGRMGMSAH--VEIESMSEW-----
Sce1            GSPIEIDLAHVE-DTI-RSVPGVVDLHDLHVWSISDGFNVLTVH--VVLAKGHHG-----
Cbu1            GVPAHLNIKQVS-QTM-VRFEGVKAVHDVHIWTLSSGVTALSAH--VNIKNITSW-----
Asp3            GVPDHLSLPEVG-QAM-AEVEGVRSVHDLHIWSLDSRQAALSAH--VVLGDSSTW-----
Mfl1            GVPLHLDIQAVE-HAM-SASDKVQGVHHLHIWSLSSEKTALSAH--IVLENILDW-----
Cvi1            GVPPHLDYNRIG-EAL-SAIAGVRSVHDLHVWTMSAERVALSAH--VRIAAPQDW-----
Psp1            GVPLHIDYLAVG-RDL-KAVPGVLAVHDLHVWEMTPSFPALIGH--IEISDMQEW-----
Tca1            GAPKGIDFNQVM--KTLLNIEGVKRVHNLRIWGLSLDKIAMSAHVAIITGQSSWLRDVGR
Aae2            GTPKYLDFTEVM--QTFLQIQGVVRVHNLRIWALSINKIALSAHLAVEPNTN--------
Cel2            GRPSNIDFAKVF--SSLEDIEGVKKVHDLRIWSLTMDKIALSVHLEIDANSQ--------
Tca3            GSPK--HSGDLA--FELLNISNVRHLHNLHIWALSPGKDAVSVHLCVG------------
Orf2            GTPRNVGFEPVR--DTLLSVPGVRATHELHLWALTLTYHVASAHLAIDSTAD--------
Bta1            GTPKGVDFTAVQ--NLLLSVEGVEALHSLHIWALTVAQPILSVHIAIAENAD--------
Xla1            GTPRGIHYSDVK--QSILAVDGVKSVHSLHLWALTMNQVILSAHIATDILGE--------
Cel1            ATPSHYDLSDVK--KALSALEGVKGVHDLHLWSIGMDKTAFSVHLALESPNR--------
Tth1            GQPDDIKYETVK--EKLEKVSGVVNVHDLHVWSINPGFVSLTAHICSKTPSVT-------
Ddi1            GVPPEIDVSEVK--GDLSEIEGVTEVHDLHIWSITLGRPALSVHLTILPTID--------
Ath2            STPREIDPTMLE--KGVCEIEEVVAVHELHIWAITVGKLLLACHVKIRPEAE--------
Dsp1            ASPKDINISQLA-ASVKN-IPGVKDFHHIHIWSISPEIHALSAHVLIDDCP---------
Sau2            SVPQHLDTDQIM-ADIKN-IDGILDVHEFHLWSITTEHYSLSAHVVLDKKY---------
Cje2            SSP--VDIEKVR-QVLLL-NPSVDEVVDLHITQITNKMLVASMHLKVRVCN---------
Cfe1            ASP--IDINAAK-NVMLE-NPLVLDVHDLHISEITHKMYVLTAHIVLNKDN---------
Abu1            SSP--VDVKEVQ-EYIEK-NEKVLELHDVHIWEITQDMYNMTAHVKIDKKY---------
Kst1            ATPKDINVGEVI-SQLEQ-ITGVDGAHDIHIWTITSGMYAMSVHIDTKDMP---------
Lbo1            SSPQTFEILHIL-EHIRE-IEGIRQILDYHFWAITRGVYACTLKVSVVD-----------
Pae1            SVPPELDIQEIE-EALLN-FRHVEGLHDLHVWALTSGVNALSCHLLVKN-----------
Aph1            GKPYNIDVGELQ-RNITSAIPNVIDVHHVHLWSLTAEYPIMTMHVRIACGSSD-------
Wen1            GTPEGISAEEIK-SKITSELPEVIDVHHIHTWSLSDNYFIITMHAKIKQN----------
Mbo1            FAPRDVPIEDVI-AAMES-VPGVSGVHNVHLWTLCSNINVLDAHVYCCEND---------
Mth1            GVPECIRTDEVV-DQIST-ASGVIQVHDLHIWGVCSSNILLSAHVVVSP-----------
Gbe2            GVPRGVEVEQVS-AAILE-VEGVEEVHHVNIWTICSHILALSGHIVVPPTF---------
Chu1            GVPEYIHIHEIK--ETALKIAGVKDLHHIHAWAISTTENALTAHLV---LDKNTTIAD--
Lin1            STPETIQSGIVE--KELKSIEGVLNIHHIHIWSLSTTENALTAHLV---LKNDLTFEH--
Rpa1            AVPPGIDPVAVR--AFLESRPGVAQVHDLHIWSMSTTEVALTSHLV---MPGGSPG----
Van1            AVPSNVDMVGLK--AYLAALPNVATFHDLHVWPMSTTENAMTVHLV---VDEYDKG----
Gox1            AVPAGIDPDAVQ--AALLSLDGVSGLHHLHIWAMSTTETALTVHLV---CDPTKPVST--
Afa1            GVPDDIDLNAVR--SVLLEIPGLKSLHDLHIWNLSTTETALTVHLV---LDENIDV----
Bph1            GVPRGVDMQKIR--AYLAAQPGVTDVHDLHVWALSTTGNALSAHLV---IPGGHPG----
Lpn1            GVPRDISWIKVS--DFLLAIPGVKSIHDLHIWAISTKENALSVHLF---MPEESLS----
Rgr1            AVPYGVDSKEIY--TYLKNLPGVQEVHDLHIWGLSTQETALTAHLI---MPETNLS----
Lme1            GVPDNVNESDVL--RYLSTHEGVEQLHDLHIWPLSTTEAAMTVHLS---VKKNVDE----
Sde1            AVPDTQQQQQIQ--QALVTLDAVDSLHHLHFWSLDGEQHVLTAHIV--LAHELTPTAQ--
Ilo1            ATPDKETYRQVA--DTLLELPHVADLHHLHFWSLDGEEHVLTVHLV--LSKNLDIEAR--
Asp4            AVPAELSLEEIK--KKICDIDQVESVHHTHLWSLEGAHHVFTTHVR--IKKVDNLKEV--
Mma1            AVPPKFDLETTK--EKILKIPHVIDLHDVRVWSMDGSEMIMSLHLV-VEENLSSAQCK--
```

```
Pgi1            GVPDGIDLEGLK--TDILSLPHIRSVHDIHLWTLDGESHVMTIHVVYCPDDFSSPQAV--
Bsp1            GVPEGINLDAVE--KDMKKVEGVEEISHLHIGSIDGEKNAMSAHVK--VRNGLEARDE--
Efa1            GSPENVNVENLA--SSINNIPAVKALSHFHIWSLDGEENALIVTVL--IDS-SDINKA--
Lla1            SVPEDIDYKKLL--IALEKLPEVRSVTQLIIWSIDGEQNAAMIHIV--IPE---NQDF--
Sgo1            AVPDGVETGDLA--KDLEALTNVKSVNQLSIWSMDGLENNALVHIC--IKD---WEQM--
Mba1            GVPEYIDIDEIK--KSILSVEGVKMVHDLHIWSLEGETVLLTAHVV---VEDKYLQTP--
Dsh1            GTPEGTDLDAVT--ADLNGQDGVLDVHHVHAWTLTSGKHAFSAHIRHQSPAEAAD-----
Ecu1            GAPTDLDIDGMR--SDIQDVENVYKIVDLYAWSISMNRSAVSIRILADDLLISDY-----


Sus1            ------------------------------DCILRSLN------------TLLEHKYGIS
Aba1            ------------------------------EAILKAVN------------EAVAHDFHIH
Mca1            ------------------------------EGLQKRVT------------RLLAQRYGIA
Tko1            -----------------------------EAQRLIDE-----------IEERLKR-FGIT
Pfu1            -----------------------------EAQQIIDV-----------IAERLRR-FGIT
Fba1            -----------------------------EFDIILHQ-----------IEELLFHKFEIN
Nsp2            -----------------------------EVTQIIAD-----------IEKLLREKFYIS
Fno1            -----------------------------QSMVIYKK-----------IEQELSK-MEIY
Cac1            -----------------------------ETRAISDK-----------IEHILNEDFHIT
Lhe1            -----------------------------ELENLYQD-----------IGKKLKSELGIN
Lre1            -----------------------------QVERIYSQ-----------VEKILHEKYGIC
Pmo1            -----------------------------DLDNCIDK-----------LNKVLKEDFEID
Tme1            -----------------------------DYDDYLRR-----------IKETLKEH-GFR
Sac1            ------------------------------VDLLVSR-----------VRELLWSRFGID
Dol1            ------------------------------TEQLART-----------LGDRLLDGFGID
Orf1            -------------------------------EDIKRA-----------VKWALRERFGIA
Rba1            -------------------------------ETIKRE-----------IKLYLTSEHNIR
Oin1            -------------------------------EAVKQA-----------VKKMLANDFGIT
Eli1            -------------------------------EGVKRR-----------ARAMLEKDFGIA
Sfr1            -------------------------------QDLKP------------IKDLLAAQYGIH
Pde1            -------------------------------VMAVRQ-----------AKAMLRRDFGID
Pcr1            ------------------------------SSILIGS------------LERSLLELGIH
Bli1            ------------------------------R-ILKQV------------SKYLRKECNVE
Sau1            ------------------------------CELLLEN------------IEHDLLHLNIH
Sha1            ------------------------------CEQLLKQ------------IEHELAHLNIQ
Ssa1            ------------------------------GEQLLNK------------IEHKLQHLNIQ
Bsp2            ------------------------------GEHILRK------------IEHNLEHKGIK
Ccu1            ------------------------------AQEVTHE------------IERKLAHLGIA
Ame1            ------------------------------------------------------------
Gka1            ------------------------------QKVLQEA------------IHFIEDHFKIQ
Emi1            ------------------------------DLVLEEA------------RKIIADNASIE
Aae1            ------------------------------CNDILKE------------VEKIAHKYGIK
Nsp1            ------------------------------DRLINKL------------QIHLQENFKIA
Msu1            ------------------------------------------------------------
Cdi1            ------------------------------DCTTLDR------------IQKVFHDAGID
Cje1            ------------------------------PAAVLCA------------AQAALQERGIA
Tva1            ------------------------------NKKVLEQ------------CHNIMISHGVY
Ath1            -------------------------------TDTKLQ------------VSRLLEDAGVK
Vvi1            -------------------------------ASTKVQ------------VSNILHDAGIK
Clu1            -------------------------------QRIVQQ------------VTGILKDAGVN
Tca2            -------------------------------KYVVSH------------TQMIFASVGVR
Cel3            -------------------------------QMIRHR------------VSNILKSTGAT
Hma1            ------------------------------QRRIQSR------------VHDYLTNRGID
Jsp1            ----------------------------DRLLTEIQT------------TLAEQWE--IH
Xor1            ----------------------------DRLRDALAT------------LLHDRFD--IV
Mpo1            ----------------------------PAVIRAVRG------------LMKERHG--IE
Dra1            ----------------------------AELLPAVHE------------VAHG-AG--IE
Asp2            ----------------------------DQVLDKLAT------------CLGSHFD--TE
Tfu1            -----------------------------RLLARLHD------------CLSTHFS--ID
Kra1            ----------------------------PQMLDALQA------------CVAEHFEVAIE
Ace1            ----------------------------PQLLDALQN------------CLAGHFDVT--
Aod1            ----------------------------DRIVHQLGE------------CACHHFP--IA
Bad1            ----------------------------ADVLSQLQN------------CLREHFPVSVP
Str1            -----------------------------DVLGSAQT------------ALREEFR--IE
Rer1            -----------------------------KVLAVAQD------------LLAKQFD--LV
Nsp3            -----------------------------RVLTDAQA------------LLSSSHR--IE
Rsp1            -----------------------------VVLESART------------VLES-HL--LS
Csa1            ------------------------------AVMLKAA------------YARLHERFGIS
Oal1            ------------------------------PAIIVAI------------KSRLAERYKLT
Mlo1            ------------------------------HRAVSAI------------KKRLASEHGIS
Lag1            ------------------------------ENVLRQI------------RERLKSRHGIG
Oan1            ------------------------------RSVMRAV------------ERELKSQFKIE
Pat1            ------------------------------DALLRRI------------QEYLLKHYQIE
```

```
Neu1          ------------------------------LSLPPLV------------SSWLRERYAID
Gbe1          ----------------------------DRDRILDAI------------QHRLSDRFGIS
Pbe1          ------------------------------DRILEDI------------KSLLAAR-GIL
Rxy1          ------------------------------HAKRREL------------EQLLREEYGIS
Sav1          ------------------------------HAVRRDL------------EELLRQDYGIT
Asp1          ------------------------------DDILNEV------------KHGLRRSFGID
Mfe1          ------------------------------DEILPLL------------LEKLRK-QGVD
Sce1          ------------------------------TNVAAAV------------ARRLREKHALT
Cbu1          ------------------------------DDVLLGL------------KSTLKQKYDIN
Asp3          ------------------------------PAVLARQ------------QRLLSERFGIG
Mfl1          ------------------------------HEVLNNL------------RHMLHERFNIE
Cvi1          ------------------------------PRILAAC------------QLMLSREFCID
Psp1          ------------------------------PSIMAKI------------NAMLLDKHGID
Tca1          LDHQALADWLAGHTSGSGKFRGFNAPRPGPTPALLDPNEAKMERWSSVASNDRQPNYLCL
Aae2          ------------------------------TETILQQ-----------ATRTVHAKYDFF
Cel2          ------------------------------SQSILRE-----------TRKMLKQTYNVH
Tca3          -----------------------------------------------------EFHYLFL
Orf2          ------------------------------PEAVLAK-----------ASSRLYSRFGFS
Bta1          ------------------------------AQAVLKA-----------VSDRLQEMFHFH
Xla1          ------------------------------SKRILKD-----------VTQNVCSSFPFH
Cel1          ------------------------------AMENVAE-----------ARSLIRRRFGVA
Tth1          ----------------------------------------------LFEATKLCKKLGIV
Ddi1          ------------------------------PEEILSI-----------ANKILLEDYEIN
Ath2          ------------------------------ADMVLDK-----------IIDYIKREHNIS
Dsp1          --------------------------VSQADYIRQQI------------ETSLREGFALN
Sau2          --------------------------EGDDYQAIDQV------------SSLLKEKYGIA
Cje2          --------------------------LKEFEKLSQDL------------SHKLLHEFEIG
Cfe1          --------------------------IFKFDEIINDL------------SLSLKNRLEIG
Abu1          --------------------------LDDYEEILHKI------------NHNLKEKFKIV
Kst1          --------------------------ISKTAAISREI------------NRVLVEKFRIG
Lbo1          --------------------------LKHTEGVIFNS------------NRILKSKFGID
Pae1          --------------------------LDAGQNILVPI------------HRELKEKYNID
Aph1          --------------------------VASSVQVVKSI------------KKLLQERFSIS
Wen1          ---------------------------VQHTNVLYEI------------KKILLDKFEIA
Mbo1          ---------------------------PETREQIKEE------------IKHRLEHFRIG
Mth1          --------------------------ESDHQRTLSEI------------RDKLSSIFGIE
Gbe2          --------------------------KGEHGPILRRI------------EERLFERFHIS
Chu1          ------------------------------ENIIKREL------------KHELEHKNIH
Lin1          ------------------------------QRIIKSKA------------REILKGLKIE
Rpa1          ------------------------------DAFLIEIA------------HELQHDFGIA
Van1          ------------------------------QGMPDEIK------------HYVHDHFEIE
Gox1          ------------------------------DLVIARAA------------ELVRTRFDIA
Afa1          ------------------------------DAALRGAT------------NILIERFSIH
Bph1          ------------------------------DRVIDAIV------------GTLRTEFDMH
Lpn1          ------------------------------DDLRRELV------------EKLKHDFSIQ
Rgr1          ------------------------------DEEYFKIN------------QVLAEKFHIQ
Lme1          ------------------------------QELLANIS------------SDLREKYDIS
Sde1          ------------------------------LNLKQQIA------------VTLAPYKLS-
Ilo1          ------------------------------SDLKQRID------------DVLAPYALS-
Asp4          ------------------------------LDAKTAIK------------EVLKAYPFS-
Mma1          -------------------------------ALKHEVK------------HTCQHLHIG-
Pgi1          ------------------------------FDMKESVR------------EKCSAHGIR-
Bsp1          ------------------------------EKIKETIR------------SQLSSCNLT-
Efa1          ------------------------------REIKEEIA------------ELAHTSGVKN
Lla1          ------------------------------SEAKIAVR------------TLLESEKVC-
Sgo1          ------------------------------METKEAVR------------QLLEERGVQ-
Mba1          ------------------------------DKMRQSIK------------SKLEKHHIE-
Dsh1          --------------------------------LLNRAY------------RRLTEQHGFH
Ecu1          ------------------------------ENILLEVN------------HIIKGKYLVD


Sus1          HTTVQFEHVSCAISETGCAIPVSEQHHH-HDH----------------------------
Aba1          HTTIQFEHEVCEVAH-GCVIPVSHAHDHTHDHA---------------------------
Mca1          HTTLQLESESCQPDSLYCDISRPNHRYHGE------------------------------
Tko1          HVTVQLEAG----------RCEDKNTICGEKGD---------------------------
Pfu1          HVTVQLEAG----------RCKEKGMICDEGV----------------------------
Fba1          HINIQPEFG----------KCDSKDVIVQD------------------------------
Nsp2          HVTLQPEFQ----------RDDDKSIVVQRDPGA--------------------------
Fno1          HSTIQFEYK----------GCPECGVISHKK-----------------------------
Cac1          HVVIQFEST----------SCEDN--ICKI------------------------------
Lhe1          HVTLQAECERGRN--------DKMIVPCRGTNED--------------------------
Lre1          HVTIQAEYHRGKDEELFNTPVDEKNVISNDFKQD--------------------------
Pmo1          HTTLQFEKN----------RCLEGEKVT-------------------------------
```

```
Tme1          HSTIQLEQI----------NCGENCIAEIQ------------------------------
Sac1          HPTLQFETR----------SEDNTFLLCCPKEHNHHH-----------------------
Dol1          HPVFQFETR----------VCGQGELLCQMTSCEQEH-----------------------
Orf1          HSTLEIEFVGTH---------SDCIETAAVVAH---------------------------
Rba1          HSTLEFEFAGT----------SDCHDSDGNLIHDGCS-----------------------
Oin1          HSSLEFETSANAH-------QDAALFGHESGPTRAGKDAEHSVEASHDR-----------
Eli1          H--VTFEPCLATD-------CDDALIPAHPAPN-NGKNTVGD------------------
Sfr1          HSTIEVVVGNTLQ-------MENCFELE--------------------------------
Pde1          HATIEAEREGDA--------CSDAGDGHDGHRH---------------------------
Pcr1          HATIQVESAAHPQTSTHSEALVCNISE---RPAENNHIGHNH------------------
Bli1          HVTVQIE---GERCSPHES---CSLG--------ANHPRS--------------------
Sau1          HMSIQLET----PNHKHDESIICSGTHSHSHNHHAHHHAHVH------------------
Sha1          HMTIQLET----PKHQHDESILCSG--------FMHQHSH--------------------
Ssa1          HMTIQLET----KDHQHDDETLCSS----IYKTTAHNHAHDH------------------
Bsp2          HVTIQLET----ASHPHESSILCQLK-------NVHEHHHE-------------------
Ccu1          HVTIQIET----GAHGHADELICEIK-----GGEAGHLGHSH------------------
Ame1          ------------------------------------------------------------
Gka1          HATIQIET-----SQIHHGEMKV-------------------------------------
Emi1          HVTIQIES----CEHKNSCNGRCN------------------------------------
Aae1          HTTVQLEK---EGYACAECCPLLSPQGLKFHHHHHHGHEHEH------------------
Nsp1          ETTLQLTSPPKSAKLPLHPLFNQDLSLMLAANKSN-------------------------
Msu1          ------------------------------------------------------------
Cdi1          HVTVQLEHDTHMSHELPCQH----------------------------------------
Cje1          HATIQVERASHADHEGPQNVC---------------------------------------
Tva1          STTVQIEFVDDFPQGTDSVGSCFYATSFGSDKRIFQTPPVYQHSIGCPHVNLPGHEHDHD
Ath1          DWTLQVESVNS-------------------------------------------------
Vvi1          DLTVQVEYVKST------------------------------------------------
Clu1          NLTIQVEKEAYFQHMSGLSTGFHDVLAMTKQMESMKYYKDGTYIM---------------
Tca2          QIYVQLDYTPM-------------------------------------------------
Cel3          HSTVQVEKKTFAHRIQQVCPGYKAGYTVVRGSVIREKKHDHSHHHGHSHDDHSGHHH---
Hma1          HATVELVGRTDPDTDPVGTTNHSH------------------------------------
Jsp1          HTTIQMEA--------------------EACDQATNDHTFGPPGDSEKDHPPEKSS----
Xor1          HVTLQVESG---------------DCGTEPCGTPKPAADAGHDAHHGHSHAHGHSHH---
Mpo1          HVTVQVED-------------------------------EALSAEFPHLPV---------
Dra1          HVTVQVEP-------------------------------PGLHATDAALHP---------
Asp2          HCTFQLEP-------------------------------ESHSEHESHQHA---------
Tfu1          HSTIQVEP-------------------------------VGHAHHENACRA---------
Kra1          HSTFQLEP-------------------------------AAHAAHEAGAHH---------
Ace1          HSTFQLEP-------------------------------RTHLTHEVGAHR---------
Aod1          HSTFQLEC-------------------------------AQHASHEHIEH----------
Bad1          HTTFQLEP-------------------------------EGYDSDSRKELHF--------
Str1          HATLQVEP-------------------------------DASIEDCGS-FKW--------
Rer1          HATLQVEP-------------------------------AAGDGRCQE-LPW--------
Nsp3          HATLQTET-------------------------------AP-TRQCHE-VTW--------
Rsp1          HATVQVES-------------------------------TANGRHCAENVTW--------
Csa1          HATLQVEGE-----ACLTGGDCQVTST-------------THP-----------------
Oal1          HVTVEVETG-----PCAD----QKT-----------------------------------
Mlo1          HATVEPEFG-----QCADEAGDHEHEH-------------EHEHDAAAHHSHHH------
Lag1          HVTIEITEG-----S---------------------------------------------
Oan1          HPTIAIDWN-DDADACSLSQASALTGH-------------AHGDRHDHDGHHHNHGPEHV
Pat1          HATIQMEYQRCDDDHCSFHQENHHLAI-------------HDGEKHDAEGHHHKH-----
Neu1          HVTVQIDPP-------------GQLEH-------------EHR-----------------
Gbe1          HATVQMETG-----LRSDDHPCRLSPL-------------PGQVHHADHDHNHD------
Pbe1          DSTIQIEMS-----PPSVWSPSTISAR-------------PAEAQCPDEAVRAREGTDLG
Rxy1          HTTLQVDHAGDH----AAHGPRFLP--------------LRPKRKTRSPHT---------
Sav1          HTTLQVDHIPEQLLQVALRGDRIPPDGAPHC------EDAHGPVHREEPHGH--------
Asp1          HTTLQIES------------VEYEH---------------LHDVHSH-------------
Mfe1          HATLQPESS--------HLQGDCSP---------------CNDHHC--------------
Sce1          HCTIQPEPLQEELLVTLRRPGAGAP---------------GGDGPAGNRSGA--------
Cbu1          HITLQPEA--------DIEEC-------------------TPCYKL--------------
Asp3          HVTLQPELPATVPLVFSPRNS-------------------TPPSDRP-------------
Mfl1          HVTLQPEIGNLHSDASANCWL-------------------TSTNHRHDHDDASDHLHGQP
Cvi1          HVTLQAEWPASAPAGKPVPID-------------------IISEDKLP------------
Psp1          HVTLQPEEVGQDEHDHEHDDHGLNTEEKPTTFLHQGDTFYVQCSSGESEHRMAYHVWGDP
Tca1          YDFHKFVEFGIPVQFDSQSTNLLREQLKKSGT----------------------------
Aae2          ETTLQIEEFQADMEDCNQCTNPV-------------------------------------
Cel2          EITIQIEEFGANRSDCGKCDFPTK------------------------------------
Tca3          FQ----------------------------------------------------------
Orf2          SCTLQVEQYQPEMAQCLRCQEPPQA-----------------------------------
Bta1          TMTIQIEDYSEDMKDCQSCRGPSD------------------------------------
Xla1          SVTIQVEPVEEQSPECMFCYEPTQ------------------------------------
Cel1          VATVQVEPFDEKIDSCDTCQQQETA-----------------------------------
Tth1          HSTIQVENFFEQDKYDYKKCEELH------------------------------------
```

```
Ddi1            HTTIQIEKPLVKDKCKDHSCPPPKPKKKKIKNDNLSSPPNQ-------------------
Ath2            HVTIQIERQ---------------------------------------------------
Dsp1            HITLQMECRVCRANDILCQLEIGRQSHSHKHH----------------------------
Sau2            HSTLQIEN---------LQLNPLDEPYFDKLT----------------------------
Cje2            HITIQPIRSENEI-----------------------------------------------
Cfe1            HITIQPEWRES-------------------------------------------------
Abu1            HTTFQFEW----------------------------------------------------
Kst1            HTVIQYGCECNNNNGESSKHLYNKEHAHEQDHTHAHDNK---------------------
Lbo1            YITIQCETPALIEKIQGLPLADSRGTQEHGHGHFHPH-----------------------
Pae1            HVTIQLE--------------DERFIAAHNKG----------------------------
Aph1            HVTIEIEHEECSDAN---------------------------------------------
Wen1            HSTVEIEYDECADNKILKH-----------------------------------------
Mbo1            HSTLEFECRECSDCRVLRELHDEPGEGHRHDG----------------------------
Mth1            HATIQLENEYCG--QGRVVFQKRVD-----------------------------------
Gbe2            HTTLQLETTRCTDTEGAKQFRHRPRAASLAHAHAHPHHHPHGTCQGGHHHHDHTP-----
Chu1            HVTLETERENDPCGAEVC------------------------------------------
Lin1            HVTLETEEERENCEQIDCN-----------------------------------------
Rpa1            HTTVQIETDP-NTVCALAPDHVV-------------------------------------
Van1            HVTVQIESS--SYACDSKHQHCGIQQ----------------------------------
Gox1            HPTFQLETQ--PSVCDTHQPCC--------------------------------------
Afa1            HSTIQCESPRFATDCTLNVRH---------------------------------------
Bph1            HATLQVDMGTTQHRCSLDHTPHVH------------------------------------
Lpn1            HVTIQVEKTEAECNDACHNPRIII------------------------------------
Rgr1            HVTLQVEQGQKDF--PCSQSLIC-------------------------------------
Lme1            HVTIQIETNEFDQSCNVI------------------------------------------
Sde1            HTNIELEMPDETCRDGDTSESRGSVGTHDGASIRD-------------------------
Ilo1            HTTVELEDPDEACRDN--------------------------------------------
Asp4            HYTVEVELEEELCELTEPHQH---------------------------------------
Mma1            HVTLELETLSEGCKLVK-------------------------------------------
Pgi1            HATIELDPEGCSCGMESC------------------------------------------
Bsp1            HSTIEINHS---------------------------------------------------
Efa1            HITIEITTSKDELEKGYAYGS---------------------------------------
Lla1            RSAIELDETVDEHKKHMIYEV---------------------------------------
Sgo1            NITIEVDSSQSNHAQHKRRVTDLEQHHGHHH-----------------------------
Mba1            HSTLELESEGFCSATECIFESKN-------------------------------------
Dsh1            MVTLQLETECLDERHARDLDIVQIAAAKAAQEKTDVRDAHPHPSPNTEGP----------
Ecu1            IVVVQIDTPNTFYGDRGFVVDGVAMDLKMLETPGMLIPQA--------------------


Sus1            ------------------------------------------------------------
Aba1            ------------------------------------------------------------
Mca1            ------------------------------------------------------------
Tko1            ------------------------------------------------------------
Pfu1            ------------------------------------------------------------
Fba1            ------------------------------------------------------------
Nsp2            ------------------------------------------------------------
Fno1            ------------------------------------------------------------
Cac1            ------------------------------------------------------------
Lhe1            ------------------------------------------------------------
Lre1            ------------------------------------------------------------
Pmo1            ------------------------------------------------------------
Tme1            ------------------------------------------------------------
Sac1            ------------------------------------------------------------
Dol1            ------------------------------------------------------------
Orf1            ------------------------------------------------------------
Rba1            ------------------------------------------------------------
Oin1            ------------------------------------------------------------
Eli1            ------------------------------------------------------------
Sfr1            ------------------------------------------------------------
Pde1            ------------------------------------------------------------
Pcr1            ------------------------------------------------------------
Bli1            ------------------------------------------------------------
Sau1            ------------------------------------------------------------
Sha1            ------------------------------------------------------------
Ssa1            ------------------------------------------------------------
Bsp2            ------------------------------------------------------------
Ccu1            ------------------------------------------------------------
Ame1            ------------------------------------------------------------
Gka1            ------------------------------------------------------------
Emi1            ------------------------------------------------------------
Aae1            ------------------------------------------------------------
Nsp1            ------------------------------------------------------------
Msu1            ------------------------------------------------------------
```

```
Cdi1          ------------------------------------------------------------
Cje1          ------------------------------------------------------------
Tva1          HDDHDHAHLHSHSHDHLHNEMQGNLRTPLNE-----------------------------
Ath1          ------------------------------------------------------------
Vvi1          ------------------------------------------------------------
Clu1          ------------------------------------------------------------
Tca2          ------------------------------------------------------------
Cel3          ------------------------------------------------------------
Hma1          ------------------------------------------------------------
Jsp1          ------------------------------------------------------------
Xor1          ------------------------------------------------------------
Mpo1          ------------------------------------------------------------
Dra1          ------------------------------------------------------------
Asp2          ------------------------------------------------------------
Tfu1          ------------------------------------------------------------
Kra1          ------------------------------------------------------------
Ace1          ------------------------------------------------------------
Aod1          ------------------------------------------------------------
Bad1          ------------------------------------------------------------
Str1          ------------------------------------------------------------
Rer1          ------------------------------------------------------------
Nsp3          ------------------------------------------------------------
Rsp1          ------------------------------------------------------------
Csa1          ------------------------------------------------------------
Oal1          ------------------------------------------------------------
Mlo1          ------------------------------------------------------------
Lag1          ------------------------------------------------------------
Oan1          HSKQEHER----------------------------------------------------
Pat1          ------------------------------------------------------------
Neu1          ------------------------------------------------------------
Gbe1          ------------------------------------------------------------
Pbe1          GIAPIPS-----------------------------------------------------
Rxy1          ------------------------------------------------------------
Sav1          ------------------------------------------------------------
Asp1          ------------------------------------------------------------
Mfe1          ------------------------------------------------------------
Sce1          ------------------------------------------------------------
Cbu1          ------------------------------------------------------------
Asp3          ------------------------------------------------------------
Mfl1          GHAH--------------------------------------------------------
Cvi1          ------------------------------------------------------------
Psp1          TNPKVLLCVHGLTRRGSDFKTLAQAMCKDYYVVCPDIVGRGESDRLSNPMLYAVPQYVAN
Tca1          ------------------------------------------------------------
Aae2          ------------------------------------------------------------
Cel2          ------------------------------------------------------------
Tca3          ------------------------------------------------------------
Orf2          ------------------------------------------------------------
Bta1          ------------------------------------------------------------
Xla1          ------------------------------------------------------------
Cel1          ------------------------------------------------------------
Tth1          ------------------------------------------------------------
Ddi1          ------------------------------------------------------------
Ath2          ------------------------------------------------------------
Dsp1          ------------------------------------------------------------
Sau2          ------------------------------------------------------------
Cje2          ------------------------------------------------------------
Cfe1          ------------------------------------------------------------
Abu1          ------------------------------------------------------------
Kst1          ------------------------------------------------------------
Lbo1          ------------------------------------------------------------
Pae1          ------------------------------------------------------------
Aph1          ------------------------------------------------------------
Wen1          ------------------------------------------------------------
Mbo1          ------------------------------------------------------------
Mth1          ------------------------------------------------------------
Gbe2          ------------------------------------------------------------
Chu1          ------------------------------------------------------------
Lin1          ------------------------------------------------------------
Rpa1          ------------------------------------------------------------
Van1          ------------------------------------------------------------
Gox1          ------------------------------------------------------------
Afa1          ------------------------------------------------------------
Bph1          ------------------------------------------------------------
```

```
Lpn1          ------------------------------------------------------------
Rgr1          ------------------------------------------------------------
Lme1          ------------------------------------------------------------
Sde1          ------------------------------------------------------------
Ilo1          ------------------------------------------------------------
Asp4          ------------------------------------------------------------
Mma1          ------------------------------------------------------------
Pgi1          ------------------------------------------------------------
Bsp1          ------------------------------------------------------------
Efa1          ------------------------------------------------------------
Lla1          ------------------------------------------------------------
Sgo1          ------------------------------------------------------------
Mba1          ------------------------------------------------------------
Dsh1          ------------------------------------------------------------
Ecu1          ------------------------------------------------------------


Sus1          ------------------------------------------------------------
Aba1          ------------------------------------------------------------
Mca1          ------------------------------------------------------------
Tko1          ------------------------------------------------------------
Pfu1          ------------------------------------------------------------
Fba1          ------------------------------------------------------------
Nsp2          ------------------------------------------------------------
Fno1          ------------------------------------------------------------
Cac1          ------------------------------------------------------------
Lhe1          ------------------------------------------------------------
Lre1          ------------------------------------------------------------
Pmo1          ------------------------------------------------------------
Tme1          ------------------------------------------------------------
Sac1          ------------------------------------------------------------
Dol1          ------------------------------------------------------------
Orf1          ------------------------------------------------------------
Rba1          ------------------------------------------------------------
Oin1          ------------------------------------------------------------
Eli1          ------------------------------------------------------------
Sfr1          ------------------------------------------------------------
Pde1          ------------------------------------------------------------
Pcr1          ------------------------------------------------------------
Bli1          ------------------------------------------------------------
Sau1          ------------------------------------------------------------
Sha1          ------------------------------------------------------------
Ssa1          ------------------------------------------------------------
Bsp2          ------------------------------------------------------------
Ccu1          ------------------------------------------------------------
Ame1          ------------------------------------------------------------
Gka1          ------------------------------------------------------------
Emi1          ------------------------------------------------------------
Aae1          ------------------------------------------------------------
Nsp1          ------------------------------------------------------------
Msu1          ------------------------------------------------------------
Cdi1          ------------------------------------------------------------
Cje1          ------------------------------------------------------------
Tva1          ------------------------------------------------------------
Ath1          ------------------------------------------------------------
Vvi1          ------------------------------------------------------------
Clu1          ------------------------------------------------------------
Tca2          ------------------------------------------------------------
Cel3          ------------------------------------------------------------
Hma1          ------------------------------------------------------------
Jsp1          ------------------------------------------------------------
Xor1          ------------------------------------------------------------
Mpo1          ------------------------------------------------------------
Dra1          ------------------------------------------------------------
Asp2          ------------------------------------------------------------
Tfu1          ------------------------------------------------------------
Kra1          ------------------------------------------------------------
Ace1          ------------------------------------------------------------
Aod1          ------------------------------------------------------------
Bad1          ------------------------------------------------------------
Str1          ------------------------------------------------------------
Rer1          ------------------------------------------------------------
Nsp3          ------------------------------------------------------------
```

```
Rsp1          ------------------------------------------------------------
Csa1          ------------------------------------------------------------
Oal1          ------------------------------------------------------------
Mlo1          ------------------------------------------------------------
Lag1          ------------------------------------------------------------
Oan1          ------------------------------------------------------------
Pat1          ------------------------------------------------------------
Neu1          ------------------------------------------------------------
Gbe1          ------------------------------------------------------------
Pbe1          ------------------------------------------------------------
Rxy1          ------------------------------------------------------------
Sav1          ------------------------------------------------------------
Asp1          ------------------------------------------------------------
Mfe1          ------------------------------------------------------------
Sce1          ------------------------------------------------------------
Cbu1          ------------------------------------------------------------
Asp3          ------------------------------------------------------------
Mfl1          ------------------------------------------------------------
Cvi1          ------------------------------------------------------------
Psp1          IAQLIKKLGVSQVDWLGTSMGGLIGMVYAAMPNCPIQKMLINDVGPRIEPEALKRLGSYV
Tca1          ------------------------------------------------------------
Aae2          ------------------------------------------------------------
Cel2          ------------------------------------------------------------
Tca3          ------------------------------------------------------------
Orf2          ------------------------------------------------------------
Bta1          ------------------------------------------------------------
Xla1          ------------------------------------------------------------
Cel1          ------------------------------------------------------------
Tth1          ------------------------------------------------------------
Ddi1          ------------------------------------------------------------
Ath2          ------------------------------------------------------------
Dsp1          ------------------------------------------------------------
Sau2          ------------------------------------------------------------
Cje2          ------------------------------------------------------------
Cfe1          ------------------------------------------------------------
Abu1          ------------------------------------------------------------
Kst1          ------------------------------------------------------------
Lbo1          ------------------------------------------------------------
Pae1          ------------------------------------------------------------
Aph1          ------------------------------------------------------------
Wen1          ------------------------------------------------------------
Mbo1          ------------------------------------------------------------
Mth1          ------------------------------------------------------------
Gbe2          ------------------------------------------------------------
Chu1          ------------------------------------------------------------
Lin1          ------------------------------------------------------------
Rpa1          ------------------------------------------------------------
Van1          ------------------------------------------------------------
Gox1          ------------------------------------------------------------
Afa1          ------------------------------------------------------------
Bph1          ------------------------------------------------------------
Lpn1          ------------------------------------------------------------
Rgr1          ------------------------------------------------------------
Lme1          ------------------------------------------------------------
Sde1          ------------------------------------------------------------
Ilo1          ------------------------------------------------------------
Asp4          ------------------------------------------------------------
Mma1          ------------------------------------------------------------
Pgi1          ------------------------------------------------------------
Bsp1          ------------------------------------------------------------
Efa1          ------------------------------------------------------------
Lla1          ------------------------------------------------------------
Sgo1          ------------------------------------------------------------
Mba1          ------------------------------------------------------------
Dsh1          ------------------------------------------------------------
Ecu1          ------------------------------------------------------------


Sus1          ------------------------------------------------------------
Aba1          ------------------------------------------------------------
Mca1          ------------------------------------------------------------
Tko1          ------------------------------------------------------------
Pfu1          ------------------------------------------------------------
```

```
Fba1      ------------------------------------------------------------
Nsp2      ------------------------------------------------------------
Fno1      ------------------------------------------------------------
Cac1      ------------------------------------------------------------
Lhe1      ------------------------------------------------------------
Lre1      ------------------------------------------------------------
Pmo1      ------------------------------------------------------------
Tme1      ------------------------------------------------------------
Sac1      ------------------------------------------------------------
Dol1      ------------------------------------------------------------
Orf1      ------------------------------------------------------------
Rba1      ------------------------------------------------------------
Oin1      ------------------------------------------------------------
Eli1      ------------------------------------------------------------
Sfr1      ------------------------------------------------------------
Pde1      ------------------------------------------------------------
Pcr1      ------------------------------------------------------------
Bli1      ------------------------------------------------------------
Sau1      ------------------------------------------------------------
Sha1      ------------------------------------------------------------
Ssa1      ------------------------------------------------------------
Bsp2      ------------------------------------------------------------
Ccu1      ------------------------------------------------------------
Ame1      ------------------------------------------------------------
Gka1      ------------------------------------------------------------
Emi1      ------------------------------------------------------------
Aae1      ------------------------------------------------------------
Nsp1      ------------------------------------------------------------
Msu1      ------------------------------------------------------------
Cdi1      ------------------------------------------------------------
Cje1      ------------------------------------------------------------
Tva1      ------------------------------------------------------------
Ath1      ------------------------------------------------------------
Vvi1      ------------------------------------------------------------
Clu1      ------------------------------------------------------------
Tca2      ------------------------------------------------------------
Cel3      ------------------------------------------------------------
Hma1      ------------------------------------------------------------
Jsp1      ------------------------------------------------------------
Xor1      ------------------------------------------------------------
Mpo1      ------------------------------------------------------------
Dra1      ------------------------------------------------------------
Asp2      ------------------------------------------------------------
Tfu1      ------------------------------------------------------------
Kra1      ------------------------------------------------------------
Ace1      ------------------------------------------------------------
Aod1      ------------------------------------------------------------
Bad1      ------------------------------------------------------------
Str1      ------------------------------------------------------------
Rer1      ------------------------------------------------------------
Nsp3      ------------------------------------------------------------
Rsp1      ------------------------------------------------------------
Csa1      ------------------------------------------------------------
Oal1      ------------------------------------------------------------
Mlo1      ------------------------------------------------------------
Lag1      ------------------------------------------------------------
Oan1      ------------------------------------------------------------
Pat1      ------------------------------------------------------------
Neu1      ------------------------------------------------------------
Gbe1      ------------------------------------------------------------
Pbe1      ------------------------------------------------------------
Rxy1      ------------------------------------------------------------
Sav1      ------------------------------------------------------------
Asp1      ------------------------------------------------------------
Mfe1      ------------------------------------------------------------
Sce1      ------------------------------------------------------------
Cbu1      ------------------------------------------------------------
Asp3      ------------------------------------------------------------
Mfl1      ------------------------------------------------------------
Cvi1      ------------------------------------------------------------
Psp1      GQPFVFGNRADALERLNKICASFGEHTPQEWEIYNGPMLIQKDEQWIMHYDPNISVPFAS
Tca1      ------------------------------------------------------------
Aae2      ------------------------------------------------------------
```

```
Cel2            ------------------------------------------------------------
Tca3            ------------------------------------------------------------
Orf2            ------------------------------------------------------------
Bta1            ------------------------------------------------------------
Xla1            ------------------------------------------------------------
Cel1            ------------------------------------------------------------
Tth1            ------------------------------------------------------------
Ddi1            ------------------------------------------------------------
Ath2            ------------------------------------------------------------
Dsp1            ------------------------------------------------------------
Sau2            ------------------------------------------------------------
Cje2            ------------------------------------------------------------
Cfe1            ------------------------------------------------------------
Abu1            ------------------------------------------------------------
Kst1            ------------------------------------------------------------
Lbo1            ------------------------------------------------------------
Pae1            ------------------------------------------------------------
Aph1            ------------------------------------------------------------
Wen1            ------------------------------------------------------------
Mbo1            ------------------------------------------------------------
Mth1            ------------------------------------------------------------
Gbe2            ------------------------------------------------------------
Chu1            ------------------------------------------------------------
Lin1            ------------------------------------------------------------
Rpa1            ------------------------------------------------------------
Van1            ------------------------------------------------------------
Gox1            ------------------------------------------------------------
Afa1            ------------------------------------------------------------
Bph1            ------------------------------------------------------------
Lpn1            ------------------------------------------------------------
Rgr1            ------------------------------------------------------------
Lme1            ------------------------------------------------------------
Sde1            ------------------------------------------------------------
Ilo1            ------------------------------------------------------------
Asp4            ------------------------------------------------------------
Mma1            ------------------------------------------------------------
Pgi1            ------------------------------------------------------------
Bsp1            ------------------------------------------------------------
Efa1            ------------------------------------------------------------
Lla1            ------------------------------------------------------------
Sgo1            ------------------------------------------------------------
Mba1            ------------------------------------------------------------
Dsh1            ------------------------------------------------------------
Ecu1            ------------------------------------------------------------

Sus1            ------------------------------------------------------------
Aba1            ------------------------------------------------------------
Mca1            ------------------------------------------------------------
Tko1            ------------------------------------------------------------
Pfu1            ------------------------------------------------------------
Fba1            ------------------------------------------------------------
Nsp2            ------------------------------------------------------------
Fno1            ------------------------------------------------------------
Cac1            ------------------------------------------------------------
Lhe1            ------------------------------------------------------------
Lre1            ------------------------------------------------------------
Pmo1            ------------------------------------------------------------
Tme1            ------------------------------------------------------------
Sac1            ------------------------------------------------------------
Dol1            ------------------------------------------------------------
Orf1            ------------------------------------------------------------
Rba1            ------------------------------------------------------------
Oin1            ------------------------------------------------------------
Eli1            ------------------------------------------------------------
Sfr1            ------------------------------------------------------------
Pde1            ------------------------------------------------------------
Pcr1            ------------------------------------------------------------
Bli1            ------------------------------------------------------------
Sau1            ------------------------------------------------------------
Sha1            ------------------------------------------------------------
Ssa1            ------------------------------------------------------------
Bsp2            ------------------------------------------------------------
```

```
Ccu1            ------------------------------------------------------------
Ame1            ------------------------------------------------------------
Gka1            ------------------------------------------------------------
Emi1            ------------------------------------------------------------
Aae1            ------------------------------------------------------------
Nsp1            ------------------------------------------------------------
Msu1            ------------------------------------------------------------
Cdi1            ------------------------------------------------------------
Cje1            ------------------------------------------------------------
Tva1            ------------------------------------------------------------
Ath1            ------------------------------------------------------------
Vvi1            ------------------------------------------------------------
Clu1            ------------------------------------------------------------
Tca2            ------------------------------------------------------------
Cel3            ------------------------------------------------------------
Hma1            ------------------------------------------------------------
Jsp1            ------------------------------------------------------------
Xor1            ------------------------------------------------------------
Mpo1            ------------------------------------------------------------
Dra1            ------------------------------------------------------------
Asp2            ------------------------------------------------------------
Tfu1            ------------------------------------------------------------
Kra1            ------------------------------------------------------------
Ace1            ------------------------------------------------------------
Aod1            ------------------------------------------------------------
Bad1            ------------------------------------------------------------
Str1            ------------------------------------------------------------
Rer1            ------------------------------------------------------------
Nsp3            ------------------------------------------------------------
Rsp1            ------------------------------------------------------------
Csa1            ------------------------------------------------------------
Oal1            ------------------------------------------------------------
Mlo1            ------------------------------------------------------------
Lag1            ------------------------------------------------------------
Oan1            ------------------------------------------------------------
Pat1            ------------------------------------------------------------
Neu1            ------------------------------------------------------------
Gbe1            ------------------------------------------------------------
Pbe1            ------------------------------------------------------------
Rxy1            ------------------------------------------------------------
Sav1            ------------------------------------------------------------
Asp1            ------------------------------------------------------------
Mfe1            ------------------------------------------------------------
Sce1            ------------------------------------------------------------
Cbu1            ------------------------------------------------------------
Asp3            ------------------------------------------------------------
Mfl1            ------------------------------------------------------------
Cvi1            ------------------------------------------------------------
Psp1            VNPIMAKAGEMAMWHAFKQIRIPMLIVRGGDSDLLSAATVAEMCKINPYARSIEIPHVGH
Tca1            ------------------------------------------------------------
Aae2            ------------------------------------------------------------
Cel2            ------------------------------------------------------------
Tca3            ------------------------------------------------------------
Orf2            ------------------------------------------------------------
Bta1            ------------------------------------------------------------
Xla1            ------------------------------------------------------------
Cel1            ------------------------------------------------------------
Tth1            ------------------------------------------------------------
Ddi1            ------------------------------------------------------------
Ath2            ------------------------------------------------------------
Dsp1            ------------------------------------------------------------
Sau2            ------------------------------------------------------------
Cje2            ------------------------------------------------------------
Cfe1            ------------------------------------------------------------
Abu1            ------------------------------------------------------------
Kst1            ------------------------------------------------------------
Lbo1            ------------------------------------------------------------
Pae1            ------------------------------------------------------------
Aph1            ------------------------------------------------------------
Wen1            ------------------------------------------------------------
Mbo1            ------------------------------------------------------------
Mth1            ------------------------------------------------------------
Gbe2            ------------------------------------------------------------
```

```
Chu1            ------------------------------------------------------------
Lin1            ------------------------------------------------------------
Rpa1            ------------------------------------------------------------
Van1            ------------------------------------------------------------
Gox1            ------------------------------------------------------------
Afa1            ------------------------------------------------------------
Bph1            ------------------------------------------------------------
Lpn1            ------------------------------------------------------------
Rgr1            ------------------------------------------------------------
Lme1            ------------------------------------------------------------
Sde1            ------------------------------------------------------------
Ilo1            ------------------------------------------------------------
Asp4            ------------------------------------------------------------
Mma1            ------------------------------------------------------------
Pgi1            ------------------------------------------------------------
Bsp1            ------------------------------------------------------------
Efa1            ------------------------------------------------------------
Lla1            ------------------------------------------------------------
Sgo1            ------------------------------------------------------------
Mba1            ------------------------------------------------------------
Dsh1            ------------------------------------------------------------
Ecu1            ------------------------------------------------------------


Sus1            ------------------
Aba1            ------------------
Mca1            ------------------
Tko1            ------------------
Pfu1            ------------------
Fba1            ------------------
Nsp2            ------------------
Fno1            ------------------
Cac1            ------------------
Lhe1            ------------------
Lre1            ------------------
Pmo1            ------------------
Tme1            ------------------
Sac1            ------------------
Dol1            ------------------
Orf1            ------------------
Rba1            ------------------
Oin1            ------------------
Eli1            ------------------
Sfr1            ------------------
Pde1            ------------------
Pcr1            ------------------
Bli1            ------------------
Sau1            ------------------
Sha1            ------------------
Ssa1            ------------------
Bsp2            ------------------
Ccu1            ------------------
Ame1            ------------------
Gka1            ------------------
Emi1            ------------------
Aae1            ------------------
Nsp1            ------------------
Msu1            ------------------
Cdi1            ------------------
Cje1            ------------------
Tva1            ------------------
Ath1            ------------------
Vvi1            ------------------
Clu1            ------------------
Tca2            ------------------
Cel3            ------------------
Hma1            ------------------
Jsp1            ------------------
Xor1            ------------------
Mpo1            ------------------
Dra1            ------------------
Asp2            ------------------
Tfu1            ------------------
```

```
Kra1            ------------------
Ace1            ------------------
Aod1            ------------------
Bad1            ------------------
Str1            ------------------
Rer1            ------------------
Nsp3            ------------------
Rsp1            ------------------
Csa1            ------------------
Oal1            ------------------
Mlo1            ------------------
Lag1            ------------------
Oan1            ------------------
Pat1            ------------------
Neu1            ------------------
Gbe1            ------------------
Pbe1            ------------------
Rxy1            ------------------
Sav1            ------------------
Asp1            ------------------
Mfe1            ------------------
Sce1            ------------------
Cbu1            ------------------
Asp3            ------------------
Mfl1            ------------------
Cvi1            ------------------
Psp1            APAFVKPEQIALAKEFFS
Tca1            ------------------
Aae2            ------------------
Cel2            ------------------
Tca3            ------------------
Orf2            ------------------
Bta1            ------------------
Xla1            ------------------
Cel1            ------------------
Tth1            ------------------
Ddi1            ------------------
Ath2            ------------------
Dsp1            ------------------
Sau2            ------------------
Cje2            ------------------
Cfe1            ------------------
Abu1            ------------------
Kst1            ------------------
Lbo1            ------------------
Pae1            ------------------
Aph1            ------------------
Wen1            ------------------
Mbo1            ------------------
Mth1            ------------------
Gbe2            ------------------
Chu1            ------------------
Lin1            ------------------
Rpa1            ------------------
Van1            ------------------
Gox1            ------------------
Afa1            ------------------
Bph1            ------------------
Lpn1            ------------------
Rgr1            ------------------
Lme1            ------------------
Sde1            ------------------
Ilo1            ------------------
Asp4            ------------------
Mma1            ------------------
Pgi1            ------------------
Bsp1            ------------------
Efa1            ------------------
Lla1            ------------------
Sgo1            ------------------
Mba1            ------------------
Dsh1            ------------------
Ecu1            ------------------
```
