# Supplementary material for: Animal Ca2+ release-activated Ca2+ (CRAC) channels appear to be homologous to and derived from the ubiquitous cation diffusion facilitators
Source: BMC Res Notes. 2010 Jun 3;3:158. doi: 10.1186/1756-0500-3-158 (PMC2894845; doi:10.1186/1756-0500-3-158)
Supplement: Additional file 4 — Table S2 - List of Orai protein sequences from the CRAC-C family included in this study. Proteins are listed according to cluster number as indicated in Figure S2A. Within each cluster, proteins are presented according to their position in the cluster. [file 1756-0500-3-158-S4.PDF]

Table S1

Orai proteins of the CRAC-C family included in this study

Abbreviation	Organism	Size	GI No.
Cluster 1: Chordata Orai-1			
Hsa1	*Homo sapiens*	301	97180269
Mmu1	*Mus musculus*	304	81897819
Gga1	*Gallus gallus*	226	82082365
Xla1	*Xenopus laevis*	258	82194530
Dre1	*Danio rerio*	222	97180268
Cluster 2: Chordata Orai-2			
Mmu2	*Mus musculus*	250	81896067
Gga2	*Gallus gallus*	257	82082722
Hsa2	*Homo sapiens*	254	74732728
Xla2	*Xenopus laevis*	257	82186030
Cluster 3: Chordata Orai-3			
Mmu3	*Mus musculus*	290	81885385
Hsa3	*Homo sapiens*	295	74732916
Cluster 4: Echinodermata			
Spu1	*Strongylocentrotus purpuratus*	201	115770361
Cluster 5: Arthropoda			
Dme1	*Drosophila melanogaster*	351	74866986
Aae1	*Aedes aegypti*	299	108867881
Aga1	*Anopheles gambiae str. PEST*	223	58385444
Ame1	*Apis mellifera*	217	110768003
Tca1	*Tribolium castaneum*	249	91095137
Nvi1	*Nasonia vitripennis*	368	156549609
Cluster 6: Nematoda			
Cbr1	*Caenorhabditis briggsae*	300	74784575
Cel1	*Caenorhabditis elegans*	293	2496887
