# Supplementary material for: Animal Ca2+ release-activated Ca2+ (CRAC) channels appear to be homologous to and derived from the ubiquitous cation diffusion facilitators
Source: BMC Res Notes. 2010 Jun 3;3:158. doi: 10.1186/1756-0500-3-158 (PMC2894845; doi:10.1186/1756-0500-3-158)
Supplement: Additional file 5 — Table S2 - List of Stim protein sequences from the CRAC-C family included in this study. Proteins are listed according to cluster number as indicated in Figure S2B. Within each cluster, proteins are presented according to their position in the cluster. [file 1756-0500-3-158-S5.PDF]

Table S2

Stim proteins of the CRAC-C family included in this study

Abbreviation	Organism	Size	GI No.
Cluster 1: Chordata Stim-1			
Hsa1	*Homo sapiens*	685	17368447
Mmu1	*Mus musculus*	685	17368305
Gga1	*Gallus gallus*	612	71895083
Xtr1	*Xenopus tropicalis*	664	45433580
Dre1	*Danio rerio*	676	113677986
Cluster 2: Chordata Stim-2			
Mmu2	*Mus musculus*	746	116242804
Gga2	*Gallus gallus*	740	118090646
Hsa2	*Homo sapiens*	746	17369338
Xtr2	*Xenopus tropicalis*	404	89266839
Cluster 3: Echinodermata			
Spu1	*Strongylocentrotus purpuratus*	514	115973379
Cluster 4: Arthropoda			
Dme1	*Drosophila melanogaster*	570	17368342
Aae1	*Aedes aegypti*	455	108869895
Aga1	*Anopheles gambiae str. PEST*	476	119115237
Ame1	*Apis mellifera*	727	110757132
Tca1	*Tribolium castaneum*	493	91081005
Nvi1	*Nasonia vitripennis*	661	156552163
Cluster 5: Nematoda			
Cbr1	*Caenorhabditis briggsae*	534	39588863
Cel1	*Caenorhabditis elegans*	530	25144975
