# Supplementary material for: Animal Ca2+ release-activated Ca2+ (CRAC) channels appear to be homologous to and derived from the ubiquitous cation diffusion facilitators
Source: BMC Res Notes. 2010 Jun 3;3:158. doi: 10.1186/1756-0500-3-158 (PMC2894845; doi:10.1186/1756-0500-3-158)
Supplement: Additional file 6 — Table S3 - List of Cation Diffusion Facilitator sequences included in this study. Proteins are listed according to cluster number as indicated in Figure S2C. Within each cluster, proteins are presented according to their position in the cluster. [file 1756-0500-3-158-S6.PDF]

Table S3

Selected members of the CDF family included in this study

Abbreviation	Organism	Size	GI Nc
Cluster 1			
Sus1	*Solibacter usitatus Ellin6076*	312	11662
Aba1	*Acidobacteria bacterium Ellin345*	312	94971
Mca1	*Methylococcus capsulatus str. Bath*	314	53803
Dsh1	*Dinoroseobacter shibae DFL 12*	328	15904
Cluster 2			
Csa1	*Chromohalobacter salexigens DSM 3043*	327	92112
Oal1	*Oceanicaulis alexandrii HTCC2633*	321	83859
Mlo1	*Mesorhizobium loti MAFF303099*	365	13472
Lag1	*Labrenzia aggregata IAM 12614*	323	11859
Oan1	*Ochrobactrum anthropi ATCC 49188*	352	15301
Pat1	*Pectobacterium atrosepticum SCRI1043*	320	50120
Neu1	*Nitrosomonas eutropha C91*	318	11433
Gbe1	*Granulibacter bethesdensis CGDNIH1*	344	11432
Pbe1	*Parvularcula bermudensis HTCC2503*	339	84703
Aph1	*Anaplasma phagocytophilum HZ*	301	88606
Wen1	*Wolbachia endosymbiont of Drosophila ananassae*	322	58698
Mbo1	*Candidatus Methanoregula boonei 6A8*	317	15415
Mth1	*Methanosaeta thermophila PT*	298	11675
Gbe2	*Geobacter bemidjiensis Bem*	335	14561
Cluster 3			
Tko1	*Thermococcus kodakarensis KOD1*	297	57641
Pfu1	*Pyrococcus furiosus DSM 3638*	295	18976
Fno1	*Fervidobacterium nodosum Rt17-B1*	324	15424
Cac1	*Clostridium acetobutylicum ATCC 824*	303	15895
Fba1	*Flavobacteriales bacterium ALC-1*	305	16378
Nsp2	*Nitratiruptor sp. SB155-2*	301	15298
Lhe1	*Lactobacillus helveticus DPC 4571*	299	16150
Lre1	*Lactobacillus reuteri 100-23*	306	92090
Pmo1	*Petrotoga mobilis SJ95*	300	16090
Tme1	*Thermosipho melanesiensis BI429*	294	15002
Sac1	*Syntrophus aciditrophicus SB*	323	85860
Dol1	*Desulfococcus oleovorans Hxd3*	307	15852
Pde1	*Paracoccus denitrificans PD1222*	311	69934
Sfr1	*Shewanella frigidimarina NCIMB 400*	300	11456

Orf1	*alpha proteobacterium BAL199*	319	16379
Rba1	*Rhodopirellula baltica SH 1*	331	32475
Oin1	*Oceanibulbus indolifex HEL-45*	322	16374
Eli1	*Erythrobacter litoralis HTCC2594*	317	85373
Cluster 4			
Chu1	*Cytophaga hutchinsonii ATCC 33406*	303	11063
Lin1	*Leptospira interrogans serovar Lai str. 56601*	308	24216
Lme1	*Leuconostoc mesenteroides subsp. mesenteroides ATCC 8293*	305	11661
Afa1	*Alcaligenes faecalis*	303	42741
Rpa1	*Rhodopseudomonas palustris BisB5*	372	91974
Van1	*Vibrio angustum S14*	301	90578
Gox1	*Gluconobacter oxydans 621H*	351	58040
Bph1	*Burkholderia phymatum STM815*	373	11802
Lpn1	*Legionella pneumophila str. Lens*	307	54295
Rgr1	*Rickettsiella grylli*	298	16087
Cluster 5			
Aae1	*Aquifex aeolicus VF5*	308	15606
Emi1	*Elusimicrobium minutum Pei191*	327	16369
Gka1	*Geobacillus kaustophilus HTA426*	315	56419
Pcr1	*Psychrobacter cryohalolentis K5*	398	93006
Bli1	*Bacillus licheniformis ATCC 14580*	311	52081
Ccu1	*Campylobacter curvus 525.92*	378	15417
Ssa1	*Staphylococcus saprophyticus subsp. saprophyticus ATCC 15305*	321	73662
Sau1	*Staphylococcus aureus*	326	34455
Sha1	*Staphylococcus haemolyticus JCSC1435*	316	70725
Bsp2	*Bacillus sp. B14905*	315	12665
Ame1	*Apis mellifera*	186	11077
Nsp1	*Nodularia spumigena CCY 9414*	326	11951
Msu1	*Mannheimia succiniciproducens MBEL55E*	229	52425
Cluster 6			
Sce1	*Sorangium cellulosum 'So ce 56'*	363	16245
Asp1	*Anaeromyxobacter sp. Fw109-5*	342	15300
Mfe1	*Mariprofundus ferrooxydans PV-1*	301	11477
Cvi1	*Chromobacterium violaceum ATCC 12472*	336	34499
Psp1	*Polynucleobacter sp. QLW-P1DMWA-1*	626	14558
Mfl1	*Methylobacillus flagellatus KT*	325	91776
Cbu1	*Coxiella burnetii 'MSU Goat Q177'*	297	15320
Asp3	*Azoarcus sp. BH72*	410	11989

Cluster 7			
Cdi1	*Corynebacterium diphtheriae NCTC 13129*	307	3823[illegible]
Cje1	*Corynebacterium jeikeium K411*	373	6853[illegible]
Mpo1	*Methylobacterium populi BJ001*	313	1638[illegible]
Dra1	*Deinococcus radiodurans R1*	325	1580[illegible]
Jsp1	*Janthinobacterium sp. Marseille*	316	1529[illegible]
Xor1	*Xanthomonas oryzae pv. oryzae KACC10331*	331	5858[illegible]
Str1	*Salinispora tropica CNB-440*	305	1455[illegible]
Rer1	*Rhodococcus erythropolis*	316	3386[illegible]
Nsp3	*Nocardioides sp. JS614*	305	1197[illegible]
Rsp1	*Rhodococcus sp. RHA1*	308	1110[illegible]
Asp2	*Arthrobacter sp. FB24*	308	1166[illegible]
Tfu1	*Thermobifida fusca YX*	300	7216[illegible]
Kra1	*Kineococcus radiotolerans SRS30216*	356	1572[illegible]
Ace1	*Acidothermus cellulolyticus 11B*	306	1179[illegible]
Aod1	*Actinomyces odontolyticus ATCC 17982*	343	1545[illegible]
Bad1	*Bifidobacterium adolescentis ATCC 15703*	313	1190[illegible]
Rxy1	*Rubrobacter xylanophilus DSM 9941*	314	1088[illegible]
Sav1	*Streptomyces avermitilis MA-4680*	371	2983[illegible]
Cluster 8			
Hma1	*Haloarcula marismortui ATCC 43049*	311	5537[illegible]
Tva1	*Trichomonas vaginalis G3*	436	1233[illegible]
Ath1	*Arabidopsis thaliana*	300	4895[illegible]
Vvi1	*Vitis vinifera*	521	1573[illegible]
Cel3	*Caenorhabditis elegans*	746	3256[illegible]
Clu1	*Canis lupus familiaris*	766	7394[illegible]
Tca2	*Tribolium castaneum*	346	9109[illegible]
Cluster 9			
Tth1	*Tetrahymena thermophila SB210*	352	1183[illegible]
Ecu1	*Encephalitozoon cuniculi GB-M1*	334	1907[illegible]
Cel1	*Caenorhabditis elegans*	410	1755[illegible]
Tca3	*Tribolium castaneum*	337	9108[illegible]
Cel2	*Caenorhabditis elegans*	382	1756[illegible]
Tca1	*Tribolium castaneum*	429	9107[illegible]
Aae2	*Aedes aegypti*	365	1571[illegible]
Xla1	*Xenopus laevis*	375	1478[illegible]
Bta1	*Bos taurus*	611	7661[illegible]
Ddi1	*Dictyostelium discoideum AX4*	543	6681[illegible]
Ath2	*Arabidopsis thaliana*	432	4256[illegible]
Cluster 10			

Mba1	*Methanosarcina barkeri str. Fusaro*	301	7367[illegible]
Asp4	*Algoriphagus sp. PR1*	277	1266[illegible]
Sde1	*Shewanella denitrificans OS217*	365	9179[illegible]
Ilo1	*Idiomarina loihiensis L2TR*	295	5646[illegible]
Mma1	*Microscilla marina ATCC 23134*	331	1240[illegible]
Pgi1	*Porphyromonas gingivalis W83*	321	3454[illegible]
Bsp1	*Bacillus sp. SG-1*	286	1491[illegible]
Efa1	*Enterococcus faecium*	307	4252[illegible]
Lla1	*Lactococcus lactis subsp. cremoris MG1363*	305	1256[illegible]
Sgo1	*Streptococcus gordonii str. Challis substr. CH1*	296	1571[illegible]
Cluster 11			
Dsp1	*Dehalococcoides sp. CBDB1*	316	7374[illegible]
Sau2	*Staphylococcus aureus subsp. aureus str. Newman*	354	1512[illegible]
Kst1	*Candidatus Kuenenia stuttgartiensis*	302	9120[illegible]
Abu1	*Arcobacter butzleri RM4018*	351	1577[illegible]
Cje2	*Campylobacter jejuni subsp. jejuni CG8486*	324	1489[illegible]
Cfe1	*Campylobacter fetus subsp. fetus 82-40*	314	1184[illegible]
Lbo1	*Leptospira borgpetersenii serovar Hardjo-bovis L550*	320	1163[illegible]
Pae1	*Prosthecochloris aestuarii DSM 271*	319	6855[illegible]
