# Supplementary figures and images for: Animal Ca2+ release-activated Ca2+ (CRAC) channels appear to be homologous to and derived from the ubiquitous cation diffusion facilitators
Source: BMC Res Notes. 2010 Jun 3;3:158. doi: 10.1186/1756-0500-3-158 (PMC2894845; doi:10.1186/1756-0500-3-158)

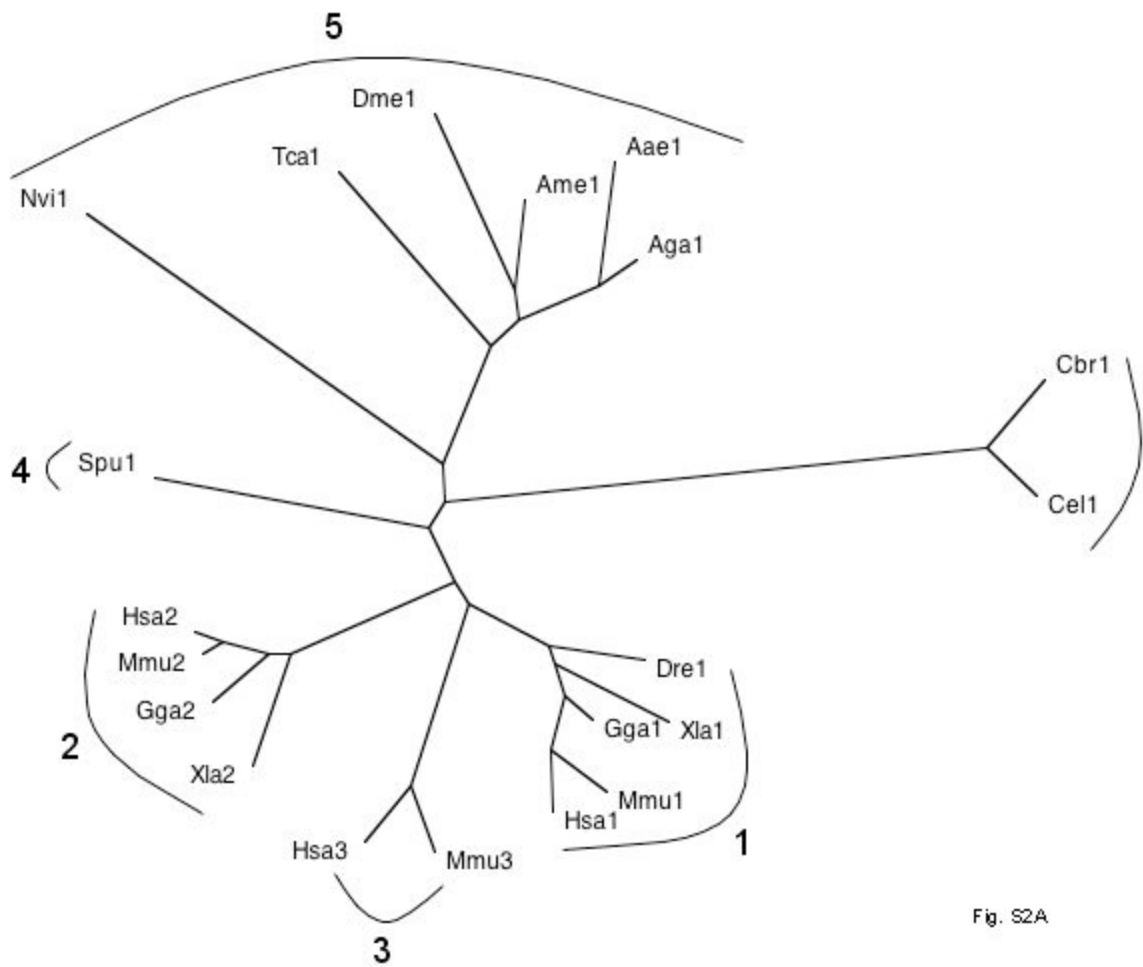

Fig. S2A

Supplement: Additional file 7 — S2A - Phylogenetic tree of Orai proteins. Protein abbreviations are as indicated in table S1. Clusters are labeled 1-6. The tree was drawn using the TreeView (neighbor joining) program, based on the multiple alignment shown in Figure S1A. [file 1756-0500-3-158-S7.PDF]

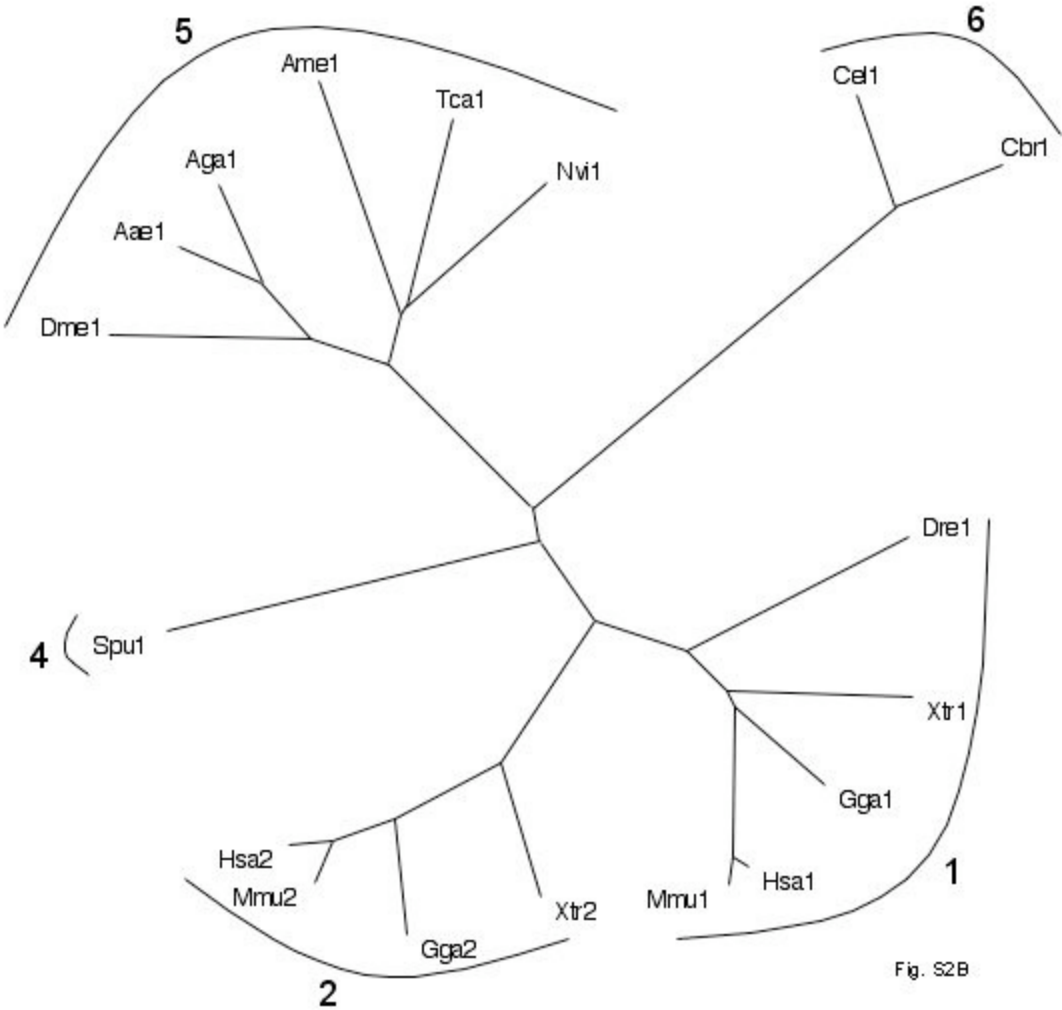

Supplement: Additional file 8 — S2B - Phylogenetic tree of Stim proteins. Protein abbreviations are as indicated in table S2. Clusters are labeled 1, 2 and 4-6, corresponding to the clusters in figure S2A. The tree was drawn using the TreeView (neighbor joining) program, based on the multiple alignment shown in Figure S1B. [file 1756-0500-3-158-S8.PDF]

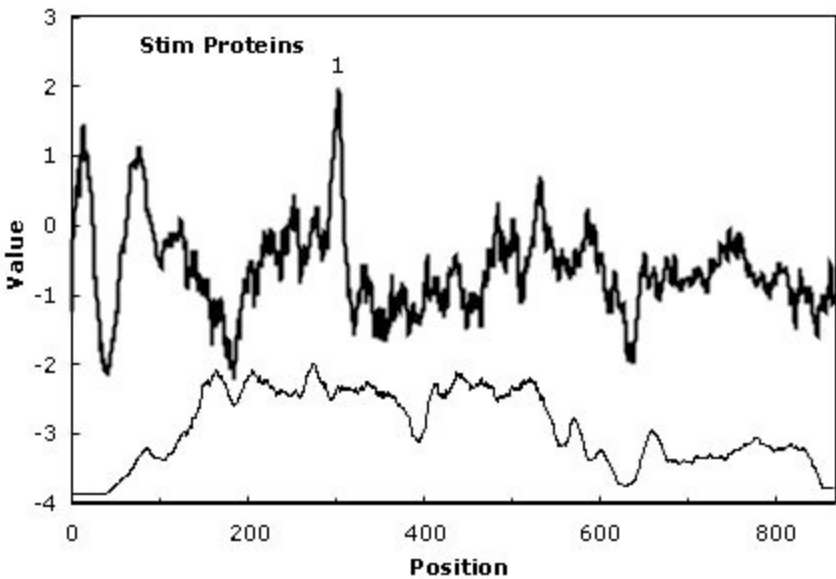

Fig S3

Supplement: Additional file 9 — S3 - Average hydropathy (dark line, top) and average similarity (light line, bottom) plots of Stim protein homologues (see Table S2). This plot was generated with the AveHas program (Zhai and Saier, 2001) based on the multiple alignment shown in figure S1B. [file 1756-0500-3-158-S9.PDF]

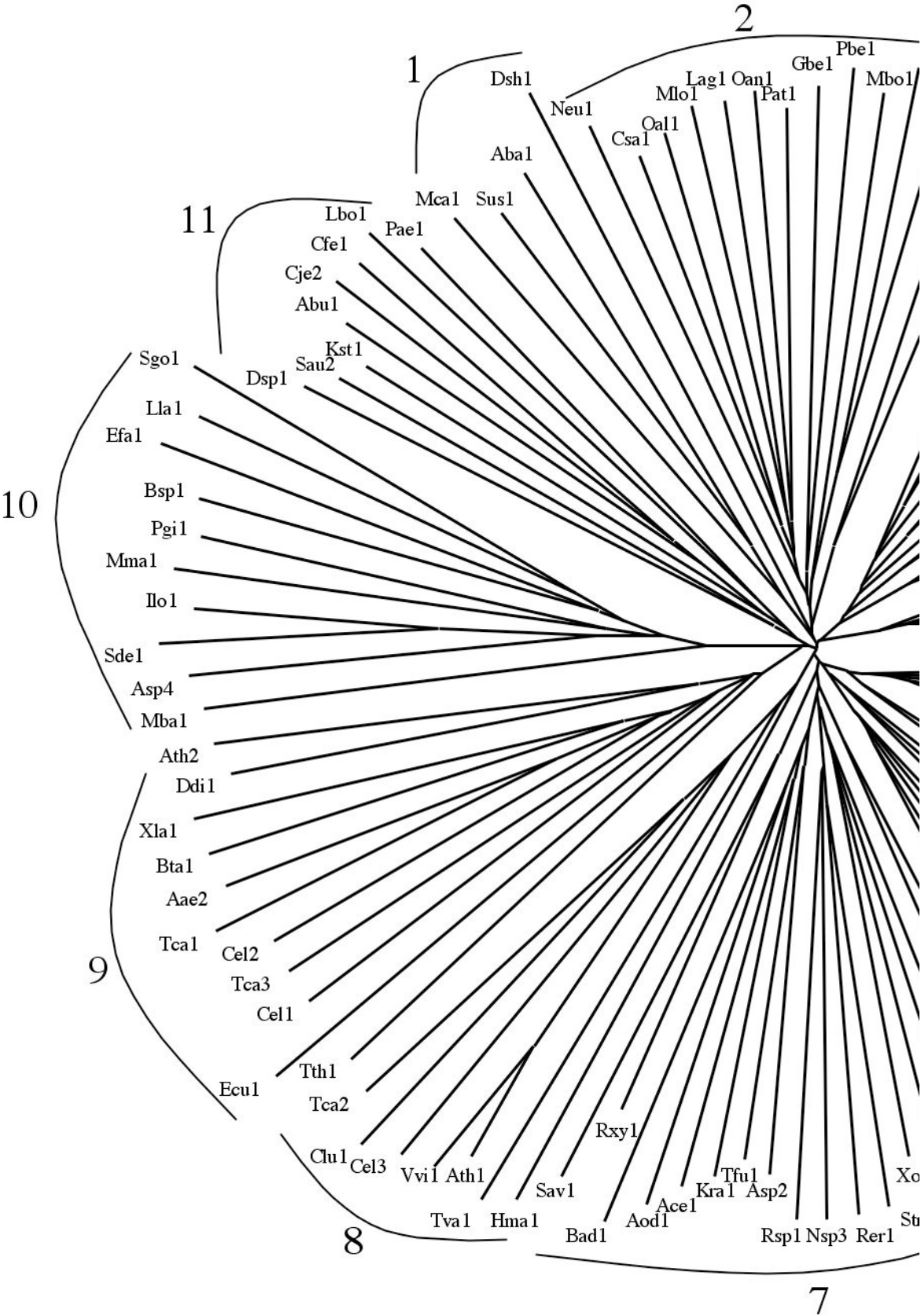

Supplement: Additional file 10 — S2C - Phylogenetic tree of CDF proteins. Protein abbreviations are as indicated in Table S3. Clusters are labeled 1-11. The tree was drawn using the TreeView (neighbor joining) program, based on the multiple alignment shown in Figure S1C. [file 1756-0500-3-158-S10.PDF]
