# Supplementary material for: Animal Ca2+ release-activated Ca2+ (CRAC) channels appear to be homologous to and derived from the ubiquitous cation diffusion facilitators
Source: BMC Res Notes. 2010 Jun 3;3:158. doi: 10.1186/1756-0500-3-158 (PMC2894845; doi:10.1186/1756-0500-3-158)
Supplement: Additional file 11 — S4 - Multiple alignment of eleven Orai homologues with eleven CDF homologues. The two proteins used as query sequences in BLAST searches were the C. elegans Orai homologue (NP_497230) and the Pyrococcus furiosus CDF homologue (AAL80682). These two proteins plus 10 homologues retrieved from each search with good scores, each from a different species, were included in the ClustalX multiple alignment (see Methods section). With 116 positions shown, there are three positions where only identities (a single residue) are present (asterisks), eighteen positions where only close similarities are present (colons), and eleven positions where only more distant similarities are present (dots) as defined by the ClustalX program (see Methods). Thus, 32 positions, or 28% of all positions, show identities and similarities in all twenty two proteins included in the alignment. The species name, the protein genbank ID#, the starting residue number, the sequence included in the alignment, and the final residue number for each protein are shown in the figure. [file 1756-0500-3-158-S11.PDF]

Organism	GI#	Start (aa)	Alignment	Stop (aa)
Caenorhabditis elegans	211593603	17	RAQLKASSRTSALLAGFAMVCLVELQYDQ-----STPKPLLIVLGVVTSLLVSVHLLALMMSTCILPYMEATG--------CTQDSPHIKLKFYIDLSWLFSTCIGLLLFLVEIGVI	120
Caenorhabditis briggsae	268575982	120	KAQLKASSRTSALLAGFAMVCLVELQYDD-----STSKPLLIVLGVVTSLLVSVHLLALMMSTCILPYMEATG--------CTQDSPHLKLKFYIDLSWLFSTCIGLLLFLVEIGVI	223
Brugia malayi	170577325	235	RAQLKASSRTSALLAGFAMVALVELQYER-----TTPPFLLITLGVVTTLLVSVHLLALMMSTCILPYIEANG--------CTQDSPHIRLKFYIDLSWLFSTCIGLVLFLIEIGII	338
Macaca mulatta	109128264	58	RAKLKASSRTSALLSGFAMVAMVEVQLES---DHEYPPGLLVAFSACTTVLVAVHLFALMVSTCLLPHIEAVSNIHNLN--SVHQSPHQRLHRYVELAWGFSTALGTFLFLAEVVLV	169
Xenopus laevis	148225707	55	RAKLKASSRTSALLSGFAMVAMVEVQLEP---NHAYPPGLLIAFSACTTVLVAVHLFALMVSTCILPNIEAVSNVHNLN--SVKESPHERMHHHIELAWAFSTVIGTLLFLAEVVLL	166
Strongylocentrotus purpuratus	115770361	19	RAKLKASSRTSALLAGFAMVAMVEVQLSATKETNAYDP-LMIAFSINTTILVVVHMAALLISTCILPNIEAVSNVHNVN--AVQESPHNSLAFYIEMSWIFSTVLGIFLFLVEIILL	132
Ixodes scapularis	242000244	53	RAKLKASSRTSALLSGFAMVAMVEIQLSK-----SIPPQLLIAFSVCTTLLVSVHMLALMISTCILPNLEAVASVHGIA--AVSESPHEKMHLYIETAWAFSTVFGILLFMSEIAIL	162
Nematostella vectensis	156384154	10	RGKLKASSRTSALMSGFAMIAMVEIQLEE-----GIPGGLLIAFSVMTTILISVHVFALMISVCILPNIESVANTHQSSGLMVHDSPHEKMHLYVELAWIFSTGLGTLLFLGEIGIL	121
Bombyx mori	290563441	28	RAKLKASSKTSALLSGFAMVAMVEVQLNPAP--TAVPKEMLVAFTVCTTLLVAVHMLALMISTCILPNIEAVGNLHSIA--LVHESPHERLHWYIEVAWAFSTLLGLILFLIEIAIL	140
Drosophila mojavensis	195121700	168	RAKLKASSKTSALLSGFAMVAMVEVQLDKD---TGVPPGMLVAFAICTTLLVAVHMLALMISTCILPNIETVCNLHSIS--LVHESPHERLHWYIETAWAFSTLLGLILFLVEIAIL	279
Nasonia vitripennis	156544243	23	KAKLKASSKTSALLAGFAMVAMVELQLNNW---NDIPEKMIILFTVCTTLLVAVHMLALMISTCILPNIEAVSSMHCIT--FINESPHERLHWYIEVAWAFSTVLGLFLFLVEIAIL	134
			Orai TMS #1 / Orai TMS #2 / Orai TMS#3	
			CDF TMS #3 / CDF TMS #4 / CDF TMS #5	
Pyrococcus furiosus	18976930	76	RAEILVAFVNSAILVGVALFLIVEAYKRFK---TPEPINGPLMFSVALIGFLANLISVLLLHEHSHENINVRSAYLHLLS-DTLSSVAVVLGGIAIIKWNAIWVDPLLSALISIYIL	188
Thermococcus kodakarensis	57641944	77	RAEILVAFVNSAVLVGVALFLLVEAYKRFK---NPEPIDGPLMLGVALIGLFANLISVLLLHEHAHESMNVRSAYLHLLS-DTLSSVAVVIGGIAIIRWDVLWIDPLVTVLISVYIL	189
Pyrococcus horikoshii	14590752	75	RAEILVAFANSAVLVGVSLFLIFEAYKRFK---SPEPIKGSLMLFVALIGLFANLASVILLHEHAHESMNIRSAYLHLVS-DSLSSIAVVLGGIAITKWNVFWIDPLITVLISLYIL	187
Thermococcus gammatolerans	240102887	80	RAEILVAFVNSAVLVGVSLLLLVEAYRRYK---NPKPIDGPLMLVVALIGLFANLFSVLLLHSHAHG-LNVRSAYLHLLS-DTLSSVAVVAGGIAIIKWNVLWIDPLITVLIALYIL	191
Thermococcus sibiricus	242399672	77	RVEILVAFMNSAVLVGVSLLLLVEAYRRFK---NPEPIDGPLMLGVALIGLFANLFSVLLLHEHAHG-LNVRSAYLHLLT-DTLSSVAVVAGGIAIMKWNILWIDPLVTVFISLYIL	188
Thermococcus sp. AM4	254173368	75	RAEILVAFINSAVLIGVSLFLVVEAYRRFK---NPQPIDTGVMLPVALIGLIANLLSVFLLHEHAHG-LNVRSAYLHLLS-DTLSSVAVVIGGLLIRFYGVEWVDPLVTVLIALYIL	186
Thermococcus onnurineus	212223406	78	RAEILVAFVNSAVLVGVALFLLVEAYKRFR---NPNPIDTGLMLVVAVIGLLANLLSVLLLHEHAHESINVRSAYLHLVS-DTLSSVAVVAGGLAIRYYDLVWIDPLVTVFISLYIL	190
Thermotoga petrophila	148269525	69	RSEIIVAFLNSVSIFVVSTLVVIEAVKRLV---NPATVHTSVLLLVSSIGLTANFFSVILLHTHSKESMNVRSAYLHLIA-DTLSSILVVLGAVFMRVWKIYWLDPVLAFVIALYMF	181
Thermotoga sp. RQ2	170288200	69	RSEIIVAFLNSVSIFVVSTLVVIEAVKRLV---NPATVHTSVLLLVSSIGLTANFFSVILLHTHSKESMNVRSTYLHLIA-DTLSSILVVLGAVFMRVWKIYWLDPVLAFVIALYMF	181
Thermotoga maritima	15643304	69	RSEIIVAFLNSVSIFVVSTLVVIEAVKRLL---SPATVHTSVLLLVSSIGLAANFFSVILLHTHSKESMNVRSAYLHLIA-DTLSSILVVLGAVFMRVWKIYWLDPVLAFVIALYMF	181
Thermotoga neapolitana	222099106	69	RSEIIVAFLNSVSIFVVSTLVVIEAVKRLG---NPATVHTSVLLLVSSIGLAANFFSVILLHTHSKESMNVRSAYLHLIA-DTLSSILVVLGAVFMRVWKIYWLDPVLAFVIALYMF	181
			: :: .: .*. : .: . :.* : : : .::: :: .* : . . : :.	
